# Subjective and objective sleep alterations in medication-naïve children and adolescents with autism spectrum disorder: a systematic review and meta-analysis

**DOI:** 10.1017/S2045796023000574

**Published:** 2023-07-20

**Authors:** Heeyeon Kim, Jae Han Kim, Junghwan Kim, Jong Yeob Kim, Samuele Cortese, Lee Smith, Ai Koyanagi, Joaquim Radua, Paolo Fusar-Poli, Andre F. Carvalho, Gonzalo Salazar de Pablo, Jae Il Shin, Keun-Ah Cheon, Marco Solmi

**Affiliations:** 1 Department of Psychiatry, Yongin Severance Hospital, Yonsei University College of Medicine, Yongin, Republic of Korea; 2 Institute of Behavioral Science in Medicine, Yonsei University College of Medicine, Yonsei University Health System, Seoul, Republic of Korea; 3 Yonsei University College of Medicine, Severance Hospital, Yonsei University Health System, Seoul, Republic of Korea; 4 Centre for Innovation in Mental Health, Academic Unit of Psychology, University of Southampton, Southampton, UK; 5 Clinical and Experimental Sciences (CNS and Psychiatry), Faculty of Medicine, University of Southampton, Southampton, UK; 6 Solent NHS Trust, Southampton, UK; 7 Hassenfeld Children's Hospital at NYU Langone, New York University Child Study Center, New York, NY, USA; 8 Centre for Health, Performance, and Wellbeing, Anglia Ruskin University, Cambridge, UK; 9 Parc Sanitari Sant Joan de Déu/CIBERSAM, Universitat de Barcelona, Fundació Sant Joan de Déu, Sant Boi de Llobregat, Barcelona, Spain; 10 Imaging Mood- and Anxiety-Related Disorders (IMARD) Group, Institut d’Investigacions Biomèdiques August Pi i Sunyer(IDIBAPS), Mental Health Research Networking Center(CIBERSAM), University of Barcelona, Barcelona, Spain; 11 Department of Psychosis Studies, Early Psychosis: Interventions and Clinical-detection (EPIC) Lab, Institute of Psychiatry, Psychology & Neuroscience, King’s College London, London, UK; 12 Department of Brain and Behavioral Sciences, University of Pavia, Pavia, Italy; 13 OASIS Service, South London and Maudsley NHS Foundation Trust, London, UK; 14 National Institute for Health Research, Maudsley Biomedical Research Centre, London, UK; 15 IMPACT Strategic Research Centre, Barwon Health, Deakin University School of Medicine, Geelong, VIC, Australia; 16 Department of Child and Adolescent Psychiatry, Institute of Psychiatry and Mental Health, Hospital General Universitario Gregorio Marañón School of Medicine, Universidad Complutense, Instituto de Investigación Sanitaria Gregorio Marañón (IiSGM), CIBERSAM, Madrid, Spain; 17 Child and Adolescent Mental Health Services, South London and Maudsley NHS Foundation Trust, London, UK; 18 Department of Child and Adolescent Psychiatry, Institute of Psychiatry, Psychology and Neuroscience, King’s College London, London, UK; 19 Department of Pediatrics, Yonsei University College of Medicine, Severance Hospital, Yonsei University Health System, Seoul, Republic of Korea; 20 Severance Children's Hospital, Yonsei University Health System, Seoul, Republic of Korea; 21 Severance Underwood Meta-research Center, Institute of Convergence Science, Yonsei University, Seoul, Republic of Korea; 22 Department of Child and Adolescent Psychiatry, Yonsei University College of Medicine, Severance Hospital, Seoul, Republic of Korea; 23 Department of Psychiatry, University of Ottawa, Ottawa, ON, Canada; 24 Department of Mental Health, The Ottawa Hospital, Ottawa, ON, Canada; 25 Ottawa Hospital Research Institute (OHRI) Clinical Epidemiology Program, University of Ottawa, Ottawa, ON, Canada; 26 Department of Child and Adolescent Psychiatry, Charité Universitätsmedizin, Berlin, Germany

**Keywords:** autism spectrum disorder, medication-naïve, meta-analysis, sleep alterations

## Abstract

**Aims:**

This study aimed to summarize the evidence on sleep alterations in medication-naïve children and adolescents with autism spectrum disorder (ASD).

**Methods:**

We systematically searched PubMed/Medline, Embase and Web of Science databases from inception through March 22, 2021. This study was registered with PROSPERO (CRD42021243881). Any observational study was included that enrolled medication-naïve children and adolescents with ASD and compared objective (actigraphy and polysomnography) or subjective sleep parameters with typically developing (TD) counterparts. We extracted relevant data such as the study design and outcome measures. The methodological quality was assessed through the Newcastle-Ottawa Scale (NOS). A meta-analysis was carried out using the random-effects model by pooling effect sizes as Hedges’ *g*. To assess publication bias, Egger’s test and *p*-curve analysis were done. A priori planned meta-regression and subgroup analysis were also performed to identify potential moderators.

**Results:**

Out of 4277 retrieved references, 16 studies were eligible with 981 ASD patients and 1220 TD individuals. The analysis of objective measures showed that medication-naïve ASD patients had significantly longer sleep latency (Hedges’ *g* 0.59; 95% confidence interval [95% CI] 0.26 to 0.92), reduced sleep efficiency (Hedges’ *g* −0.58; 95% CI −0.87 to −0.28), time in bed (Hedges’ *g* −0.64; 95% CI −1.02 to −0.26) and total sleep time (Hedges’ *g* −0.64; 95% CI −1.01 to −0.27). The analysis of subjective measures showed that they had more problems in daytime sleepiness (Hedges’ *g* 0.48; 95% CI 0.26 to 0.71), sleep latency (Hedges’ *g* 1.15; 95% CI 0.72 to 1.58), initiating and maintaining sleep (Hedges’ *g* 0.86; 95% CI 0.39 to 1.33) and sleep hyperhidrosis (Hedges’ *g* 0.48; 95% CI 0.29 to 0.66). Potential publication bias was detected for sleep latency, sleep period time and total sleep time measured by polysomnography. Some sleep alterations were moderated by age, sex and concurrent intellectual disability. The median NOS score was 8 (interquartile range 7.25–8.75).

**Conclusion:**

We found that medication-naïve children and adolescents with ASD presented significantly more subjective and objective sleep alterations compared to TD and identified possible moderators of these differences. Future research requires an analysis of how these sleep alterations are linked to core symptom severity and comorbid behavioural problems, which would provide an integrated therapeutic intervention for ASD. However, our results should be interpreted in light of the potential publication bias.

## Introduction

Autism spectrum disorder (ASD) is a neurodevelopmental disorder characterized by impairment of social communication and repetitive/restrictive behaviours (Lord *et al.*, 2018). Notably, 40–80% of patients with ASD suffer from sleep alterations, which is a significantly higher rate compared with their typically developing (TD) counterparts (Cohen *et al.*, 2014). Sleep alterations in ASD could be partly explained by its underlying neurobiology. Accumulated evidence has supported lower melatonin levels in individuals with ASD, and a previous review found that disturbance of sleep/circadian rhythm was associated with the progression of ASD (Wu *et al.*, 2020). Moreover, the overlaps of sleep maturation and synaptic physiology are believed to play a patho-etiological role in sleep alterations in various neurodevelopmental disorders, including ASD (Crocker and Sehgal, 2010; Gaetz *et al.*, 2014; Veatch *et al.*, 2015; Veenstra-VanderWeele *et al.*, 2012).

Sleep alterations in patients with ASD are associated with core symptoms and problematic behaviours. A recent study reported that short sleep duration showed a strong relationship with patients’ tendency to fail in developing peer relationships (Veatch *et al.*, 2017), which supports the statement that sleep alterations are associated with impairment in social function in ASD (Richdale and Schreck, 2009; Schreck *et al.*, 2004). Internalizing and externalizing behavioural problems have also been found to be strongly correlated with sleep alterations in ASD (Adams *et al.*, 2014; Goldman *et al.*, 2011; Henderson *et al.*, 2011; Mayes and Calhoun, 2009; Schreck *et al.*, 2004; Sikora *et al.*, 2012). Therefore, sleep alterations in these individuals may substantially affect the quality of life of patients and their caregivers (Levin and Scher, 2016).

To appropriately manage these problems, it is necessary to elucidate the characteristics of sleep alterations in children and adolescents with ASD. Numerous primary studies including meta-analysis have investigated sleep alterations in ASD using objective measures such as actigraphy, polysomnography and other subjective measures (Chen *et al.*, 2021; Díaz-Román *et al.*, 2018; Elrod and Hood, 2015). However, previous analyses included studies that enrolled patients with ASD taking psychotropic drugs. Considering that psychotropic drugs have various effects on sleep, their results may not represent the genuine characteristics of sleep alterations in children and adolescents with ASD. In this regard, the present study aims to explore sleep alterations in medication-naïve children and adolescents with ASD compared with TD controls. We meta-analysed each of the various sleep parameters of actigraphy, polysomnography and other subjective measures to (1) accurately elucidate sleep alterations in this population and (2) generate results that could be compared with previous meta-analyses that included studies enrolling patients with ASD prescribed with psychotropic medications.

## Methods

### Protocol, registration and study design

We performed a systematic review and meta-analysis following the Preferred Reporting Items for Systematic Reviews and Meta-analyses guidelines (Supplementary Appendix pp 4–7) (Page *et al.*, 2021). The protocol was registered with PROSPERO (CRD42021243881) with the amendment for addressing reviewers’ comment (Supplementary Appendix p 13). The entire process of screening, data extraction and methodological appraisal of all the included articles was performed by at least two independent authors (HK, JHK and JK).

### Search strategy and selection criteria

**Figure 1.**
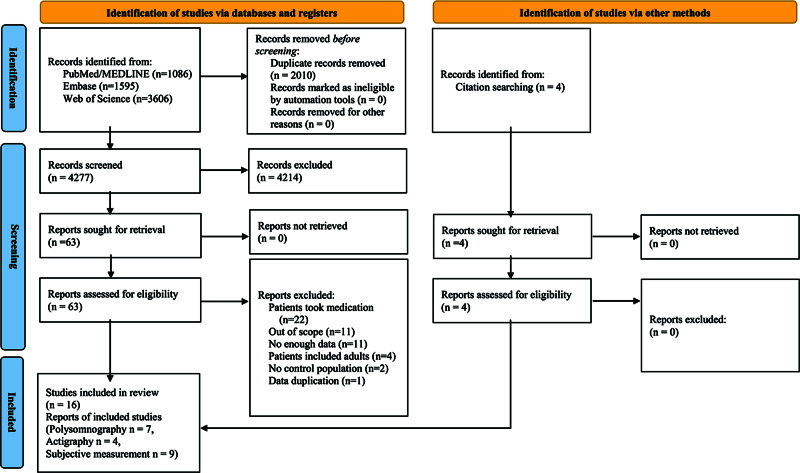
Literature search process.

We systematically searched PubMed/Medline, Embase and Web of Science from database inception to March 22, 2021, without any language restrictions. Full search strategies for each database are presented in Supplementary Appendix p 8. To identify eligible articles, two independent authors (JHK and JK) screened titles, abstracts and full texts sequentially (Fig. 1). We also manually searched references of the relevant studies to find further eligible articles. Any disagreements were resolved by discussion with the other authors (HK, JIS and KAC).

We included studies that met the following inclusion criteria: observational studies that compared the objective (i.e., actigraphy or polysomnography) and/or subjective sleep measures (e.g., Children’s Sleep Habits Questionnaire [CSHQ], Sleep Disturbance Scale for Children [SDSC], and Pediatric Sleep Questionnaire [PSQ]) between ASD patients and TD individuals, studies that clearly stated that patients were medication-naïve and studies that included child or adolescent patients only (age < 18 years). The ASD diagnosis was operationalized according to any version of the International Classification of Diseases, Diagnostic and Statistical Manual of Mental Disorders, Autism Diagnostic Interview or Autism Diagnostic Observation Schedule.

We excluded studies that met the following criteria: studies that did not examine sleep measures in ASD patients, studies that included patients who took medication, studies that did not compare the outcome with TD individuals, studies that did not provide enough data for analysis, studies with intervention, non-human and genetic studies and conference abstracts.

### Data extraction

Two independent authors (JHK and JK) performed data extraction, and any discrepancies were resolved by discussion with the third author (HK). The following data were extracted from each eligible observational study: name of the first author, publication year, the country where the study was conducted, details of the study (number of participants, mean age and corresponding standard deviation [SD], percentage of boys, diagnostic criteria for ASD and comorbidity for intellectual disability), mean and SD for each outcome measure (polysomnography, actigraphy and other subjective measures) and nights recorded for assessment.

### Methodological quality assessment

The quality of eligible articles was assessed independently by two authors (JHK and JK) based on Newcastle-Ottawa Scale (NOS) because all included studies were of a non-randomized design (Wells *et al.*, 2000). The three categories for quality assessment of case–control studies were the selection of the study population, intergroup comparability of case and control groups and the measures of exposure factors. The maximum score of NOS is 9 points. The quality of the study was classified as follows: ‘good’, which required 3 or 4 stars in the selection, 1 or 2 in comparability and 2 or 3 in outcomes; ‘fair’, which required 2 in the selection, 1 or 2 in comparability and 2 or 3 in outcomes; the study was graded ‘poor’ otherwise.

### Statistical analysis

We performed a meta-analysis for each sleep measurement (e.g., sleep latency or time in bed). Specifically, the differences in sleep measures between medication-naïve children and adolescents with ASD and TD controls were converted to standardized effect sizes (Hedges’ *g*) and corresponding 95% confidence intervals (95% CIs) and were combined using random-effects models weighted by the inverse of the study variance and between-study heterogeneity. Additionally, we conducted an influence leave-one-out analysis to examine the effect of each study on the overall estimate by omitting one study at a time.

We performed Cochran’s *Q* test and calculated *I*^2^ statistics to evaluate heterogeneity between studies (Higgins and Thompson, 2002). The *Q* test assesses whether heterogeneity is statistically significant, and *I*^2^ statistics measures the proportion of variance in the pooled effect size attributable to heterogeneity. Publication bias was evaluated using Egger’s test, *p*-curve analysis and visual inspection of the funnel plots (Egger *et al.*, 1997; Simonsohn *et al.*, 2014a, 2014b). The *p*-curve analysis evaluates the presence of evidential value for a set of individual studies. The evidential value was denoted when either of the following condition was satisfied: *P* for the right-skewness test for the half curve was <0.05, or *P* for the right-skewness test for both half and full curve was <0.1. Moreover, we systematically searched observational studies that investigated sleep parameters in medication-naïve ASD patients without TD controls and performed exploratory independent *T*-tests between sleep parameters in two study designs (comparative studies [studies that enrolled both TD and ASD participants] versus non-comparative studies [studies that enrolled ASD participants only]) to gain further insights regarding publication bias. Details are presented in Supplementary Appendix pp 13–29. When potential publication bias was suspected by Egger’s test or *p*-curve analysis, we corrected the effect size using the trim-and-fill method (Duval and Tweedie, 2000).

To evaluate potential moderating factors, we performed meta-regression and subgroup analysis. For continuous variables (publication year, mean age of ASD group and percentage of boys in ASD group), we conducted meta-regression analysis for each sleep measure except those with fewer than four studies. The moderating effects of categorical variables (inclusion of intellectual disability and questionnaires for sleep) were assessed by subgroup analysis. All statistical tests were performed using R version 4.1.0 software and its packages. All statistical tests were two-sided, and statistical significance was set at *P* < 0.05.

## Results

### Study selection and study characteristics

From the database search, we identified 4277 candidate articles after eliminating duplicates, of which 63 were included after the title and abstract screening. We also manually identified four candidate articles from citation screening. After the final screening, 16 studies were eligible for this systematic review and meta-analysis (Fig. 1). The list of included articles is provided in Supplementary Appendix p 8. The list of excluded articles in the full-text screening stage is presented in Supplementary Appendix pp 10–12.

The main characteristics of the eligible studies are presented in Table 1. A total of 981 patients with ASD (median 26.5 per study, interquartile range [IQR] 13–71.75, range 10–414) and 1220 TD individuals (median 27.5 per study, IQR 13–75.75, range 5–584) were included. Out of 16 eligible studies, four studies used actigraphy, seven polysomnography and nine subjective measures (Fig. 1).

**Table 1.** Characteristics of included studies

		ASD group			Control group					
Author (year)	Country	Diagnostic tool	*N* (% boys)	Mean age ± SD (years)	Type	*N* (% boys)	Mean age ± SD (years)	Sleep measures	Nights recorded	Inclusion of intellectual disability
Allik *et al.* (2006)	Sweden	ICD-10	32 (87.5)	10.8 ± 1.24	TD	32 (87.5)	10.9 ± 1.3	ACT and Sleep diary	7	No
Anders *et al.* (2012)	United States	ADOS and ADI-R	68 (81)	3.92 ± 0.83	TD	69 (70)	3.4 ± 0.92	ACT and CSHQ	7	No
Bruni *et al.* (2007)	Italy	ICD-10, DSM-IV, ADOS and CARS	10 (90)	11.9 ± 2.5	TD	12 (58.3)	12.6 ± 3.7	PSG	2	Yes
Elia *et al.* (2000)	Italy	DSM-IV	13 (100)	8.72 ± 2.22	TD	5 (100)	9.22 ± 2.02	PSG	2	No
Harder *et al.* (2016)	United States	ADOS	21 (100)	7.8 ± 1.8	TD	23 (78.3)	8.0 ± 1.9	PSG	1	No
Lambert *et al.* (2016)	Canada	DSM-IV, ADOS and ADI-R	13 (NA)	10.27 ± 2.24	TD	13 (NA)	10.23 ± 2.01	PSG	2	No
Malow *et al.* (2006)	United States	ADOS	11 (81.8)	6.26 ± 1.67	TD	10 (80)	7.62 ± 2.2	PSG and CSHQ	2	No
Miano *et al.* (2007)	Italy	DSM-IV and CARS	16 (100)	9.4 ± 2.33	TD	18 (50)	10.2 ± 2.93	PSG	2	Yes
Mutluer *et al.* (2016)	Turkey	DSM-IV-TR and DSM-5	64 (79.7)	11.66 ± 3.8	TD	53 (75.5)	11.75 ± 0.85	Pediatric sleep questionnaire	NA	Yes
Paavonen *et al.* (2008)	Finland	ICD-10 and DSM-IV	52 (76.9)	10.1 ± 3.4	TD	61 (47.5)	10.0 ± 1.9	SDSC	NA	No
Pace *et al.* (2016)	France	DSM-IV	19 (NA)	10.7 ± 1.2	TD	19 (NA)	9.9 ± 1.6	ACT	7	No
Reynolds *et al.* (2019)	United States	ADOS and ADI-R	414 (NA)	NA	TD	584 (NA)	NA	CSHQ	NA	Yes
Romeo *et al.* (2021)	Italy	DSM-5 and ADOS-2	84 (82.1)	3.9 ± 0.8	TD	84 (77.4)	3.7 ± 0.8	SDSC	NA	Yes
Tessier *et al.* (2015)	Canada	DSM-IV-TR, ADI-R and ADOS	13 (100)	10.23 ± 2.09	TD	13 (100)	10.23 ± 2.01	PSG	2	No
Tse *et al.* (2020)	Hong Kong	DSM-5	78 (79.5)	10.05 ± 1.08	TD	78 (79.5)	10.05 ± 1.08	ACT and Sleep log assessment	7	Yes
Tyagi *et al.* (2019)	India	DSM-5	73 (80.8)	4.8 ± 1.8	TD	146 (80.8)	4.6 ± 1.6	SDSC	NA	No

Abbreviations: ACT = actigraphy, ADI-R = autism diagnostic interview-revised, ADOS = autism diagnostic observation schedule, ASD = autism spectrum disorder, CARS = Childhood Autism Rating Scale, CSHQ = Children’s Sleep Habits Questionnaire, DSM = Diagnostic and Statistical Manual of Mental Disorders, ICD = International Classification of Diseases, *N* = the number, NA = not available, PSQ = polysomnography, SD = standard deviation, SDSC = Sleep Disturbance Scale for Children and TD = typically development.

### Actigraphy

**Figure 2.**
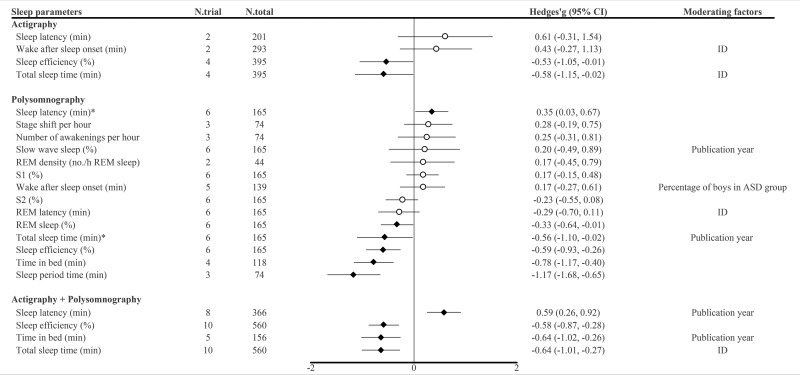
Summary of meta-analysis on objective sleep parameters in medication-naïve autism spectrum disorder.

Abbreviations: ASD = autism spectrum disorder, CI = confidence interval, ID = intellectual disability, NA = not available, N.total = the number of total participants, N.trial = the number of trials, REM = rapid eye movement, S1 = stage 1 sleep and S2 = stage 2 sleep. *Corrected effect size by the trim-and-fill method was presented. †Black rhombus indicated statistical significance, while white circle indicated non-significance.

Among the 16 included articles, four measured sleep parameters with actigraphy. Meta-analyses were performed for sleep efficiency (*n* = 4), sleep latency (*n* = 2), total sleep time (*n* = 4) and wake after sleep onset (*n* = 2). The results showed that the patients with medication-naïve ASD exhibited significantly lower sleep efficiency (Hedges’ *g*, −0.53; 95% CI, −1.05 to −0.01) and total sleep time (Hedges’ *g*, −0.58; 95% CI, −1.15 to −0.02) than TD individuals (Table 2 and Fig. 2). All parameters measured using actigraphy showed large heterogeneity. Subgroup analyses revealed that the effect sizes of total sleep time and wake after sleep onset were moderated by intellectual disability (Table 2, Fig. 2 and Supplementary Appendix p 38).

**Table 2.** Meta-analyses for each sleep alteration of objective sleep measures

	Meta-analysis						Heterogeneity			Egger’s test^a^		*P*-curve analysis^b^		
Sleep parameters	*k*	ASD_N	Control_N	Hedges’ *g* (95% CI)	*Z*	*P*	*Q*	*P*	*I*^2^ (%)	*t*	*P*	Presence of evidential value	Moderating factors	Note^c^
*Actigraphy*														
Sleep efficiency (%)	4	197	198	−0.53 (−1.05, −0.01)	−1.99	<0.05	17.62	0.0005	83	0.554	0.635			c
Sleep latency (min)	2	100	101	0.61 (−0.31, 1.54)	1.31	0.192	8.69	0.0032	88.5					a,b,c,d
Total sleep time (min)	4	197	198	−0.58 (−1.15, −0.02)	−2.02	<0.05	20.31	0.0001	85.2	1.428	0.29		ID	c
Wake after sleep onset (min)	2	146	147	0.43 (−0.27, 1.13)	1.19	0.233	9	0.0027	88.9				ID	a,b,c
*Polysomnography*														
Number of awakenings per hour	3	39	35	0.25 (−0.31, 0.81)	0.88	0.378	2.46	0.2921	18.8	−0.581	0.6649			a,b,c
REM density (no./h REM sleep)	2	26	18	0.17 (−0.45, 0.79)	0.53	0.593	0.77	0.3809	0					a,b,c,d
REM latency (min)	6	84	81	−0.29 (−0.70, 0.11)	−1.42	0.155	7.43	0.1905	32.7	−0.977	0.384		ID	
REM sleep (%)	6	84	81	−0.33 (−0.64, −0.01)	−2.04	<0.05	2.95	0.7069	0	−0.009	0.9931			
S1 (%)	6	84	81	0.17 (−0.15, 0.48)	1.04	0.3	4.83	0.4369	0	−1.652	0.1739			
S2 (%)	6	84	81	−0.23 (−0.55, 0.08)	−1.47	0.141	4.66	0.4593	0	−0.226	0.8323			
Slow wave sleep (%)	6	84	81	0.20 (−0.49, 0.89)	0.58	0.564	19.85	0.0013	74.8	1.223	0.2886		Publication year	
Sleep efficiency (%)	6	84	81	−0.59 (−0.93, −0.26)	−3.49	0.0005	6.09	0.2974	17.9	−1.013	0.3683			
Sleep latency (min)	6	84	81	0.56 (0.24, 0.88)	3.38	<0.001	4.68	0.456	0	3.548	<0.05			
Sleep period time (min)	3	39	35	−1.17 (−1.68, −0.65)	−4.46	<0.0001	0.98	0.6129	0	−3.912	0.1593	No		a,b,c
Stage shift per hour	3	39	35	0.28 (−0.19, 0.75)	1.17	0.244	0.67	0.7136	0	−4.039	0.1545			a,b,c
Time in bed (min)	4	60	58	−0.78 (−1.17, −0.40)	−3.99	<0.0001	2.86	0.4135	0	−3.415	0.0761			
Total sleep time (min)	6	84	81	−0.69 (−1.25, −0.14)	−2.47	<0.05	13.1	0.0224	61.9	−0.857	0.44	No	Publication year	
Wake after sleep onset (min)	5	71	68	0.17 (−0.27, 0.61)	0.75	0.454	6.06	0.1948	34	−1.649	0.1976		Percentage of boys in ASD group	
*Actigraphy + Polysomnography*														
Sleep efficiency (%)	10	281	279	−0.58 (−0.87, −0.28)	−3.81	0.0001	23.72	0.0048	62.1	0.144	0.8888	Yes		
Sleep latency (min)	8	184	182	0.59 (0.26, 0.92)	3.55	<0.0005	13.67	0.0574	48.8	1.812	0.1199		Publication year	
Time in bed (min)	5	79	77	−0.64 (−1.02, −0.26)	−3.3	0.001	5.66	0.226	29.3	−2.661	0.0762		Publication year	
Total sleep time (min)	10	281	279	−0.64 (−1.01, −0.27)	−3.4	<0.001	33.63	0.0001	73.2	0.527	0.6126	Yes	ID	

Abbreviation: ASD = autism spectrum disorder, CI = confidence interval, ID = intellectual disability, *k* = the number of studies, *N* = the number, NA = not available and REM = rapid eye movement.

a Egger’s test may lack the statistical power to detect bias when the number of studies is small (i.e., *k* < 10).

b Blanked cells indicated the case where the *p*-curve analysis was not carried out due to the number of statistically significant studies were scarce.

c Meta-regression or subgroup analysis was not available for (a) publication year, (b) the mean age of ASD group, (c) percentage of boys in ASD group or (d) the inclusion of ID. The reasons for each note are presented in the Supplementary Appendix.

### Polysomnography

Among the 16 included articles, seven measured sleep parameters with polysomnography. Meta-analyses were available for 14 sleep parameters, which were numbers of awakenings per hour (*n* = 3), rapid eye movement (REM) density (*n* = 2), REM latency (*n* = 6), REM sleep (*n* = 6), S1 (*n* = 6), S2 (*n* = 6), slow wave sleep (*n* = 6), sleep efficiency (*n* = 6), sleep latency (*n* = 6), sleep period time (*n* = 3), stage shift per hour (*n* = 3), time in bed (*n* = 4), total sleep time (*n* = 6) and wake after sleep onset (*n* = 5). The results exhibited that patients with medication-naïve ASD showed significantly lower REM sleep (Hedges’ *g*, −0.33; 95% CI, −0.64 to −0.01), sleep efficiency (Hedges’ *g*, −0.59; 95% CI, −0.93 to −0.26), sleep period time (Hedges’ *g*, −1.17; 95% CI, −1.68 to −0.65), time in bed (Hedges’ *g*, −0.78; 95% CI, −1.17 to −0.40), total sleep time (Hedges’ *g*, −0.69; 95% CI, −1.25 to −0.14) and longer sleep latency (Hedges’ *g*, 0.56; 95% CI, 0.24 to 0.88) than TD individuals (Table 2 and Fig. 2). Slow wave sleep and total sleep time showed large heterogeneity. Meta-regression and subgroup analyses showed that the effect size of REM latency, slow wave sleep, total sleep time and wake after sleep onset were moderated by intellectual disability, publication year, publication year and percentage of boys in ASD group, respectively (Table 2, Fig. 2 and Supplementary Appendix pp 33–34, 39).

### Actigraphy + polysomnography

For more concrete evidence, we pooled effect sizes of sleep parameters that were measured using objective sleep measures, and meta-analyses were performed for sleep efficiency (*n* = 10), sleep latency (*n* = 8), time in bed (*n* = 5) and total sleep time (*n* = 10). No study reported outcomes with both actigraphy and polysomnography. The results showed that the patients with medication-naïve ASD had significantly lower sleep efficiency (Hedges’ *g*, −0.58; 95% CI, −0.87 to −0.28), time in bed (Hedges’ *g*, −0.64; 95% CI, −1.02 to −0.26), total sleep time (Hedges’ *g*, −0.64; 95% CI, −1.01 to −0.27) and longer sleep latency (Hedges’ *g*, 0.59; 95% CI, 0.26 to 0.92) than TD individuals (Table 2 and Fig. 2). Sleep efficiency and total sleep time showed large heterogeneity. Meta-regression and subgroup analyses suggested that the effect sizes of sleep latency, time in bed and total sleep time were moderated by publication year, publication year and intellectual disability, respectively (Table 2, Fig. 2 and Supplementary Appendix pp 35, 40).

### Subjective measures

**Figure 3.**
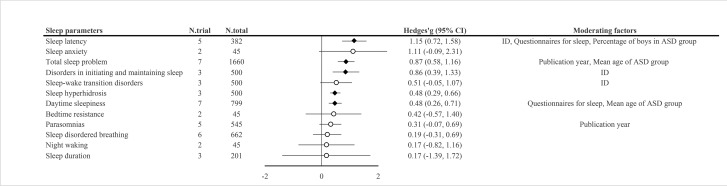
Summary of meta-analysis on subjective sleep parameters in medication-naïve autism spectrum disorder.

Abbreviations: ASD = autism spectrum disorder, CI = confidence interval, ID = intellectual disability, N.total = the number of total participants and N.trial = the number of trials. †Black rhombus indicated statistical significance, while white circle indicated non-significance.

Among the 16 included articles, nine measured sleep problems with subjective measures, which included CSHQ, SDSC, PSQ, sleep diary and sleep log assessment. Since measured sleep problems and their designation varied among sleep measures, we united them according to similarity (Details are in the Supplementary Appendix p 30). Meta-analyses were available for 12 sleep parameters, which were daytime sleepiness (*n* = 7), parasomnias (*n* = 5), sleep-disordered breathing (*n* = 6), sleep latency (*n* = 5), bedtime resistance (*n* = 2), disorders in initiating and maintaining sleep (*n* = 3), night waking (*n* = 2), sleep anxiety (*n* = 2), sleep duration (*n* = 3), sleep hyperhidrosis (*n* = 3), sleep–wake transition disorders (*n* = 3) and total sleep problem (*n* = 7). The results showed that the medication-naïve ASD patients showed significantly increased daytime sleepiness (Hedges’ *g*, 0.48, 0.26 to 0.71), sleep latency (Hedges’ *g*, 1.15; 95% CI, 0.72 to 1.58), disorders in initiating and maintaining sleep (Hedges’ *g*, 0.86; 95% CI, 0.39 to 1.33), sleep hyperhidrosis (Hedges’ *g*, 0.48; 95% CI, 0.29 to 0.66) and total sleep problem (Hedges’ *g*, 0.87; 95% CI, 0.58 to 1.16) compared to TD individuals (Table 3 and Fig. 3). All sleep parameters showed large heterogeneity except for sleep hyperhidrosis.

**Table 3.** Meta-analyses for each sleep problem of subjective sleep measures

	Meta-analysis						Heterogeneity			Egger’s test^a^		*P*-curve analysis^b^		
Sleep parameters	*k*	ASD_N	Control_N	Hedges’ *g* (95% CI)	*Z*	*P*	*Q*	*P*	*I*^2^ (%)	*t*	*P*	Presence of evidential value	Moderating factors	Note^c^
Daytime sleepiness	7	363	436	0.48 (0.26, 0.71)	4.91	<0.0001	11.86	0.0652	49.4	0.749	0.4877	Yes	Mean age of ASD group and Questionnaires for sleep	
Parasomnias	5	231	314	0.31 (−0.07, 0.69)	1.61	0.1075	14.49	<0.01	72.4	1.168	0.3272		Publication year	
Sleep-disordered breathing	6	295	367	0.19 (−0.31, 0.69)	0.74	0.461	48.61	<0.0001	89.7	0.917	0.4108			
Sleep latency	5	196	186	1.15 (0.72, 1.58)	5.22	<0.0001	11.6	0.02	65.5	−1.766	0.1757	Yes	Percentage of boys in ASD group, ID and Questionnaires for sleep	
Bedtime resistance	2	22	23	0.42 (−0.57, 1.40)	0.82	0.41	2.65	0.1037	62.2					a,b,c,d,e
Disorders in initiating and maintaining sleep	3	209	291	0.86 (0.39, 1.33)	3.59	<0.0005	12	0.003	83.3	0.626	0.6441	Yes	ID	a,b,c,e
Night waking	2	22	23	0.17 (−0.82, 1.16)	0.33	0.7398	2.72	0.0992	63.2					a,b,c,d,e
Sleep anxiety	2	22	23	1.11 (−0.09, 2.31)	1.81	0.0696	3.29	0.0696	69.6					a,b,c,d,e
Sleep duration	3	100	101	0.17 (−1.39, 1.72)	0.21	0.8324	21.3	<0.0001	90.6	1.534	0.3678			a,b,c
Sleep hyperhidrosis	3	209	291	0.48 (0.29, 0.66)	5.1	<0.0001	1.84	0.3981	0	9.409	0.0674	Yes		a,b,c,e
Sleep–wake transition disorders	3	209	291	0.51 (−0.05, 1.07)	1.78	0.0751	20.02	<0.0001	90	0.059	0.9625		ID	a,b,c,e
Total sleep problem	7	709	951	0.87 (0.58, 1.16)	5.93	<0.0001	23.88	<0.001	74.9	1.695	0.1509	Yes	Publication year and mean age of ASD group	

Abbreviation: ASD = autism spectrum disorder, CI = confidence interval, ID = intellectual disability, k = the number of studies, N = the number and NA = not available.

a Egger’s test may lack the statistical power to detect bias when the number of studies is small (i.e., *k* < 10).

b Blanked cells indicated the case where the *p*-curve analysis was not carried out due to the number of statistically significant studies were scarce.

c Meta-regression or subgroup analysis was not available for (a) publication year, (b) the mean age of ASD group, (c) percentage of boys in ASD group, (d) the inclusion of ID or (e) questionnaires for sleep. The reasons for each note are presented in the Supplementary Appendix.

Meta-regression and subgroup analysis showed that intellectual disability moderated the effect sizes of sleep latency, disorders in initiating and maintaining sleep and sleep–wake transition disorders; publication year moderated the effect sizes of parasomnias and total sleep problems; the type of questionnaires for assessing sleep moderated the effect sizes of daytime sleepiness and sleep latency; the mean age of the ASD group moderated the effect size of daytime sleepiness and total sleep problems and the percentage of boys in ASD group moderated the effect size of sleep latency (Table 3 and Fig. 3). Detailed results are provided in Supplementary Appendix pp 36–37, 41–42.

### Newcastle-Ottawa scale

We performed the quality assessment of the 16 included studies. The median score was 8, IQR 7.25–8.75 and the range was 6–9. All included studies met the criteria for ‘high’ quality except for two, which were graded as ‘poor’ mainly because they did not provide any factor for comparability between cases and controls. Details of the NOS score for each study with reason are presented in Supplementary Appendix p 31.

### Publication bias assessment and effect size correction

Our Egger’s test and *p*-curve analysis revealed that sleep latency, sleep period time and total sleep time measured by polysomnography might be affected by publication bias (Table 2). We corrected the effect size of sleep latency and total sleep time measured by polysomnography, and statistical significances of both were retained, whereas the effect size was slightly reduced (Table 4). However, the effect size of sleep period time measured by polysomnography was not corrected via the trim-and-fill method possibly because this meta-analysis contained only three individual studies. Our exploratory investigation for publication bias by independent *T*-tests between sleep parameters in two study designs (comparative versus non-comparative studies) suggested that the results from the CSHQ might be suspected to have publication bias. Detailed results are displayed in Supplementary Appendix p 13.

**Table 4.** Correction of the effect sizes by trim-and-fill method

		Meta-analysis				Heterogeneity		
		*k*	Hedges’ *g* (95% CI)	Z	*P*	*Q*	*P*	*I*^2^ (%)
Sleep latency measured by polysomnography	Before correction	6	0.56 (0.24, 0.88)	3.38	<0.001	4.68	0.456	0
	After correction	9^a^	0.35 (0.03, 0.67)	2.13	<0.05	11.97	0.1527	33.1
Total sleep time (min) measured by polysomnography	Before correction	6	−0.69 (−1.25, −0.14)	−2.47	<0.05	13.1	0.0224	61.9
	After correction	7^b^	−0.56 (−1.10, −0.02)	−2.05	<0.05	16.26	0.0124	63.1

Abbreviations: CI = confidence interval and *k* = the number of studies.

a Three studies were added for effect size correction.

b One study was added for effect size correction.

## Discussion

This meta-analysis aimed to explore sleep alterations in medication-naïve children and adolescents with ASD compared with TD individuals. We found that these patients’ group had a variety of sleep issues that were supported by objective and subjective measures. The medication-naïve ASD group had significantly longer sleep latency and lower sleep efficiency, time in bed and total sleep time than the TD group in objective measures combining actigraphy and polysomnography. Polysomnography alone revealed that the medication-naïve ASD group had a lower proportion of REM sleep and shorter sleep period time. According to subjective measures, the medication-naïve ASD group reported increased problems with daytime sleepiness, sleep latency, disorders in initiating and maintaining sleep and sleep hyperhidrosis. Among the more prominent sleep alterations in ASD, we found that daytime sleepiness may become more severe with ageing and that prolonged sleep latency is more evident in girls than in boys. Moreover, moderators of variations in total sleep time and sleep latency were related to concurrent intellectual disability.

Our analysis concerning only objective sleep measures found that the medication-naïve ASD group had more noticeable sleep alterations than TD, which is consistent with a recent meta-analysis that included all ASD patients independent of the drug usage (Chen *et al.*, 2021). However, a lower proportion of REM sleep in children and adolescents with ASD has not been consistently reported in previous research. Considering that REM sleep is most abundant in early life and tends to correlate with the developmental phases of synaptogenesis and cortical plasticity, researchers had attempted to investigate the link between REM sleep and the clinical features of ASD. According to studies that included children with ASD, a lower proportion of REM sleep was correlated with a higher Childhood Autism Rating Scale score (Maski *et al.*, 2013) and Childhood Behaviour Checklist internalizing score (Bruni *et al.*, 2007), which indicated that REM sleep deficiency in ASD is associated with severity of symptoms and behavioural problems. In addition, the cholinergic system was suggested to be a potential contributor to the decreased REM sleep in ASD, considering that acetylcholine is the main driver of REM sleep coordinating REM and non-REM sleep (McCarley, 2007). This may imply the possibility of intervention targeting the cholinergic system in ASD. Indeed, an open-label trial reported that donepezil (acetylcholinesterase inhibitor) could increase REM sleep and improve behavioural problems and inattention in children with ASD (Buckley *et al.*, 2011). However, further research is needed since the evidence is not yet fully established regarding this issue.

Regarding subjective measures, fewer sleep problems were observed compared to the most recent meta-analysis that pooled the results of 39 studies using subjective sleep parameters in the ASD (Díaz-Román *et al.*, 2018). However, caution is needed when interpreting these results. Since we excluded studies that involved ASD patients taking medications, individuals with relatively mild symptoms or lesser comorbidities were selected for this study. Moreover, only nine studies were included in our analysis; thus, a comparatively smaller number of studies reduced the statistical power. Therefore, we should not overlook the potential for genuine sleep problems in ASD patients to be underestimated. For example, while ‘sleep duration’ in subjective parameters did not retain statistical significance, ‘total sleep time’ in objective parameters was supported by concrete evidence.

Moreover, we performed meta-regression and subgroup analysis to identify potential moderating factors. First, among the subjective parameters, the sex of the ASD group was a significant moderator in sleep latency, and our results suggested that sleep latency was more pronounced in girls than in boys. Second, patients’ age was found to be another moderating factor. Our findings suggest that those with ASD experienced more daytime sleepiness and reported more total sleep problems than the TD individuals, and these problems appeared to increase with age. It is noticeable considering that sleep problems usually decrease with age in the normal development (Gregory and O’Connor, 2002; Li *et al.*, 2018). Moreover, since daytime sleepiness in ASD may worsen symptoms such as irritability and thus negatively affect social relationships (Cohen *et al.*, 2014), it may be essential to monitor ASD patients’ complaints of sleep problems during their growth to provide appropriate intervention in time. Third, concurrent intellectual disability has also been discovered to be a moderating factor. Our subgroup analysis reported that the ASD group with intellectual disability had more problems with total sleep time on objective measures and sleep latency on subjective measures than the ASD group without intellectual disability. There are two hypotheses regarding the relationship between sleep problems and intellectual disability in children with ASD. One is that preceding sleep alteration negatively affects cognitive function, and the other is that behavioural problems accompanying intellectual disability may interfere with the current sleep (Elia *et al.*, 2000). However, further studies in this field are required because a small number of studies may lead to overestimation or underestimation, and case–control studies cannot establish a causal relationship.

Our results should be interpreted in light of potential publication bias. Indeed, our Egger’s test and *p*-curve analysis revealed that the results of three sleep parameters (sleep latency, sleep period time and total sleep time measured by polysomnography) were influenced by potential publication bias, and their effect sizes were attenuated after the correction by the trim-and-fill method. This indicated that other sleep parameters might also be susceptible to publication bias, and their effect sizes might be overestimated even if not detected by our Egger’s test and *p*-curve analysis, possibly due to their small number of studies (*k* < 10). Moreover, our further investigation that compared the CSHQ scores between two study designs (comparative studies versus non-comparative studies) suggested that consideration is needed for the possibility of underlying publication bias that originated from the study design (i.e., authors of comparative studies might tend to report more prominent results relative to ASD patients than those in non-comparative studies). However, these analyses were conducted post hoc to address reviewers’ comments, and results are based on few studies and small sample sizes and should be considered exploratory. Therefore, further studies with a non-comparative design and a large number of participants are warranted to establish robust evidence in this field, and until then, careful interpretation with consideration for potential publication bias is required.

### Limitations

This study has some limitations. First, the number of studies and participants included in each meta-analysis was small, resulting in reduced statistical power to identify genuine sleep alterations in ASD patients. This issue became more problematic when subgroup analyses were performed. In this context, we conducted a meta-analysis by combining the results of actigraphy and polysomnography, which contained up to 10 studies with 560 participants, to obtain more reliable evidence. Second, various subjective measures for sleep were united for meta-analysis, which seemed to partly contribute to the large heterogeneity across the results of the subjective measures. However, since we used standardized effect sizes (Hedges’ *g*) and the content between subjective measures was very similar, it appeared to not exert a substantial impact on our results. Third, considering that ASD often requires the administration of psychotropic drugs with various accompanying symptoms, it is possible that the inclusion of medication-naïve patients led to the selection of patients with mild clinical symptoms, which might result in the underestimation of sleep problems in patients with ASD.

## Conclusion

In conclusion, our study performed a meta-analysis that included only medication-naïve children and adolescents to investigate sleep alterations in patients with ASD. Our results suggest that children and adolescents with ASD experience diverse sleep alterations. We also investigated some moderating factors such as the patient’s age, sex and concurrent intellectual disability. Future research should analyse how these sleep alterations are linked to core symptom severity and comorbid behavioural problems to provide an integrated therapeutic intervention for patients with ASD.

## Data Availability

The data used for this study cannot be presented online because included articles are protected by copyright. Additional data from our analysis can be shared by contacting the corresponding author (Keun-Ah Cheon; kacheon@yuhs.ac).

## Supplementary material

For supplementary material accompanying this paper visit http://doi.org/10.1017/S2045796023000574. click here to view supplementary material
