# Supplementary material for: Subjective and objective sleep alterations in medication-naïve children and adolescents with autism spectrum disorder: a systematic review and meta-analysis
Source: Epidemiol Psychiatr Sci. 2023 Jul 20;32:e48. doi: 10.1017/S2045796023000574 (PMC10387490; doi:10.1017/S2045796023000574)
Supplement: Supplementary file 1 [file S2045796023000574sup001.docx]

**Appendix.**

**Subjective and objective sleep alterations in medication-naïve children and adolescents with autism spectrum disorder: a systematic review and meta-analysis**

**[Index]**

[**eAppendix1. PRISMA checklist** 4](#_Toc133321793)

[**Table S1. PRISMA checklist** 4](#_Toc133321794)

[**eAppendix 2. PRISMA abstract checklist** 7](#_Toc133321795)

[**Table S2. PRISMA abstract checklist** 7](#_Toc133321796)

[**eAppendix 3. Full search strategies (The last search was done on March 22^nd^, 2021)** 8](#_Toc133321797)

[**Table S3. Full search strategies** 8](#_Toc133321798)

[**eAppendix 4.** **The list of eligible articles** 9](#_Toc133321799)

[**eAppendix 5. The list of excluded articles by full text screening with exclusion reason** 10](#_Toc133321800)

[**Table S4. The list of excluded articles by full text screening with exclusion reason** 10](#_Toc133321801)

[**eAppendix 6. References of the excluded articles by full text screening** 11](#_Toc133321802)

[**eAppendix 7. Exploratory investigation on publication bias as post-hoc analysis (independent T-tests between sleep parameters in two study designs [comparative studies versus non-comparative studies])** 13](#_Toc133321803)

[**Table S5. Differences in sleep parameters between study designs (comparative versus non-comparative designs)** 13](#_Toc133321804)

[**Figure S1. Study selection flow for further investigation on publication bias (The last search was done on March 22nd, 2021)** 14](#_Toc133321805)

[**eAppendix 7.1 The list of eligible articles** **for further investigation on publication bias** 15](#_Toc133321806)

[**Table S6. The list of excluded articles by full text screening with exclusion reason** **for further investigation on publication bias** 15](#_Toc133321807)

[**eAppendix 8. Unification of sleep parameters assessed by subjective measurements** 30](#_Toc133321808)

[**Table S7. Unification of sleep parameters assessed by subjective measurements** 30](#_Toc133321809)

[**eAppendix 9. Result of the study quality assessment (Newcastle-Ottawa scale)** 31](#_Toc133321810)

[**Table S8. Result of the Newcastle-Ottawa scale** 31](#_Toc133321811)

[**eAppendix 10. Statistical results of meta-regression analyses – Publication year, mean age of ASD group, Percentage of boys in ASD group** 32](#_Toc133321812)

[**Table S9. Statistical results of meta-regression analyses - Actigraphy** 32](#_Toc133321813)

[**Table S10. Statistical results of meta-regression analyses - Polysomnography** 33](#_Toc133321814)

[**Table S10. Continued** 34](#_Toc133321815)

[**Table S11. Statistical results of meta-regression analyses - Actigraphy + Polysomnography** 35](#_Toc133321816)

[**Table S12. Statistical results of meta-regression analyses - Subjective measurements** 36](#_Toc133321817)

[**Table S12. Continued** 37](#_Toc133321818)

[**eAppendix 11. Statistical results of the subgroup analyses - Inclusion of Intellectual disability** 38](#_Toc133321819)

[**Table S13. Statistical results of the subgroup analyses(Inclusion of Intellectual disability) - Actigraphy** 38](#_Toc133321820)

[**Table S14. Statistical results of the subgroup analyses(Inclusion of Intellectual disability) - Polysomnography** 39](#_Toc133321821)

[**Table S15. Statistical results of the subgroup analyses(Inclusion of Intellectual disability) - Actigraphy + Polysomnography** 40](#_Toc133321822)

[**Table S16. Statistical results of the subgroup analyses(Inclusion of Intellectual disability) - Subjective measurements** 41](#_Toc133321823)

[**eAppendix 12. Statistical results of the subgroup analyses - Questionnaires for sleep (Subjective measurement)** 42](#_Toc133321824)

[**eAppendix 13. Funnel plot, p-curve analysis plot, and influence analysis plot** 43](#_Toc133321825)

[**13.1. Actigraphy** 43](#_Toc133321826)

[**Figure S2. Funnel plot and influence analysis plot for Sleep efficiency (%)** 43](#_Toc133321827)

[**Figure S3. Funnel plot for Sleep latency(min)** 43](#_Toc133321828)

[**Figure S4. Funnel plot and influence analysis plot for Total sleep time (min)** 43](#_Toc133321829)

[**Figure S5. Funnel plot for Wake after sleep onset (min)** 44](#_Toc133321830)

[**13.2. Polysomnography** 44](#_Toc133321831)

[**Figure S6. Funnel plot and influence analysis plot for Number of awakenings per hour** 44](#_Toc133321832)

[**Figure S7. Funnel plot for REM density (no./h REM sleep)** 44](#_Toc133321833)

[**Figure S8. Funnel plot and influence analysis plot for REM latency (min)** 45](#_Toc133321834)

[**Figure S9. Funnel plot and influence analysis plot for REM sleep (%)** 45](#_Toc133321835)

[**Figure S10. Funnel plot and influence analysis plot for S1 (%)** 45](#_Toc133321836)

[**Figure S11. Funnel plot and influence analysis plot for S2 (%)** 46](#_Toc133321837)

[**Figure S12. Funnel plot and influence analysis plot for Slow wave sleep (%)** 46](#_Toc133321838)

[**Figure S13. Funnel plot and influence analysis plot for Sleep efficiency (%)** 46](#_Toc133321839)

[**Figure S14. Funnel plot and influence analysis plot for Sleep latency (min)** 47](#_Toc133321840)

[**Figure S15. Funnel plot, p-curve plot, and influence analysis plot for Sleep period time (min)** 47](#_Toc133321841)

[**Figure S16. Funnel plot and influence analysis plot for Stage shift per hour** 48](#_Toc133321842)

[**Figure S17. Funnel plot and influence analysis plot for Time in bed (min)** 48](#_Toc133321843)

[**Figure S18. Funnel plot, p-curve plot, and influence analysis plot for Total sleep time (min)** 49](#_Toc133321844)

[**Figure S19. Funnel plot and influence analysis plot for Wake after sleep onset (min)** 49](#_Toc133321845)

[**13.3. Actigraphy + Polysomnography** 50](#_Toc133321846)

[**Figure S20. Funnel plot, p-curve plot, and influence analysis plot for Sleep efficiency** 50](#_Toc133321847)

[**Figure S21. Funnel plot and influence analysis plot for Sleep latency (min)** 50](#_Toc133321848)

[**Figure S22. Funnel plot and influence analysis plot for Time in bed (min)** 51](#_Toc133321849)

[**Figure S23. Funnel plot, p-curve plot, and influence analysis plot for Total sleep time (min)** 51](#_Toc133321850)

[**13.4. Subjective measurement** 52](#_Toc133321851)

[**Figure S24. Funnel plot, and p-curve plot, and influence analysis plot for Daytime sleepiness** 52](#_Toc133321852)

[**Figure S25. Funnel plot and influence analysis plot for Parasomnias** 52](#_Toc133321853)

[**Figure S26. Funnel plot and influence analysis plot for Sleep disordered breathing** 53](#_Toc133321854)

[**Figure S27. Funnel plot, p-curve plot, and influence analysis plot for Sleep latency** 53](#_Toc133321855)

[**Figure S28. Funnel plot for Bedtime resistance** 54](#_Toc133321856)

[**Figure S29. Funnel plot, and p-curve plot, and influence analysis plot for Disorders in initiating and maintaining sleep** 54](#_Toc133321857)

[**Figure S30. Funnel plot for Night waking** 55](#_Toc133321858)

[**Figure S31. Funnel plot for Sleep anxiety** 55](#_Toc133321859)

[**Figure S32. Funnel plot and influence analysis plot for Sleep duration** 55](#_Toc133321860)

[**Figure S33. Funnel plot, and p-curve plot, and influence analysis plot for Sleep hyperhidrosis** 56](#_Toc133321861)

[**Figure S34. Funnel plot and influence analysis plot for Sleep-wake transition disorders** 56](#_Toc133321862)

[**Figure S35. Funnel plot, and p-curve plot, and influence analysis plot for Total sleep problem** 57](#_Toc133321863)

# **eAppendix1. PRISMA checklist**

## **Table S1. PRISMA checklist**

| **Section and Topic** | **Item #** | **Checklist item** | **Location where item is reported** |
| --- | --- | --- | --- |
| **TITLE** | | |  |
| Title | 1 | Identify the report as a systematic review. | Title page |
| **ABSTRACT** | | |  |
| Abstract | 2 | See the PRISMA 2020 for Abstracts checklist. | Appendix p 6 |
| **INTRODUCTION** | | |  |
| Rationale | 3 | Describe the rationale for the review in the context of existing knowledge. | #2 |
| Objectives | 4 | Provide an explicit statement of the objective(s) or question(s) the review addresses. | #2 |
| **METHODS** | | |  |
| Eligibility criteria | 5 | Specify the inclusion and exclusion criteria for the review and how studies were grouped for the syntheses. | #3 |
| Information sources | 6 | Specify all databases, registers, websites, organisations, reference lists and other sources searched or consulted to identify studies. Specify the date when each source was last searched or consulted. | #3 |
| Search strategy | 7 | Present the full search strategies for all databases, registers and websites, including any filters and limits used. | Appendix p 8 |
| Selection process | 8 | Specify the methods used to decide whether a study met the inclusion criteria of the review, including how many reviewers screened each record and each report retrieved, whether they worked independently, and if applicable, details of automation tools used in the process. | Figure 1, #5 |
| Data collection process | 9 | Specify the methods used to collect data from reports, including how many reviewers collected data from each report, whether they worked independently, any processes for obtaining or confirming data from study investigators, and if applicable, details of automation tools used in the process. | #3 |
| Data items | 10a | List and define all outcomes for which data were sought. Specify whether all results that were compatible with each outcome domain in each study were sought (e.g. for all measures, time points, analyses), and if not, the methods used to decide which results to collect. | #3 |
|  | 10b | List and define all other variables for which data were sought (e.g. participant and intervention characteristics, funding sources). Describe any assumptions made about any missing or unclear information. | #3 |
| Study risk of bias assessment | 11 | Specify the methods used to assess risk of bias in the included studies, including details of the tool(s) used, how many reviewers assessed each study and whether they worked independently, and if applicable, details of automation tools used in the process. | #3-4 |
| Effect measures | 12 | Specify for each outcome the effect measure(s) (e.g. risk ratio, mean difference) used in the synthesis or presentation of results. | #4 |
| Synthesis methods | 13a | Describe the processes used to decide which studies were eligible for each synthesis (e.g. tabulating the study intervention characteristics and comparing against the planned groups for each synthesis (item #5)). | #4 |
|  | 13b | Describe any methods required to prepare the data for presentation or synthesis, such as handling of missing summary statistics, or data conversions. | #4 |
|  | 13c | Describe any methods used to tabulate or visually display results of individual studies and syntheses. | #4 |
|  | 13d | Describe any methods used to synthesize results and provide a rationale for the choice(s). If meta-analysis was performed, describe the model(s), method(s) to identify the presence and extent of statistical heterogeneity, and software package(s) used. | #4 |
|  | 13e | Describe any methods used to explore possible causes of heterogeneity among study results (e.g. subgroup analysis, meta-regression). | #4 |
|  | 13f | Describe any sensitivity analyses conducted to assess robustness of the synthesized results. | #4 |
| Reporting bias assessment | 14 | Describe any methods used to assess risk of bias due to missing results in a synthesis (arising from reporting biases). | #3-4 |
| Certainty assessment | 15 | Describe any methods used to assess certainty (or confidence) in the body of evidence for an outcome. | Not applicable |
| **RESULTS** | | |  |
| Study selection | 16a | Describe the results of the search and selection process, from the number of records identified in the search to the number of studies included in the review, ideally using a flow diagram. | Figure 1, #5 |
|  | 16b | Cite studies that might appear to meet the inclusion criteria, but which were excluded, and explain why they were excluded. | Appendix pp 10-12 |
| Study characteristics | 17 | Cite each included study and present its characteristics. | Table 1, Appendix p 9 |
| Risk of bias in studies | 18 | Present assessments of risk of bias for each included study. | #6 |
| Results of individual studies | 19 | For all outcomes, present, for each study: (a) summary statistics for each group (where appropriate) and (b) an effect estimate and its precision (e.g. confidence/credible interval), ideally using structured tables or plots. | Table 1 |
| Results of syntheses | 20a | For each synthesis, briefly summarise the characteristics and risk of bias among contributing studies. | #5-6 |
|  | 20b | Present results of all statistical syntheses conducted. If meta-analysis was done, present for each the summary estimate and its precision (e.g. confidence/credible interval) and measures of statistical heterogeneity. If comparing groups, describe the direction of the effect. | #5-6 |
|  | 20c | Present results of all investigations of possible causes of heterogeneity among study results. | #6-7 |
|  | 20d | Present results of all sensitivity analyses conducted to assess the robustness of the synthesized results. | #5-6 |
| Reporting biases | 21 | Present assessments of risk of bias due to missing results (arising from reporting biases) for each synthesis assessed. | #6 |
| Certainty of evidence | 22 | Present assessments of certainty (or confidence) in the body of evidence for each outcome assessed. | Not applicable |
| **DISCUSSION** | | |  |
| Discussion | 23a | Provide a general interpretation of the results in the context of other evidence. | #8 |
|  | 23b | Discuss any limitations of the evidence included in the review. | #9-10 |
|  | 23c | Discuss any limitations of the review processes used. | #9-10 |
|  | 23d | Discuss implications of the results for practice, policy, and future research. | #8-9 |
| **OTHER INFORMATION** | | |  |
| Registration and protocol | 24a | Provide registration information for the review, including register name and registration number, or state that the review was not registered. | #3 |
|  | 24b | Indicate where the review protocol can be accessed, or state that a protocol was not prepared. | #3 |
|  | 24c | Describe and explain any amendments to information provided at registration or in the protocol. | #3, Appendix p 13 |
| Support | 25 | Describe sources of financial or non-financial support for the review, and the role of the funders or sponsors in the review. | #11 |
| Competing interests | 26 | Declare any competing interests of review authors. | #11 |
| Availability of data, code and other materials | 27 | Report which of the following are publicly available and where they can be found: template data collection forms; data extracted from included studies; data used for all analyses; analytic code; any other materials used in the review. | #11 |

| **Section and Topic** | **Item #** | **Checklist item** | **Reported (Yes/No)** |
| --- | --- | --- | --- |
| **TITLE** | | |  |
| Title | 1 | Identify the report as a systematic review. | Yes |
| **BACKGROUND** | | |  |
| Objectives | 2 | Provide an explicit statement of the main objective(s) or question(s) the review addresses. | Yes |
| **METHODS** | | |  |
| Eligibility criteria | 3 | Specify the inclusion and exclusion criteria for the review. | Yes |
| Information sources | 4 | Specify the information sources (e.g. databases, registers) used to identify studies and the date when each was last searched. | Yes |
| Risk of bias | 5 | Specify the methods used to assess risk of bias in the included studies. | Yes |
| Synthesis of results | 6 | Specify the methods used to present and synthesise results. | Yes |
| **RESULTS** | | |  |
| Included studies | 7 | Give the total number of included studies and participants and summarise relevant characteristics of studies. | Yes |
| Synthesis of results | 8 | Present results for main outcomes, preferably indicating the number of included studies and participants for each. If meta-analysis was done, report the summary estimate and confidence/credible interval. If comparing groups, indicate the direction of the effect (i.e. which group is favoured). | Yes |
| **DISCUSSION** | | |  |
| Limitations of evidence | 9 | Provide a brief summary of the limitations of the evidence included in the review (e.g. study risk of bias, inconsistency and imprecision). | Yes |
| Interpretation | 10 | Provide a general interpretation of the results and important implications. | Yes |
| **OTHER** | | |  |
| Funding | 11 | Specify the primary source of funding for the review. | Not applicable |
| Registration | 12 | Provide the register name and registration number. | Yes |

# **eAppendix 2. PRISMA abstract checklist**

## **Table S2. PRISMA abstract checklist**

# **eAppendix 3. Full search strategies (The last search was done on March 22^nd^, 2021)**

## **Table S3. Full search strategies**

| PubMed (1086 articles were found) |
| --- |
| ("autis*" OR "Asperg*" OR "pervasive developmental disorder") AND (sleep [tiab] OR sleepiness [tiab] OR alertness [tiab] OR vigilance [tiab]) AND (child [tiab] OR children [tiab] OR adolescent* OR pediatric OR paediatric OR young people [tiab]) |
| Web of science (1595 articles were found) |
| (autis* OR Asperg* OR (pervasive developmental disorder)) AND (sleep OR sleepiness OR alertness OR vigilance) AND (child OR children OR adolescent* OR pediatric OR paediatric OR young people) |
| Embase (3606 articles were found) |
| (autis* OR Asperg* OR (pervasive developmental disorder)) AND (sleep OR sleepiness OR alertness OR vigilance) AND (child OR children OR adolescent* OR pediatric OR paediatric OR young people) |

# **eAppendix 4.** **The list of eligible articles**

**Allik H, Larsson J O and Smedje H** (2006) Sleep patterns of school-age children with Asperger syndrome or high-functioning autism. *Journal of Autism and Developmental Disorders*. **36**, 585-595.

**Anders T, Iosif A M, Schwichtenberg A J, Tang K and Goodlin-Jones B** (2012) Sleep and Daytime Functioning: A Short-term Longitudinal Study of Three Preschool-age Comparison Groups. *Ajidd-American Journal on Intellectual and Developmental Disabilities*. **117**, 275-290.

**Bruni O, Ferri R, Vittori E, Novelli L, Vignati M, Porfirio M C, Arico D, Bernabei P and Curatolo P** (2007) Sleep architecture and NREM alterations in children and adolescents with Asperger syndrome. *Sleep*. **30**, 1577-1585.

**Elia M, Ferri R, Musumeci S A, Del Gracco S, Bottitta M, Scuderi C, Miano G, Panerai S, Bertrand T and Grubar J C** (2000) Sleep in subjects with autistic disorder: a neurophysiological and psychological study. *Brain & Development*. **22**, 88-92.

**Harder R, Malow B A, Goodpaster R L, Iqbal F, Halbower A, Goldman S E, Fawkes D B, Wang L, Shi Y P, Baudenbacher F and Diedrich A** (2016) Heart rate variability during sleep in children with autism spectrum disorder. *Clinical Autonomic Research*. **26**, 423-432.

**Lambert A, Tessier S, Rochette A C, Scherzer P, Mottron L and Godbout R** (2016) Poor sleep affects daytime functioning in typically developing and autistic children not complaining of sleep problems: A questionnaire-based and polysomnographic study. *Research in Autism Spectrum Disorders*. **23**, 94-106.

**Malow B A, Marzec M L, McGrew S G, Wang L, Henderson L M and Stone W L** (2006) Characterizing sleep in children with autism spectrum disorders: A multidimensional approach. *Sleep*. **29**, 1563-1571.

**Miano S, Bruni V, Elia M, Trovato A, Smerieri A, Verrillo E, Roccella M, Terzano M G and Ferri R** (2007) Sleep in children with autistic spectrum disorder: A questionnaire and polysomnographic study. *Sleep Medicine*. **9**, 64-70.

**Mutluer T, Demirkaya S K and Abali O** (2016) Assessment of Sleep Problems and Related Risk Factors Observed in Turkish Children with Autism Spectrum Disorders. *Autism Research*. **9**, 536-542.

**Paavonen E J, Vehkalahti K, Vanhala R, Von Wendt L, Wendt T N V and Aronen E T** (2008) Sleep in children with Asperger syndrome. *Journal of Autism and Developmental Disorders*. **38**, 41-51.

**Pace M, Dumortier L, Favre-Juvin A, Guinot M and Bricout V A** (2016) Heart rate variability during sleep in children with autism spectrum disorders. *Physiology & Behavior*. **167**, 309-312.

**Reynolds A M, Soke G N, Sabourin K R, Hepburn S, Katz T, Wiggins L D, Schieve L A and Levy S E** (2019) Sleep Problems in 2-to 5-Year-Olds With Autism Spectrum Disorder and Other Developmental Delays. *Pediatrics*. **143**, 9.

**Romeo D M, Brogna C, Belli A, Lucibello S, Cutrona C, Apicella M, Mercuri E and Mariotti P** (2021) Sleep Disorders in Autism Spectrum Disorder Pre-School Children: An Evaluation Using the Sleep Disturbance Scale for Children. *Medicina-Lithuania*. **57**, 10.

**Tessier S, Lambert A, Scherzer P, Jemel B and Godbout R** (2015) REM sleep and emotional face memory in typically-developing children and children with autism. *Biological Psychology*. **110**, 107-114.

**Tse A C Y, Yu C C W and Lee P H** (2020) Comparing sleep patterns between children with autism spectrum disorder and children with typical development: A matched case-control study. *Autism*. **24**, 2298-2303.

**Tyagi V, Juneja M and Jain R** (2019) Sleep Problems and Their Correlates in Children with Autism Spectrum Disorder: An Indian Study. *Journal of Autism and Developmental Disorders*. **49**, 1169-1181.

# **eAppendix 5. The list of excluded articles by full text screening with exclusion reason**

## **Table S4. The list of excluded articles by full text screening with exclusion reason**

| **Author, year** | **Reason for exclusion** |
| --- | --- |
| Lehoux, et al. 2019 | data duplicate with Lambert et al. 2016 |
| Samanta, et al. 2020 | no control |
| Yavuz-Kodat, et al. 2020 | no control |
| Bartakovičová et al, 2019 | no data |
| Best et al, 2018 | no data |
| Delemere & Dounavi 2018 | no data |
| Gagnon et al, 2018 | no data |
| Gupta et al, 2020 | no data |
| Kuhlthau et al, 2018 | no data |
| Lawson et al, 2020 | no data |
| Malhi et al, 2019 | no data |
| McLay et al, 2020 | no data |
| Uren et al, 2019 | no data |
| Winkelman et al, 2018 | no data |
| Souders et al, 2009 | no medication naïve patient presented |
| Al-Farsi et al, 2019 | no medication-naïve patient presented |
| Alder et al, 2020 | no medication-naïve patient presented |
| Arazi et al, 2020 | no medication-naïve patient presented |
| Benson et al, 2019 | no medication-naïve patient presented |
| Cebreros-Paniagua et al, 2020 | no medication-naïve patient presented |
| Halstead et al, 2021 | no medication-naïve patient presented |
| Harris et al, 2018 | no medication-naïve patient presented |
| Holingue et al, 2021 | no medication-naïve patient presented |
| Johnson et al, 2018 | no medication-naïve patient presented |
| Koo et al, 2020 | no medication-naïve patient presented |
| Lindor et al, 2019 | no medication-naïve patient presented |
| Neumeyer et al, 2019 | no medication-naïve patient presented |
| Phung et al, 2019 | no medication-naïve patient presented |
| Restrepo et al, 2020 | no medication-naïve patient presented |
| Richdale & Schreck, 2019 | no medication-naïve patient presented |
| Shui et al, 2021 | no medication-naïve patient presented |
| Surtees et al, 2019 | no medication-naïve patient presented |
| Taylor & Siegel, 2019 | no medication-naïve patient presented |
| Taylor et al, 2018 | no medication-naïve patient presented |
| Yavuz-Kodat et al, 2019 | no medication-naïve patient presented |
| Zwaigenbaum et al, 2020 | no medication-naïve patient presented |
| Jeon et al, 2020 | out of scope |
| Johansson et al, 2018 | out of scope |
| Knuppel et al, 2018 | out of scope |
| McCrae et al, 2018 | out of scope |
| McGovney et al, 2020 | out of scope |
| Richdale & Roussis, 2018 | out of scope |
| Smidt et al, 2019 | out of scope |
| Tomkies et al, 2018 | out of scope |
| Tomkies et al, 2019 | out of scope |
| Toper, 2018 | out of scope |
| Vite et al, 2018 | out of scope |
| Baker et al, 2019 | studies for adult with autism spectrum disorder |
| Ballester et al, 2019 | studies for adult with autism spectrum disorder |
| Deserno et al, 2019 | studies for adult with autism spectrum disorder |
| Goldman et al, 2017 | studies for adult with autism spectrum disorder |

# **eAppendix 6. References of the excluded articles by full text screening**

**Al-Farsi O A, Al-Farsi Y M, Al-Sharbati M M and Al-Adawi S** (2019) Sleep habits and sleep disorders among children with autism spectrum disorders, intellectual disabilities and typically developing children in Oman: a case-control study. *Early Child Development and Care*. **189**, 2370-2380.

**Alder M L, Ye F, Run F, Bagai K, Fawkes D B, Peterson B T and Malow B A** (2020) Application of a novel actigraphy algorithm to detect movement and sleep/wake patterns in children with autism spectrum disorder. *Sleep Medicine*. **71**, 28-34.

**Arazi A, Meiri G, Danan D, Michaelovski A, Flusser H, Menashe I, Tarasiuk A and Dinstein I** (2020) Reduced sleep pressure in young children with autism. *Sleep*. **43**, 11.

**Baker E K, Richdale A L, Hazi A and Prendergast L A** (2019) Assessing a hyperarousal hypothesis of insomnia in adults with autism spectrum disorder. *Autism Research*. **12**, 897-910.

**Ballester P, Martínez M J, Javaloyes A, Inda M d M, Fernández N, Gázquez P, Aguilar V, Pérez A, Hernández L and Richdale A L** (2019) Sleep problems in adults with autism spectrum disorder and intellectual disability. *Autism Research*. **12**, 66-79.

**Bartakovičová K, Keményová P, Siklenková L, Ostatníková D and Babinská K** (2019) Sleep disturbances in children with autism spectrum disorder. *Act Nerv Super Rediviva*. **61**, 41-48.

**Benson S, Bender A M, Wickenheiser H, Naylor A, Clarke M, Samuels C H and Werthner P** (2019) Differences in sleep patterns, sleepiness, and physical activity levels between young adults with autism spectrum disorder and typically developing controls. *Developmental neurorehabilitation*. **22**, 164-173.

**Best V, Riedel A, Feige B, Tebartz van Elst L, Riemann D and Spiegelhalder K** (2018) Subjektive schlafbezogene Parameter bei Patienten mit Autismus-Spektrum-Störung. *Somnologie*. **22**, 262-266.

**Cebreros-Paniagua R, Ayala-Guerrero F and Mateos-Salgado E L** (2020) Sleep disturbances in patients with Asperger syndrome related to the severity of their symptoms. *Sleep and Vigilance*. **4**, 161-166.

**Delemere E and Dounavi K** (2018) Parent-implemented bedtime fading and positive routines for children with autism spectrum disorders. *Journal of Autism and Developmental Disorders*. **48**, 1002-1019.

**Deserno M K, Borsboom D, Begeer S, Agelink Van Rentergem J A, Mataw K and Geurts H M** (2019) Sleep determines quality of life in autistic adults: A longitudinal study. *Autism Research*. **12**, 794-801.

**Gagnon K, Lambert A, Tessier S, Rochette A C and Godbout R** (2018) Association between REM sleep EEG connectivity and daytime core symptoms in children with Autism Spectrum Disorder. *Journal of Sleep Research*. **27**, 2.

**Goldman S, Alder M, Burgess H, Corbett B, Hundley R, Wofford D, Fawkes D, Wang L, Laudenslager M and Malow B** (2017) Characterizing sleep in adolescents and adults with autism spectrum disorders. *Journal of Autism and Developmental Disorders*. **47**, 1682-1695.

**Gupta A, Shukla G, Poornima S, Mohd A, Katoch J, Taneja D and Singhal N** (2020) 0969 early life sleep disturbance among children with autism spectrum disorders: a questionnaire-based retrospective study. *Sleep*. **43**, A368.

**Halstead E J, Joyce A, Sullivan E, Tywyn C, Davies K, Jones A and Dimitriou D** (2021) Sleep disturbances and patterns in children with neurodevelopmental conditions. *Frontiers in pediatrics*. **9**, 91.

**Harris J, Malow B and Werkhaven J** (2018) 0787 Descriptive Epidemiology of Obstructive Sleep Apnea in Children with Autism Spectrum Disorder. *Sleep*. **41**, A292.

**Holingue C, Volk H, Crocetti D, Gottlieb B, Spira A P and Mostofsky S H** (2021) Links between parent-reported measures of poor sleep and executive function in childhood autism and attention deficit hyperactivity disorder. *Sleep Health*. **7**, 375-383.

**Jeon M, Halstead E J and Dimitriou D** (2020) Cross-cultural comparison of sleep patterns of typically developing children and children with autism spectrum disorder in the UK and South Korea. *Journal of Sleep Research*. **29**, 347-347.

**Johansson A E E, Feeley C A, Dorman J S and Chasens E R** (2018) Characteristics of sleep in children with autism spectrum disorders from the Simons Simplex Collection. *Research in Autism Spectrum Disorders*. **53**, 18-30.

**Johnson C R, Smith T, DeMand A, Lecavalier L, Evans V, Gurka M, Swiezy N, Bearss K and Scahill L** (2018) Exploring sleep quality of young children with autism spectrum disorder and disruptive behaviors. *Sleep Medicine*. **44**, 61-66.

**Knuppel A, Telleus G K, Jakobsen H and Lauritsen M B** (2018) Quality of life in adolescents and adults with autism spectrum disorder: Results from a nationwide Danish survey using self reports and parental proxy-reports. *Research in Developmental Disabilities*. **83**, 247-259.

**Koo H W, Ismail J, Yang W W and Zakaria S Z S** (2021) Sleep Disturbances in Children With Autism Spectrum Disorder at a Malaysian Tertiary Hospital. *Frontiers in pediatrics*. **8**, 7.

**Kuhlthau K A, McDonnell E, Coury D L, Payakachat N and Macklin E** (2018) Associations of quality of life with health-related characteristics among children with autism. *Autism*. **22**, 804-813.

**Lawson L P, Richdale A L, Haschek A, Flower R L, Vartuli J, Arnold S R and Trollor J N** (2020) Cross-sectional and longitudinal predictors of quality of life in autistic individuals from adolescence to adulthood: The role of mental health and sleep quality. *Autism*. **24**, 954-967.

**Lehoux T, Carrier J and Godbout R** (2019) NREM sleep EEG slow waves in autistic and typically developing children: Morphological characteristics and scalp distribution. *Journal of Sleep Research*. **28**, e12775.

**Lindor E, Sivaratnam C, May T, Stefanac N, Howells K and Rinehart N** (2019) Problem behavior in autism spectrum disorder: considering core symptom severity and accompanying sleep disturbance. *Frontiers in psychiatry*, 487.

**Malhi P, Kaur A, Singhi P and Sankhyan N** (2019) Sleep dysfunction and behavioral daytime problems in children with autism spectrum disorders: A comparative study. *The Indian Journal of Pediatrics*. **86**, 12-17.

**McCrae C S, Chan W S, Deroche C B, Munoz M, McLean D, Davenport M, Muckerman J, Takahashi N, McCann D, McGovney K, Sahota P and Mazurek M** (2018) CBT FOR INSOMNIA IN CHILDREN WITH AUTISM SPECTRUM DISORDER (ASD). *Sleep*. **41**, A298-A298.

**McGovney K D, Curtis A F, Mazurek M, Chan W S, Deroche C B, Munoz M, Davenport M, Takamatsu S, Takahashi N, Muckerman J, McCann D, Sahota P, Mills B and McCrae C S** (2020) NIGHTLY ASSOCIATIONS BETWEEN PRE-BEDTIME ACTIVITY, ACTIGRAPHIC LIGHT, AND SLEEP IN CHILDREN WITH ASD AND INSOMNIA. *Sleep*. **43**, A350-A351.

**McLay L, Hansen S G, Carnett A, France K G and Blampied N M** (2020) Attributions, causal beliefs, and help-seeking behavior of parents of children with autism spectrum disorder and sleep problems. *Autism*. **24**, 1829-1840.

**Neumeyer A M, Anixt J, Chan J, Perrin J M, Murray D, Coury D L, Bennett A, Farmer J and Parker R A** (2019) Identifying associations among co-occurring medical conditions in children with autism spectrum disorders. *Academic Pediatrics*. **19**, 300-306.

**Phung J N, Abdullah M M and Goldberg W A** (2019) Poor sleep quality among adolescents with ASD is associated with depressive symptoms, problem behaviors, and conflicted family relationships. *Focus on Autism and Other Developmental Disabilities*. **34**, 173-182.

**Restrepo B, Angkustsiri K, Taylor S L, Rogers S J, Cabral J, Heath B, Hechtman A, Solomon M, Ashwood P and Amaral D G** (2020) Developmental–behavioral profiles in children with autism spectrum disorder and co‐occurring gastrointestinal symptoms. *Autism Research*. **13**, 1778-1789.

**Richdale A and Roussis S** (2018) Sleep problem severity and behaviour in children with autism aged 2-to 5-years. *Journal of Sleep Research*. **27**, 2.

**Richdale A L and Schreck K A** (2019) Examining sleep hygiene factors and sleep in young children with It and without autism spectrum disorder. *Research in Autism Spectrum Disorders*. **57**, 154-162.

**Samanta P, Mishra D P, Panigrahi A, Mishra J, Senapati L K and Ravan J R** (2020) Sleep disturbances and associated factors among 2-6-year-old male children with autism in Bhubaneswar, India. *Sleep Medicine*. **67**, 77-82.

**Shui A M, Lampinen L A, Zheng S T and Katz T** (2021) Characteristics associated with parental estimates of sleep duration in children with autism spectrum disorders. *Research in Autism Spectrum Disorders*. **80**, 15.

**Smidt S E, Ghorai A, Gehringer B, Dow H, Griffiths Z, Taylor S, Zhang J, Rader D, Almasy L, Brodkin E and Bucan M** (2019) Sleep in Autism Spectrum Disorder Without Intellectual Disability. *Neuropsychopharmacology*. **44**, 188-189.

**Souders M C, Mason T B, Valladares O, Bucan M, Levy S E, Mandell D S, Weaver T E and Pinto-Martin J** (2009) Sleep behaviors and sleep quality in children with autism spectrum disorders. *Sleep*. **32**, 1566-1578.

**Surtees A D, Richards C, Clarkson E L, Heald M, Trickett J, Denyer H, Crawford H and Oliver C** (2019) Sleep problems in autism spectrum disorders: A comparison to sleep in typically developing children using actigraphy, diaries and questionnaires. *Research in Autism Spectrum Disorders*. **67**, 101439.

**Taylor B, Palka T, Grados M, Peura C, Verdi M and Siegel M** (2018) 0841 Discrepancies between Parent-Reported and Observed Sleep Disturbance in Hospitalized Children with Autism Spectrum Disorder (ASD). *Sleep*. **41**, A312-A312.

**Taylor B J and Siegel M** (2019) 0783 Characterization Of Sleep Disturbance Profiles In Hospitalized Children With Autism Spectrum Disorder. *Sleep*. **42**, A314-A315.

**Tomkies A, Johnson R F and Mitchell R B** (2018) Obstructive sleep apnea in children with autism spectrum disorder. *Otolaryngology - Head and Neck Surgery (United States)*. **159**, P308.

**Tomkies A, Johnson R F, Shah G, Caraballo M, Evans P and Mitchell R B** (2019) Obstructive Sleep Apnea in Children With Autism. *Journal of Clinical Sleep Medicine*. **15**, 1469-1476.

**Toper O** (2018) Sleep Problems and Behavioral Interventions for Children With Autism Spectrum Disorders and Intellectual Disabilities. *Ankara Universitesi Egitim Bilimleri Fakultesi Ozel Egitim Dergisi-Ankara University Faculty of Educational Sciences Journal of Special Education*. **19**, 801-824.

**Uren J, Richdale A L, Cotton S M and Whitehouse A J** (2019) Sleep problems and anxiety from 2 to 8 years and the influence of autistic traits: a longitudinal study. *European Child & Adolescent Psychiatry*. **28**, 1117-1127.

**Vite T K G, Guerrero F A, Salgado E L M and Paniagua R C** (2018) Characterization of the Mu rhythm during the sleep of children with autism spectrum disorder level 1. *Salud Mental*. **41**, 109-116.

**Winkelman T, Naples A, Rolison M, McNaughton K, Day T, Hasselmo S, McAllister T, Ellison K, Jarzabek E and Lewis B** (2018) Children with Autism Demonstrate Atypical Resting EEG Correlates of Sleepiness. *Age (years)*. **9**, 15.

**Yavuz-Kodat E, Reynaud E, Geoffray M-M, Limousin N, Franco P, Bonnet-Brilhault F, Bourgin P and Schroder C M** (2020) Disturbances of continuous sleep and circadian rhythms account for behavioral difficulties in children with autism spectrum disorder. *Journal of Clinical Medicine*. **9**, 1978.

**Yavuz-Kodat E, Reynaud E, Geoffray M-M, Limousin N, Franco P, Bourgin P and Schroder C M** (2019) Validity of actigraphy compared to polysomnography for sleep assessment in children with autism spectrum disorder. *Frontiers in psychiatry*, 551.

**Zwaigenbaum L, Zaidman-Zait A, Duku E, Bennett T, Mirenda P, Smith I, Szatmari P, Vaillancourt T, Waddell C, Elsabbagh M, Georgiades S, Kerns C and Ungar W** (2020) Profiles of sleep problems among young children with autism spectrum disorders. *Paediatrics and Child Health (Canada)*. **25**, e23.

# **eAppendix 7. Exploratory investigation on publication bias as post-hoc analysis (independent T-tests between sleep parameters in two study designs [comparative studies versus non-comparative studies])**

In the main text, we addressed comparative studies only that compared sleep parameters between patients with autism spectrum disorder (ASD) and typically developing (TD) individuals. However, this concept could potentially make our results susceptible to publication bias since comparative studies may have the motivation to overrate ASD patients' sleep parameters compared to TD controls to publish positive results. Herein, we exploratory evaluated publication bias using the following process as a post-hoc analysis.

*Methods*

We performed an additional systematic search to identify observational studies that investigated sleep parameters in medication-naive ASD patients without controls under the age of 18. We systematically searched PubMed/Medline, Embase, and Web of Science from database inception to March 22, 2021, without any language restrictions. We used the original search strategies since they were appropriate to detect these studies (Appendix p 8). The study selection process that included title, abstract, and full-text screening was independently performed by two first authors (JHK and JK), and disagreement on this process was resolved by discussion.

After the screening process, data extraction was performed by two independent authors (JHK and JK). The following data were extracted for analysis: name of the first author, publication year, the country where the study was conducted, details of the study (number of participants, mean age and corresponding standard deviation [SD], diagnostic criteria for ASD), mean and SD for each outcome measure.

We performed independent T-tests for each sleep parameter to assess the differences between study designs (i.e., comparative studies [originally included in this study] versus non-comparative studies [newly identified study]). We also conducted F-tests before T-tests to assess the equality of two variances with the null hypothesis that the variances of two groups are equal. F-test was the only appropriate test to investigate the homogeneity of variance since we only had summarized results. The normality of each group was assumed because both the normality test and the Wilcoxon rank sum test were impossible to conduct. According to the results of F-tests, Student's T-tests (if the null hypothesis was not rejected) or Welch's T-tests (if the null hypothesis was rejected) were done. All statistical tests were performed using R version 4.1.0 software and its packages. All statistical tests were two-sided and statistical significance was set at P < 0.05.

*Results*

Among the 4277 citations that were found from a systematic search, 292 survived after the title and abstract screening. However, after the full-text screening, only one study was found to be eligible (Samanta et al. 2020), which reported the scores of the Children’s Sleep Habits Questionnaire (CSHQ) only. As a result of independent T-tests, there were statistically significant differences in the following CSHQ sleep parameters: bedtime resistance, sleep latency, sleep duration, sleep anxiety, and total sleep problem (see below table).

## **Table S5. Differences in sleep parameters between study designs (comparative versus non-comparative designs)**

|  | k | N | Comparative design (mean ± SD) | k | N | Non-comparative design (mean ± SD) | *P* |
| --- | --- | --- | --- | --- | --- | --- | --- |
| Bedtime resistance | 2 | 22 | 8.25 ± 8.92 | 1 | 100 | 16.05 ± 2.57 | <0.0001^a^ |
| Sleep latency (min) | 2 | 22 | 115.38 ± 58.12 | 1 | 100 | 153 ± 41.4 | <0.01^a^ |
| Sleep duration (min) | 2 | 22 | 279.62 ± 228.96 | 1 | 100 | 426.6 ± 115.2 | <0.01^a^ |
| Sleep anxiety | 2 | 22 | 6.56 ± 7.02 | 1 | 100 | 9.8 ± 2.12 | <0.05^a^ |
| Night wakings | 2 | 22 | 4.34 ± 6.37 | 1 | 100 | 5.69 ± 1.83 | 0.335^a^ |
| Sleep disordered breathing | 2 | 22 | 3.67 ± 3.65 | 1 | 100 | 5.06 ± 2.29 | 0.099^a^ |
| Daytime sleepiness | 3 | 90 | 13.09 ± 1.68 | 1 | 100 | 12.84 ± 4.36 | 0.61^b^ |
| Parasomnias | 2 | 22 | 9.3 ± 9.14 | 1 | 100 | 12.96 ± 4.4 | 0.08^a^ |
| Total sleep problem | 3 | 436 | 48.85 ± 3.19 | 1 | 100 | 66.86 ± 14.77 | <0.0001^a^ |
| Abbreviations: k=the number of studies, N=the number of patients with autism spectrum disorder, SD=standard deviation  ^a^ Welch's T-test was done since equality of two variances could not be assumed by the result of F-test.  ^b^ Student's T-test was done since equality of two variances could be assumed by the result of F-test. | | | | | | | |

## **Figure S1. Study selection flow for further investigation on publication bias (The last search was done on March 22nd, 2021)**

**Identification of studies via databases and registers**

Records identified from:

PubMed/MEDLINE (n=1086)

Embase (n=1595)

Web of Science (n=3606)

Records removed *before screening*:

Duplicate records removed

(n = 2010)

Records marked as ineligible by automation tools (n = 0)

Records removed for other reasons (n = 0)

**Identification**

Records excluded

(n = 3985)

Reports sought for retrieval

(n =292)

Reports assessed for eligibility

(n = 292)

Records screened

(n = 4277)

Studies included in review

(n = 1)

Reports of included studies

(Subjective measurement : n = 1)

**Screening**

Reports not retrieved

(n = 0)

291 reports excluded:

Out of scope (n=83)

Not enough data (n=75)

Patients took medication (n=73)

Comparative design (n=29)

Review article (n=12)

Not observational study (n=9)

Data duplication (n=6)

Patients included adults (n=4)

**Included**

## **eAppendix 7.1 The list of eligible articles** **for further investigation on publication bias**

1. Samanta, P., Mishra, D. P., Panigrahi, A., Mishra, J., Senapati, L. K., & Ravan, J. R. (2020). Sleep disturbances and associated factors among 2-6-year-old male children with autism in Bhubaneswar, India. *Sleep medicine*, *67*, 77–82.

## **Table S6. The list of excluded articles by full text screening with exclusion reason** **for further investigation on publication bias**

| **Reference** | **Reason for exclusion** |
| --- | --- |
| Baker, E. K., Richdale, A. L., Hazi, A., & Prendergast, L. A. (2019). Assessing a hyperarousal hypothesis of insomnia in adults with autism spectrum disorder. *Autism Research, 12*(6), 897-910. | Patients included adults |
| Ballester, P., Martínez, M. J., Javaloyes, A., Inda, M.-d.-M., Fernández, N., Gázquez, P., . . . Peiró, A. M. (2019). Sleep problems in adults with autism spectrum disorder and intellectual disability. Autism Research, 12(1), 66-79. | Patients included adults |
| Deserno, M. K., Borsboom, D., Begeer, S., Agelink Van Rentergem, J. A., Mataw, K., & Geurts, H. M. (2019). Sleep determines quality of life in autistic adults: A longitudinal study. *Autism Research, 12*(5), 794-801. | Patients included adults |
| Goldman, S., Alder, M., Burgess, H., Corbett, B., Hundley, R., Wofford, D., . . . Malow, B. (2017). Characterizing sleep in adolescents and adults with autism spectrum disorders. *Journal of Autism and Developmental Disorders, 47*, 1682-1695. | Patients included adults |
| Allik, H., Larsson, J.-O., & Smedje, H. (2006). Sleep patterns of school-age children with Asperger syndrome or high-functioning autism. *Journal of Autism and Developmental Disorders, 36*, 585-595. | Comparative design |
| Anders, T., Iosif, A.-M., Schwichtenberg, A., Tang, K., & Goodlin-Jones, B. (2012). Sleep and daytime functioning: a short-term longitudinal study of three preschool-age comparison groups. *American Journal on Intellectual and Developmental Disabilities, 117*(4), 275-290. | Comparative design |
| Best, V., Riedel, A., Feige, B., Tebartz van Elst, L., Riemann, D., & Spiegelhalder, K. (2018). Subjective sleep-related parameters in patients with autism spectrum disorders. *Somnologie, 22*, 262-266. | Comparative design |
| Bruni, O., Ferri, R., Vittori, E., Novelli, L., Vignati, M., Porfirio, M. C., . . . Curatolo, P. (2007). Sleep architecture and NREM alterations in children and adolescents with Asperger syndrome. *Sleep, 30*(11), 1577-1585. | Comparative design |
| Delahaye, J., Kovacs, E., Sikora, D., Hall, T. A., Orlich, F., Clemons, T. E., . . . Kuhlthau, K. (2014). The relationship between health-related quality of life and sleep problems in children with autism spectrum disorders. *Research in autism spectrum disorders, 8*(3), 292-303. | Comparative design |
| Diomedi, M., Curatolo, P., Scalise, A., Placidi, F., Caretto, F., & Gigli, G. L. (1999). Sleep abnormalities in mentally retarded autistic subjects: Down's syndrome with mental retardation and normal subjects. *Brain and Development, 21*(8), 548-553. | Comparative design |
| Eyuboglu, M., & Eyuboglu, D. (2018). Sensory reactivity and sleep problems in toddlers with autism spectrum disorder and anxiety/depression symptoms in their mothers: are they related? *Early Child Development and Care*. | Comparative design |
| Fletcher, F. E., Knowland, V., Walker, S., Gaskell, M. G., Norbury, C., & Henderson, L. M. (2020). Atypicalities in sleep and semantic consolidation in autism. *Developmental Science, 23*(3), e12906. | Comparative design |
| Giannotti, F., Cortesi, F., Cerquiglini, A., & Bernabei, P. (2006). An open-label study of controlled-release melatonin in treatment of sleep disorders in children with autism. *Journal of Autism and Developmental Disorders, 36*, 741-752. | Comparative design |
| Goldman, S. E., Surdyka, K., Cuevas, R., Adkins, K., Wang, L., & Malow, B. A. (2009). Defining the sleep phenotype in children with autism. *Developmental neuropsychology, 34*(5), 560-573. | Comparative design |
| Guler, S., Yesil, G., Ozdil, M., Ekici, B., & Onal, H. (2016). Sleep disturbances and serum vitamin D levels in children with autism spectrum disorder. *Int. J. Clin. Exp. Med, 9*, 14691-14697. | Comparative design |
| Hering, E., Epstein, R., Elroy, S., Iancu, D. R., & Zelnik, N. (1999). Sleep patterns in autistic children. *Journal of Autism & Developmental Disorders, 29*(2). | Comparative design |
| Hirata, I., Mohri, I., Kato-Nishimura, K., Tachibana, M., Kuwada, A., Kagitani-Shimono, K., . . . Taniike, M. (2016). Sleep problems are more frequent and associated with problematic behaviors in preschoolers with autism spectrum disorder. *Research in developmental disabilities, 49*, 86-99. | Comparative design |
| Humphreys, J. S., Gringras, P., Blair, P. S., Scott, N., Henderson, J., Fleming, P. J., & Emond, A. M. (2014). Sleep patterns in children with autistic spectrum disorders: a prospective cohort study. *Archives of disease in childhood, 99*(2), 114-118. | Comparative design |
| Jeon, M., Halstead, E., & Dimitriou, D. (2020). *Cross-cultural comparison of sleep patterns of typically developing children and children with autism spectrum disorder in the UK and South Korea.* Paper presented at the Journal of Sleep Research. | Comparative design |
| Lambert, A., Tessier, S., Rochette, A.-C., Scherzer, P., Mottron, L., & Godbout, R. (2016). Poor sleep affects daytime functioning in typically developing and autistic children not complaining of sleep problems: A questionnaire-based and polysomnographic study. *Research in autism spectrum disorders, 23*, 94-106. | Comparative design |
| Lawson, L. P., Richdale, A. L., Haschek, A., Flower, R. L., Vartuli, J., Arnold, S. R., & Trollor, J. N. (2020). Cross-sectional and longitudinal predictors of quality of life in autistic individuals from adolescence to adulthood: The role of mental health and sleep quality. *Autism, 24*(4), 954-967. | Comparative design |
| Lehoux, T., Carrier, J., & Godbout, R. (2019). NREM sleep EEG slow waves in autistic and typically developing children: morphological characteristics and scalp distribution. *Journal of Sleep Research, 28*(4), e12775. | Comparative design |
| Malhi, P., Kaur, A., Singhi, P., & Sankhyan, N. (2019). Sleep dysfunction and behavioral daytime problems in children with autism spectrum disorders: a comparative study. *The Indian Journal of Pediatrics, 86*, 12-17. | Comparative design |
| Malow, B. A., Marzec, M. L., McGrew, S. G., Wang, L., Henderson, L. M., & Stone, W. L. (2006). Characterizing sleep in children with autism spectrum disorders: a multidimensional approach. *Sleep, 29*(12), 1563-1571. | Comparative design |
| Miano, S., Bruni, O., Elia, M., Trovato, A., Smerieri, A., Verrillo, E., . . . Ferri, R. (2007). Sleep in children with autistic spectrum disorder: a questionnaire and polysomnographic study. *Sleep medicine, 9*(1), 64-70. | Comparative design |
| Mutluer, T., Karakoc Demirkaya, S., & Abali, O. (2016). Assessment of sleep problems and related risk factors observed in T urkish children with A utism spectrum disorders. *Autism Research, 9*(5), 536-542. | Comparative design |
| Paavonen, E. J., Vehkalahti, K., Vanhala, R., von Wendt, L., Nieminen-von Wendt, T., & Aronen, E. T. (2008). Sleep in children with Asperger syndrome. *Journal of Autism and Developmental Disorders, 38*, 41-51. | Comparative design |
| Reynolds, A. M., Soke, G. N., Sabourin, K. R., Hepburn, S., Katz, T., Wiggins, L. D., . . . Levy, S. E. (2019). Sleep problems in 2-to 5-year-olds with autism spectrum disorder and other developmental delays. *Pediatrics, 143*(3). | Comparative design |
| Richdale, A. L., & Prior, M. R. (1995). The sleep/wake rhythm in children with autism. *European child & adolescent psychiatry, 4*, 175-186. | Comparative design |
| Romeo, D. M., Brogna, C., Belli, A., Lucibello, S., Cutrona, C., Apicella, M., . . . Mariotti, P. (2021). Sleep disorders in autism spectrum disorder pre-school children: an evaluation using the sleep disturbance scale for children. *Medicina, 57*(2), 95. | Comparative design |
| Tse, A. C., Yu, C., & Lee, P. H. (2020). Comparing sleep patterns between children with autism spectrum disorder and children with typical development: A matched case–control study. *Autism, 24*(8), 2298-2303. | Comparative design |
| Tyagi, V., Juneja, M., & Jain, R. (2019). Sleep problems and their correlates in children with autism spectrum disorder: An Indian study. *Journal of Autism and Developmental Disorders, 49*, 1169-1181. | Comparative design |
| Wang, G., Liu, Z., Xu, G., Jiang, F., Lu, N., Baylor, A., & Owens, J. (2016). Sleep disturbances and associated factors in Chinese children with autism spectrum disorder: a retrospective and cross-sectional study. *Child Psychiatry & Human Development, 47*, 248-258. | Comparative design |
| Delemere, E., & Dounavi, K. (2018). Parent-implemented bedtime fading and positive routines for children with autism spectrum disorders. *Journal of Autism and Developmental Disorders, 48*, 1002-1019. | Not observational study |
| Johnson, C. R., Turner, K. S., Foldes, E., Brooks, M. M., Kronk, R., & Wiggs, L. (2013). Behavioral parent training to address sleep disturbances in young children with autism spectrum disorder: a pilot trial. *Sleep medicine, 14*(10), 995-1004. | Not observational study |
| Moon, E. C., Corkum, P., & Smith, I. M. (2011). Case study: A case-series evaluation of a behavioral sleep intervention for three children with autism and primary insomnia. *Journal of pediatric psychology, 36*(1), 47-54. doi:10.1093/jpepsy/jsq057 | Not observational study |
| Weiskop, S., Matthews, J., & Richdale, A. (2001). Treatment of sleep problems in a 5-year-old boy with autism using behavioural principles. *Autism, 5*(2), 209-221. | Not observational study |
| Garstang, J., & Wallis, M. (2006). Randomized controlled trial of melatonin for children with autistic spectrum disorders and sleep problems. *Child: care, health and development, 32*(5), 585-589. | Not observational study |
| Goldman, S. E., Adkins, K. W., Calcutt, M. W., Carter, M. D., Goodpaster, R. L., Wang, L., . . . Malow, B. A. (2014). Melatonin in children with autism spectrum disorders: endogenous and pharmacokinetic profiles in relation to sleep. *Journal of Autism and Developmental Disorders, 44*, 2525-2535. | Not observational study |
| Malow, B., Adkins, K. W., McGrew, S. G., Wang, L., Goldman, S. E., Fawkes, D., & Burnette, C. (2012). Melatonin for sleep in children with autism: a controlled trial examining dose, tolerability, and outcomes. *Journal of Autism and Developmental Disorders, 42*, 1729-1737. | Not observational study |
| Paavonen, E. J., Nieminen-von Wendt, T., Vanhala, R., Aronen, E. T., & von Wendt, L. (2003). Effectiveness of melatonin in the treatment of sleep disturbances in children with Asperger disorder. *Journal of Child and Adolescent Psychopharmacology, 13*(1), 83-95. | Not observational study |
| Wright, B., Sims, D., Smart, S., Alwazeer, A., Alderson-Day, B., Allgar, V., . . . Jardine, J. (2011). Melatonin versus placebo in children with autism spectrum conditions and severe sleep problems not amenable to behaviour management strategies: a randomised controlled crossover trial. *Journal of Autism and Developmental Disorders, 41*, 175-184. | Not observational study |
| Malow, B., Adkins, K., Clemons, T., Goldman, S., Molloy, C., Wofford, D., . . . Surdyka, K. (2011). *Effects of a standardized pamphlet on sleep latency in children with autism.* Paper presented at the Sleep. | Data duplication |
| McLay, L., France, K., Blampied, N., & Hunter, J. (2019). Using functional behavioral assessment to treat sleep problems in two children with autism and vocal stereotypy. *International Journal of Developmental Disabilities, 65*(3), 175-184. | Data duplication |
| Tomkies, A., Johnson, R. F., Shah, G., Caraballo, M., Evans, P., & Mitchell, R. B. (2019). Obstructive sleep apnea in children with autism. *Journal of Clinical Sleep Medicine, 15*(10), 1469-1476. | Data duplication |
| Wirojanan, J., Jacquemont, S., Diaz, R., Bacalman, S., Anders, T. F., Hagerman, R. J., & Goodlin-Jones, B. L. (2009). The efficacy of melatonin for sleep problems in children with autism, fragile X syndrome, or autism and fragile X syndrome. *Journal of Clinical Sleep Medicine, 5*(2), 145-150. | Data duplication |
| Yavuz-Kodat, E., Reynaud, E., Geoffray, M.-M., Limousin, N., Franco, P., Bonnet-Brilhault, F., . . . Schroder, C. M. (2020). Disturbances of continuous sleep and circadian rhythms account for behavioral difficulties in children with autism spectrum disorder. *Journal of clinical medicine, 9*(6), 1978. | Data duplication |
| Yavuz-Kodat, E., Reynaud, E., Geoffray, M.-M., Limousin, N., Franco, P., Bonnet-Brilhault, F., . . . Schroder, C. M. (2020). Disturbances of continuous sleep and circadian rhythms account for behavioral difficulties in children with autism spectrum disorder. *Journal of clinical medicine, 9*(6), 1978. | Data duplication |
| Abel, E., Schwichtenberg, A., & Brodhead, M. (2017). 0916 assessing sleep problems within the context of early behavioral intervention for autism spectrum disorder. *Sleep, 40*, A340-A341. | Not enough data |
| Aguilar Andujar, M., Sanchez, G., Menendez De Leon, C., Ramos, I., & Marquez, A. (2014). *Sleep characteristics in children with autism spectrum disorder.* Paper presented at the Journal of Sleep Research. | Not enough data |
| Ayala-Guerrero, F., Mateos, E. L., Villamar, C., García-Vite, T., Alcántara, M., & Gutierrez, C. A. (2019). The patient sleep of the autista spectrum and theory of the mind. Sleep Science, 12, 18. Retrieved from https://www.embase.com/search/results?subaction=viewrecord&id=L630029412&from=export | Not enough data |
| Ballester, P., Martinez Madrid, M., Canet, T., Richdale, A., & Peiro, A. (2020). *Sleep problems across a lifespan of children, adolescents and adults with autism spectrum disorder and intellectual disability at a glance.* Paper presented at the Journal of Sleep Research. | Not enough data |
| Bartakovicova, K., Kemenyova, P., Siklenkova, L., Ostatnikova, D., & Babinska, K. (2019). Sleep disturbances in children with autism spectrum disorder. *Activitas Nervosa Superior Rediviva, 61*(2), 41-48. Retrieved from <Go to ISI>://WOS:000489092000001 | Not enough data |
| Bisson, F., & Golfeto, J. H. (2000). Sleep disorders in children with autism. Infanto, 8(2), 90-93. | Not enough data |
| Bouvier, M., Claustrat, B., & Franco, P. (2012). *Melatonin treatment in autism spectrum disorders (ASD): preliminary results.* Paper presented at the Journal of Sleep Research. | Not enough data |
| Calhoun, S., Durica, K., Fernandez-Mendoza, J., Pearl, A., He, F., & Mayes, S. (2016). Sleep disturbance, working memory and learning problems in adolescents with high functioning autism spectrum disorder. *Journal of Sleep Research, 25*, 282-283. doi:10.1111/jsr.12446 | Not enough data |
| Cao, M., Luo, J., Shi, B., & Jing, J. (2020). *Parent-reported sleep onset latency is associated with increased salivary cortisol level among autism boys.* Paper presented at the Journal of Sleep Research. | Not enough data |
| Chen, X., Velez, J., Barbosa, C., Pepper, M., Gelaye, B., Carskadon, M. A., . . . Williams, M. A. (2015). Actigraphy-measured sleep patterns among children with autism and other disabilities. Sleep, 38, A381. | Not enough data |
| Cohen, S., Cornish, K., Rajaratnam, S., Lockley, S., & Conduit, R. (2014). Relationship between sleep deficiency and poor behaviour in children with autism spectrum disorder. *Sleep and Biological Rhythms, 12*, 37. doi:10.1111/sbr.12082 | Not enough data |
| Dimitriou, D., & Pavlopoulou, G. (2019). Autistic teens personal accounts about their sleep problems and daytime anxiety. *Sleep medicine, 64*, S92-S93. | Not enough data |
| Elkhatib Smidt, S., Ghorai, A., Gehringer, B., Dow, H., Smernoff, Z., Taylor, S., . . . Brodkin, E. (2020). 0974 Family-Based Study Of Sleep In Autism Spectrum Disorder Without Intellectual Disability. *Sleep, 43*(Supplement_1), A370-A370. | Not enough data |
| Elkhatib, S., Lu, F., Rao, S., Asato, A., & Handen, B. (2017). *Socioeconomic Status and Sleep in Children with Autism Spectrum Disorder.* Paper presented at the ANNALS OF NEUROLOGY. | Not enough data |
| Ellis, J., & Aldridge, D. (2012). Attention Biases in Children with Autism Spectrum Disorders and Sleep Problems. | Not enough data |
| Fidan, T., Asfuroglu, B., & Yanardag, M. (2019). THE effect of strucured pyhsical activity on sleep and mental health in severe autistic children. | Not enough data |
| Findling, R. L., Gringras, P., Nir, T., & Zisapel, N. (2017). *Short-and Long-Term Prolonged Release Melatonin Treatment for Sleep Disorders in Children With Autism Spectrum Disorders–Results of a Phase III Randomized Clinical Trial.* Paper presented at the 64th Annual Meeting. | Not enough data |
| Gagnon, K., Lambert, A., Tessier, S., Rochette, A.-C., & Godbout, R. (2018). *Association between REM sleep EEG connectivity and daytime core symptoms in children with Autism Spectrum Disorder.* Paper presented at the Journal of Sleep Research. | Not enough data |
| Galán, C., Sánchez, S., Franco, L., Bravo, R., Rivero, M., Rodríguez, A. B., & Barriga, C. (2017). Tryptophan-enriched antioxidant cereals improve sleep in children with autistic spectrum and attention deficit hyperactivity disorders. *Journal of Cellular Neuroscience and Oxidative Stress, 9*(1), 608-616. | Not enough data |
| Garcia-Gomez, A., Morillo, M. M., Sanchez, J. C. Z., & Cotrina, L. J. (2020). Physical activity and sleep in a group of three teenagers with autism. *Retos-Nuevas Tendencias En Educacion Fisica Deporte Y Recreacion*(38), 248-254. | Not enough data |
| Geranmayeh, A., Holding, B., Lundmark, I., Fondberg, R., & Axelsson, J. (2018). *Sleep and daytime functioning in children with autism.* Paper presented at the Journal of Sleep Research. | Not enough data |
| Goldman, S. E., McGrew, S., Johnson, K. P., Richdale, A. L., Clemons, T., & Malow, B. A. (2011). Sleep is associated with problem behaviors in children and adolescents with autism spectrum disorders. *Research in autism spectrum disorders, 5*(3), 1223-1229. | Not enough data |
| Goldman, S., Clemons, T., McGrew, S., & Malow, B. (2010). *SPECTRUM OF SLEEP PROBLEMS IN CHILDREN WITH AUTISM-AN EPIDEMIOLOGICAL ANALYSIS.* Paper presented at the Sleep. | Not enough data |
| Gringras, P., Findling, R., Nir, T., & Zisapel, N. (2017). Short and long term prolonged release melatonin treatment for sleep disorders in children with autism spectrum disorders: results of a phase III randomized clinical trial. *Sleep medicine, 40*, e119. | Not enough data |
| Gupta, A., Shukla, G., Poornima, S., Mohammed, A., Katoch, J., Taneja, D., & Singhal, N. (2019). 0782 Circadian Rhythm Sleep Disorders And Their Predictors Among Children With Autism-A Prospective Case Control Study. *Sleep, 42*, A314. | Not enough data |
| Gupta, A., Shukla, G., Poornima, S., Mohd, A., Katoch, J., Taneja, D., & Singhal, N. (2020). 0969 early life sleep disturbance among children with autism spectrum disorders: a questionnaire-based retrospective study. *Sleep, 43*, A368. | Not enough data |
| Holsboer-Trachsler, E. (2015). *Aerobic Exercise and Skill Training Improved Objective Sleep and Motor Skills in Children Suffering from Autism Spectrum Disorder (ASD)-A Pilot Study.* Paper presented at the BIOLOGICAL PSYCHIATRY. | Not enough data |
| Hori, R., Suda, M., Sasanabe, R., & Shiomi, T. (2019a). *AUTISM TENDENCY OF PATIENTS WITH SUSPECTED HYPERSOMNIA.* Paper presented at the PSYCHOSOMATIC MEDICINE. | Not enough data |
| Hori, R., Suda, M., Sasanabe, R., & Shiomi, T. (2019b). *Autism tendency of patients with symptoms of hypersomnia.* Paper presented at the PSYCHOTHERAPY AND PSYCHOSOMATICS. | Not enough data |
| Jaworski, M., Chicoine, M., Martello, E., Godbout, R., & Belhumeur, C. (2016). Development of A Novel Tool, The Owl-Sleep Inventory, for the Assessment of Sleep Disorders in Children With Comorbid Psychiatric Illness. *Paediatr Child Health, 21*(Supplement_5), e66-e66. | Not enough data |
| Jmor, S., & Abbas, E. (2015). G202 (P) Sleep management in autistic spectrum disorder. In: BMJ Publishing Group Ltd. | Not enough data |
| Katz, T., Malow, B. A., Shui, A., Carno, M., Connolly, H. V., Coury, D., & Bennett, A. (2013). Medication use in children with autism spectrum disorders and sleep difficulties. Sleep, 36, A379. | Not enough data |
| Lawson, L. M., & Little, L. (2017). Feasibility of a swimming intervention to improve sleep behaviors of children with autism spectrum disorder. *Therapeutic Recreation Journal, 51*(2). | Not enough data |
| Leu, R. M., Beyderman, L., Botzolakis, E. J., Surdyka, K., Wang, L., & Malow, B. A. (2011). Relation of melatonin to sleep architecture in children with autism. *Journal of Autism and Developmental Disorders, 41*, 427-433. | Not enough data |
| Loddenkemper, T., Sullivan, J., McConnell, K., Coulter, D., Braga-Kenyon, P., Kothare, S., & Lockley, S. (2012). *RELATIONSHIP BETWEEN SLEEP-DEFICIENCY AND POOR DAYTIME-BEHAVIOR IN CHILDREN WITH AUTISM-SPECTRUM-DISORDER.* Paper presented at the Sleep. | Not enough data |
| MacDonald, L. L., Loring, W., Wyatt, A., Fawkes, D. B., Gray, L., & Malow, B. A. (2019). 0749 Behavioral Sleep Education in Children with Autism and Insomnia: Partnership with Community Practices. *Sleep, 42*(Supplement_1), A300-A301. | Not enough data |
| Malow, B. A., Adkins, K. W., McGrew, S. G., Surdyka, K., & Wofford, D. (2009). *Supplemental Melatonin Improves Sleep in Children with Autism Spectrum Disorders.* Paper presented at the ANNALS OF NEUROLOGY. | Not enough data |
| Malow, B. A., Goldman, S. E., Fawkes, D., Goodpaster, R. L., Adkins, K. W., & Peterson, B. T. (2014). A novel actigraphy analysis method for detecting the effects of treatment on disturbed sleep in children with autism. Sleep, 37, A371-A372. | Not enough data |
| Malow, B., Adkins, K., Clemons, T., Goldman, S., Molloy, C., Wofford, D., . . . Surdyka, K. (2011). *Effects of a standardized pamphlet on sleep latency in children with autism.* Paper presented at the Sleep. | Not enough data |
| Malow, B., Adkins, K., McGrew, S., Surdyka, K., Goldman, S., & Wofford, D. (2009). *IMPACT OF SUPPLEMENTAL MELATONIN ON SLEEP AND BEHAVIOR IN CHILDREN WITH AUTISM SPECTRUM DISORDERS.* Paper presented at the Sleep. | Not enough data |
| Malow, B., Reynolds, A., Weiss, S., Adkins, K., Artibee, K., Clemons, T., . . . Loh, A. (2012). *PARENT-BASED SLEEP EDUCATION PROGRAM FOR CHILDREN WITH AUTISM.* Paper presented at the Sleep. | Not enough data |
| McCrae, C. S., Chan, W. S., Deroche, C. B., Munoz, M., McLean, D., Davenport, M., . . . Mazurek, M. (2018). CBT FOR INSOMNIA IN CHILDREN WITH AUTISM SPECTRUM DISORDER (ASD). *Sleep, 41*, A298-A298. | Not enough data |
| McGovney, K. D., Curtis, A. F., Mazurek, M., Chan, W. S., Deroche, C. B., Munoz, M., . . . McCrae, C. S. (2020). NIGHTLY ASSOCIATIONS BETWEEN PRE-BEDTIME ACTIVITY, ACTIGRAPHIC LIGHT, AND SLEEP IN CHILDREN WITH ASD AND INSOMNIA. *Sleep, 43*, A350-A351. | Not enough data |
| Nguyen, A. D., Murphy, L. E., Kocak, M., Tylavsky, F. A., & Pagani, L. S. (2018). Prospective associations between infant sleep at 12 months and autism spectrum disorder screening scores at 24 months in a community-based birth cohort. *The Journal of Clinical Psychiatry, 79*(1), 5332. | Not enough data |
| Oriel, K. N., Kanupka, J. W., DeLong, K. S., & Noel, K. (2016). The impact of aquatic exercise on sleep behaviors in children with autism spectrum disorder: A pilot study. *Focus on Autism and Other Developmental Disabilities, 31*(4), 254-261. | Not enough data |
| Palka, T., Peura, C., Verdi, M., Kaplan, D., Beresford, C. A., Sannar, E. M., & Grados, M. (2016). 3.30 SLEEP DISTURBANCE IN HOSPITALIZED CHILDREN WITH AUTISM SPECTRUM DISORDER: RELATIONSHIP TO PARENT REPORT AND PARENTAL STRESS. *Journal of the American Academy of Child & Adolescent Psychiatry, 55*(10), S151-S152. | Not enough data |
| Pallia, R. (2012). Sleep disorder in autism spectrum disorders. *Neuropsychiatrie de l'enfance et de l'adolescence, 5*(60), S58-S59. | Not enough data |
| Papadopoulos, N., Sciberras, E., Hiscock, H., Mulraney, M., McGillivray, J., & Rinehart, N. (2015). The effectiveness of a brief behavioural sleep intervention in school aged children with ADHD and comorbid autism spectrum disorder. | Not enough data |
| Pasula, E. Y., Anderson, C., Golden, K., Fletcher, F., & Cornish, K. (2015). Sleep disturbance in malaysian and australian children with autism spectrum disorder: A cross-cultural comparison. *Sleep and Biological Rhythms, 13*, 31. | Not enough data |
| Posar, A., & Visconti, P. (2020). Sleep problems in children with autism spectrum disorder. *Pediatric Annals, 49*(6), e278-e282. | Not enough data |
| Richards, C., Trickett, J., Heald, M., Surtees, A., Clarkson, E., & Oliver, C. (2016). *Sleep disorders and painful health conditions in children with autism spectrum disorder, Smith-Magenis syndrome and Angelman syndrome.* Paper presented at the Journal of Intellectual Disability Research. | Not enough data |
| Richdale, A. (2013). Examining family demographic and environmental effects on sleep in children aged 2-5 years with and without autism spectrum disorder. *Sleep and Biological Rhythms, 11*, 8. | Not enough data |
| Richdale, A., & Roussis, S. (2018). Sleep problem severity and behaviour in children with autism aged 2-to 5-years. *Journal of Sleep Research, 27*. | Not enough data |
| Richdale, A., & Uljarevic, M. (2016). *Does intolerance of uncertainty contribute to insomnia symptoms in young people with autism?* Paper presented at the JOURNAL OF INTELLECTUAL DISABILITY RESEARCH. | Not enough data |
| Rinehart, N., Papadopoulos, N., Whelan, M., & Austin, D. (2016). *Exploring the association between family functioning and sleep problems in children with autism spectrum disorder: A pilot study.* Paper presented at the JOURNAL OF INTELLECTUAL DISABILITY RESEARCH. | Not enough data |
| Rinehart, N., Sciberras, E., Papadopoulos, N., Mulraney, M., Williams, K., Busija, L., . . . Hiscock, H. (2015). Understanding sleep problems in children with autism spectrum disorder. *Sleep and Biological Rhythms, 13*, 51. | Not enough data |
| Saletin, J., Koopman-Verhoeff, M., Han, G., Barker, D., Carskadon, M., Anders, T., & Sheinkopf, S. (2020). 1002 Endorsement Of Sleep Problems Indexes Autism Severity In Children And Adolescents: Evidence From A Large Community Sample. *Sleep, 43*, A380-A381. | Not enough data |
| Sayed, R., Bhalerao, N., Hegde, A., & Devnani, P. (2012). *PILOT STUDY: SLEEP CHARACTERISTCS IN CHILDREN WITH AUTISM SPECTRUM DISORDER.* Paper presented at the Sleep. | Not enough data |
| Schreck, K., Sims, C., Schreck, R., & Butter, E. (2016). *Possible relationships among daytime behaviour, mental health and sleep problems for children with autism spectrum disorder.* Paper presented at the JOURNAL OF INTELLECTUAL DISABILITY RESEARCH. | Not enough data |
| Serrano-Drozdowskyj, E., Fernandez, A., Moreno, C., Llorente, C., Dorado, M., & Parellada, M. (2015). *Prevalence and clinical correlates of Sleep Disorders in young people with Autism Spectrum Disorders.* Paper presented at the EUROPEAN NEUROPSYCHOPHARMACOLOGY. | Not enough data |
| Shut, A. M., Malow, B. A., & Katz, T. (2015). Item analysis of the Children's Sleep Habits Questionnaire in the autism Speaks-Autism Treatment Network registry data. Sleep, 38, A366. | Not enough data |
| Sikora, D. M., Johnson, K., Clemons, T., & Katz, T. (2012). The relationship between sleep problems and daytime behavior in children of different ages with autism spectrum disorders. *Pediatrics, 130* (Supplement_2), S83-S90. | Not enough data |
| Smidt, S. E., Ghorai, A., Gehringer, B., Dow, H., Griffiths, Z., Taylor, S., . . . Bucan, M. (2019). Sleep in Autism Spectrum Disorder Without Intellectual Disability. *Neuropsychopharmacology, 44*(SUPPL 1), 188-189. | Not enough data |
| Sun, F., Cao, M., & Jing, J. (2018). Sleep problems in children with autism spectrum disorder. *Zhonghua er ke za zhi= Chinese Journal of Pediatrics, 56*(10), 790-793. | Not enough data |
| Taira, M., Takase, M., & Sasaki, H. (1998). Sleep disorder in children with autism. *Psychiatry and clinical neurosciences, 52*(2), 182-183. | Not enough data |
| TAKASE, M., TAIRA, M., & SASAKI, H. (1998). Sleep‐wake rhythm of autistic children. *Psychiatry and clinical neurosciences, 52*(2), 181-182. | Not enough data |
| Thomas, S., Papadopoulos, N., Barnett, L., Hinkley, T., Salmon, J., May, T., . . . McGillivray, J. (2016). *Physical activity and behavioural sleep problems in young people with autism spectrum disorder.* Paper presented at the JOURNAL OF INTELLECTUAL DISABILITY RESEARCH. | Not enough data |
| Tse, A. C. Y., Lee, P. H., Zhang, J., & Lai, E. W. H. (2018). Study protocol for a randomised controlled trial examining the association between physical activity and sleep quality in children with autism spectrum disorder based on the melatonin-mediated mechanism model. *BMJ Open, 8*(4). | Not enough data |
| Tzischinsky, O., Manelis, L., Bar-Sinai, A., Fluser, H., Michaelovski, A., Zivan, O., . . . Dinstein, I. (2016). *Children with autism who have sleep problems exhibit abnormally high sensory sensitivities.* Paper presented at the Journal of Sleep Research. | Not enough data |
| Veatch, O. J., Sutcliffe, J. S., Warren, Z. E., Potter, M. H., & Malow, B. A. (2016). Multivariate analysis of insomnia symptoms in children with autism spectrum disorder reveals connection with constipation and attention deficit disorder. Sleep, 39, A5. | Not enough data |
| Villalobos, R., Jimenez, J., & Rangel Chavez, J. (2016). Sleep in children with autistic spectrum disorders and epilepsy. Sleep, 39, A334. | Not enough data |
| Wang, G., Liu, Z., Lu, N., Lewin, D., Xu, G., & Owens, J. (2014). Sleep disturbances in Chinese children with autism spectrum disorders: Characteristics and associated factors. Sleep, 37, A291. | Not enough data |
| Williams, K. C., Christofi, F. L., Clemmons, T., Rosenberg, D., & Fuchs, G. J. (2012). Mo1987 Association of Chronic Gastrointestinal Symptoms With Sleep Problems May Help Identify Distinct Subgroups of Autism Spectrum Disorders. *Gastroenterology, 5*(142), S-714. | Not enough data |
| Winkelman, T., Naples, A., Rolison, M., McNaughton, K., Day, T., Hasselmo, S., . . . Lewis, B. (2018). Children with Autism Demonstrate Atypical Resting EEG Correlates of Sleepiness. *Age (years), 9*(12), 15. | Not enough data |
| Youssef, J., Singh, K., Huntington, N., Becker, R., & Kothare, S. V. (2013). Relationship of serum ferritin levels to sleep fragmentation and periodic limb movements of sleep on polysomnography in autism spectrum disorders. *Pediatric Neurology, 49*(4), 274-278. | Not enough data |
| Adkins, K. W., Molloy, C., Weiss, S. K., Reynolds, A., Goldman, S. E., Burnette, C., . . . Malow, B. A. (2012). Effects of a standardized pamphlet on insomnia in children with autism spectrum disorders. *Pediatrics, 130*(Supplement_2), S139-S144. | Patients took medication |
| AlBacker, N., & Bashir, S. (2017). Assessment of parent report and actigraphy for sleep in children with autism spectrum disorders. Pilot study. *Neurology, Psychiatry and Brain Research, 23*, 16-19. | Patients took medication |
| Alder, M. L., Ye, F., Run, F., Bagai, K., Fawkes, D. B., Peterson, B. T., & Malow, B. A. (2020). Application of a novel actigraphy algorithm to detect movement and sleep/wake patterns in children with autism spectrum disorder. *Sleep medicine, 71*, 28-34. | Patients took medication |
| Al-Farsi, O. A., Al-Farsi, Y. M., Al-Sharbati, M. M., & Al-Adawi, S. (2019). Sleep habits and sleep disorders among children with autism spectrum disorders, intellectual disabilities and typically developing children in Oman: a case-control study. *Early Child Development and Care, 189*(14), 2370-2380. | Patients took medication |
| Andersen, I. M., Kaczmarska, J., McGrew, S. G., & Malow, B. A. (2008). Melatonin for insomnia in children with autism spectrum disorders. *Journal of child neurology, 23*(5), 482-485. | Patients took medication |
| Arazi, A., Meiri, G., Danan, D., Michaelovski, A., Flusser, H., Menashe, I., . . . Dinstein, I. (2020). Reduced sleep pressure in young children with autism. *Sleep, 43*(6), zsz309. | Patients took medication |
| Benson, S., Bender, A. M., Wickenheiser, H., Naylor, A., Clarke, M., Samuels, C. H., & Werthner, P. (2019). Differences in sleep patterns, sleepiness, and physical activity levels between young adults with autism spectrum disorder and typically developing controls. *Developmental neurorehabilitation, 22*(3), 164-173. | Patients took medication |
| Brand, S., Jossen, S., Holsboer-Trachsler, E., Pühse, U., & Gerber, M. (2015). Impact of aerobic exercise on sleep and motor skills in children with autism spectrum disorders–a pilot study. *Neuropsychiatric Disease and Treatment*, 1911-1920. | Patients took medication |
| Cebreros-Paniagua, R., Ayala-Guerrero, F., & Mateos-Salgado, E. L. (2020). Sleep disturbances in patients with Asperger syndrome related to the severity of their symptoms. *Sleep and Vigilance, 4*, 161-166. | Patients took medication |
| Cohen, S., Fulcher, B. D., Rajaratnam, S. M., Conduit, R., Sullivan, J. P., Hilaire, M. A. S., . . . McConnell, K. (2017). Behaviorally-determined sleep phenotypes are robustly associated with adaptive functioning in individuals with low functioning autism. *Scientific Reports, 7*(1), 14228. | Patients took medication |
| Cremone-Caira, A., Buirkle, J., Gilbert, R., Nayudu, N., & Faja, S. (2019). Relations between caregiver-report of sleep and executive function problems in children with autism spectrum disorder and attention-deficit/hyperactivity disorder. *Research in developmental disabilities, 94*, 103464. | Patients took medication |
| Durand, V. M. (2002). Treating sleep terrors in children with autism. *Journal of Positive Behavior Interventions, 4*(2), 66-72. | Patients took medication |
| Elkhatib Smidt, S. D., Lu, F., Rao, S. R., Asato, M., & Handen, B. L. (2020). Primary caregiver education level and sleep problems in children with autism spectrum disorder. *Journal of Sleep Research, 29*(5), e12932. | Patients took medication |
| Frazier, T. W., Krishna, J., Klingemier, E., Beukemann, M., Nawabit, R., & Ibrahim, S. (2017). A randomized, crossover trial of a novel sound-to-sleep mattress technology in children with autism and sleep difficulties. *Journal of Clinical Sleep Medicine, 13*(1), 95-104. | Patients took medication |
| Garcia, J. M., Leahy, N., Rivera, P., Brazendale, K., & Rice, D. J. (2020). The association among demographic factors, health behaviors and sleep quality in youth with Autism Spectrum Disorder. *Disability and Health Journal, 13*(3), 100885. | Patients took medication |
| Gringras, P., Green, D., Wright, B., Rush, C., Sparrowhawk, M., Pratt, K., . . . Zaiwalla, Z. (2014). Weighted blankets and sleep in autistic children—A randomized controlled trial. *Pediatrics, 134*(2), 298-306. | Patients took medication |
| Halstead, E. J., Joyce, A., Sullivan, E., Tywyn, C., Davies, K., Jones, A., & Dimitriou, D. (2021). Sleep disturbances and patterns in children with neurodevelopmental conditions. *Frontiers in pediatrics, 9*, 637770. | Patients took medication |
| Harris, J., Malow, B., & Werkhaven, J. (2018). 0787 Descriptive Epidemiology of Obstructive Sleep Apnea in Children with Autism Spectrum Disorder. *Sleep, 41*, A292. | Patients took medication |
| Healy, S., Brewer, B., Garcia, J., Daly, J., & Patterson, F. (2021). Sweat, Sit, Sleep: A Compositional Analysis of 24‐hr Movement Behaviors and Body Mass Index among Children with Autism Spectrum Disorder. *Autism Research, 14*(3), 545-550. | Patients took medication |
| Hoffman, C. D., Sweeney, D. P., Lopez-Wagner, M. C., Hodge, D., Nam, C. Y., & Botts, B. H. (2008). Children with autism: Sleep problems and mothers' stress. *Focus on Autism and Other Developmental Disabilities, 23*(3), 155-165. | Patients took medication |
| Holingue, C., Volk, H., Crocetti, D., Gottlieb, B., Spira, A. P., & Mostofsky, S. H. (2021). Links between parent-reported measures of poor sleep and executive function in childhood autism and attention deficit hyperactivity disorder. *Sleep Health, 7*(3), 375-383. | Patients took medication |
| Hollway, J. A., Aman, M. G., & Butter, E. (2013). Correlates and risk markers for sleep disturbance in participants of the autism treatment network. *Journal of Autism and Developmental Disorders, 43*, 2830-2843. | Patients took medication |
| Hollway, J. A., Mendoza-Burcham, M., Andridge, R., Aman, M. G., Handen, B., Arnold, L. E., . . . Smith, T. (2018). Atomoxetine, parent training, and their effects on sleep in youth with autism spectrum disorder and attention-deficit/hyperactivity disorder. *Journal of Child and Adolescent Psychopharmacology, 28*(2), 130-135. | Patients took medication |
| Honomichl, R. D., Goodlin-Jones, B. L., Burnham, M. M., Hansen, R. L., & Anders, T. F. (2002). Secretin and sleep in children with autism. *Child Psychiatry and Human Development, 33*, 107-123. | Patients took medication |
| Irwanto, Rehatta, N. M., Hartini, S., & Takada, S. (2016). Sleep problem of children with autistic spectrum disorder assessed by children sleep habits questionnaire-abbreviated in Indonesia and Japan. Kobe Journal of Medical Sciences, 62(2), E22-E26. | Patients took medication |
| Johnson, C. R., DeMand, A., Lecavalier, L., Smith, T., Aman, M., Foldes, E., & Scahill, L. (2016). Psychometric properties of the children's sleep habits questionnaire in children with autism spectrum disorder. *Sleep medicine, 20*, 5-11. | Patients took medication |
| Johnson, C. R., Smith, T., DeMand, A., Lecavalier, L., Evans, V., Gurka, M., . . . Scahill, L. (2018). Exploring sleep quality of young children with autism spectrum disorder and disruptive behaviors. *Sleep medicine, 44*, 61-66. | Patients took medication |
| Johnson, C. R., Turner, K. S., Foldes, E. L., Malow, B. A., & Wiggs, L. (2012). Comparison of sleep questionnaires in the assessment of sleep disturbances in children with autism spectrum disorders. *Sleep medicine, 13*(7), 795-801. | Patients took medication |
| Katz, T., Shui, A. M., Johnson, C. R., Richdale, A. L., Reynolds, A. M., Scahill, L., & Malow, B. A. (2018). Modification of the children’s sleep habits questionnaire for children with autism spectrum disorder. *Journal of Autism and Developmental Disorders, 48*, 2629-2641. | Patients took medication |
| Kirkpatrick, B., Gilroy, S. P., & Leader, G. (2019). Qualitative study on parents’ perspectives of the familial impact of living with a child with autism spectrum disorder who experiences insomnia. *Sleep medicine, 62*, 59-68. | Patients took medication |
| Koo, H. W., Ismail, J., Yang, W. W., & Syed Zakaria, S. Z. (2020). Sleep Disturbances in Children With Autism Spectrum Disorder at a Malaysian Tertiary Hospital. *Frontiers in pediatrics, 8*. | Patients took medication |
| Lindor, E., Sivaratnam, C., May, T., Stefanac, N., Howells, K., & Rinehart, N. (2019). Problem behavior in autism spectrum disorder: considering core symptom severity and accompanying sleep disturbance. *Frontiers in Psychiatry, 10*, 487. | Patients took medication |
| Liu, X., Hubbard, J. A., Fabes, R. A., & Adam, J. B. (2006). Sleep disturbances and correlates of children with autism spectrum disorders. *Child Psychiatry and Human Development, 37*, 179-191. | Patients took medication |
| MacQuarrie, J. (2009). Parental attributions regarding sleep problems of children with an Autism Spectrum Disorder or Down Syndrome. *Parental attributions regarding sleep problems of children with an Autism Spectrum Disorder or Down Syndrome AND CLINICAL RESEARCH PORTFOLIO*, 57. | Patients took medication |
| Malow, B. A., Katz, T., Reynolds, A. M., Shui, A., Carno, M., Connolly, H. V., . . . Bennett, A. E. (2016). Sleep difficulties and medications in children with autism spectrum disorders: a registry study. *Pediatrics, 137*(Supplement_2), S98-S104. | Patients took medication |
| Mannion, A., & Leader, G. (2016). An investigation of comorbid psychological disorders, sleep problems, gastrointestinal symptoms and epilepsy in children and adolescents with autism spectrum disorder: A two year follow-up. *Research in autism spectrum disorders, 22*, 20-33. | Patients took medication |
| Mannion, A., Leader, G., & Healy, O. (2013). An investigation of comorbid psychological disorders, sleep problems, gastrointestinal symptoms and epilepsy in children and adolescents with autism spectrum disorder. *Research in autism spectrum disorders, 7*(1), 35-42. | Patients took medication |
| Maras, A., Schroder, C. M., Malow, B. A., Findling, R. L., Breddy, J., Nir, T., . . . Gringras, P. (2018). Long-term efficacy and safety of pediatric prolonged-release melatonin for insomnia in children with autism spectrum disorder. *Journal of Child and Adolescent Psychopharmacology, 28*(10), 699-710. | Patients took medication |
| Mazurek, M. O., & Petroski, G. F. (2015). Sleep problems in children with autism spectrum disorder: examining the contributions of sensory over-responsivity and anxiety. *Sleep medicine, 16*(2), 270-279. | Patients took medication |
| Mazurek, M. O., Dovgan, K., Neumeyer, A. M., & Malow, B. A. (2019). Course and predictors of sleep and co-occurring problems in children with autism spectrum disorder. *Journal of Autism and Developmental Disorders, 49*, 2101-2115. | Patients took medication |
| Mazurek, M. O., Engelhardt, C. R., Hilgard, J., & Sohl, K. (2016). Bedtime electronic media use and sleep in children with autism spectrum disorder. *Journal of Developmental & Behavioral Pediatrics, 37*(7), 525-531. | Patients took medication |
| McLay, L., France, K., Blampied, N., & Hunter, J. (2019). Using functional behavioral assessment to treat sleep problems in two children with autism and vocal stereotypy. *International Journal of Developmental Disabilities, 65*(3), 175-184. | Patients took medication |
| Mehrazad‐Saber, Z., Kheirouri, S., & Noorazar, S. G. (2018). Effects of l‐carnosine supplementation on sleep disorders and disease severity in autistic children: a randomized, controlled clinical trial. *Basic & clinical pharmacology & toxicology, 123*(1), 72-77. | Patients took medication |
| Merikanto, I., Kuula, L., Makkonen, T., Salmela, L., Räikkönen, K., & Pesonen, A.-K. (2019). Autistic traits are associated with decreased activity of fast sleep spindles during adolescence. *Journal of Clinical Sleep Medicine, 15*(3), 401-407. | Patients took medication |
| Neumeyer, A. M., Anixt, J., Chan, J., Perrin, J. M., Murray, D., Coury, D. L., . . . Parker, R. A. (2019). Identifying associations among co-occurring medical conditions in children with autism spectrum disorders. *Academic Pediatrics, 19*(3), 300-306. | Patients took medication |
| Øyane, N. M., & Bjorvatn, B. (2005). Sleep disturbances in adolescents and young adults with autism and Asperger syndrome. *Autism, 9*(1), 83-94. | Patients took medication |
| Papadopoulos, N., Sciberras, E., Hiscock, H., Mulraney, M., McGillivray, J., & Rinehart, N. (2019). The efficacy of a brief behavioral sleep intervention in school-aged children with ADHD and comorbid autism spectrum disorder. *Journal of attention disorders, 23*(4), 341-350. | Patients took medication |
| Phung, J. N., Abdullah, M. M., & Goldberg, W. A. (2019). Poor sleep quality among adolescents with ASD is associated with depressive symptoms, problem behaviors, and conflicted family relationships. *Focus on Autism and Other Developmental Disabilities, 34*(3), 173-182. | Patients took medication |
| Restrepo, B., Angkustsiri, K., Taylor, S. L., Rogers, S. J., Cabral, J., Heath, B., . . . Amaral, D. G. (2020). Developmental–behavioral profiles in children with autism spectrum disorder and co‐occurring gastrointestinal symptoms. *Autism Research, 13*(10), 1778-1789. | Patients took medication |
| Richdale, A. L., & Schreck, K. A. (2019). Examining sleep hygiene factors and sleep in young children with and without autism spectrum disorder. *Research in autism spectrum disorders, 57*, 154-162. | Patients took medication |
| Roussis, S., Richdale, A. L., Katz, T., Malow, B. A., Barbaro, J., & Sadka, N. (2021). Behaviour, cognition, and autism symptoms and their relationship with sleep problem severity in young children with autism spectrum disorder. *Research in autism spectrum disorders, 83*, 101743. | Patients took medication |
| Sanberg, S. A., Kuhn, B. R., & Kennedy, A. E. (2018). Outcomes of a behavioral intervention for sleep disturbances in children with autism spectrum disorder. *Journal of Autism and Developmental Disorders, 48*, 4250-4277. | Patients took medication |
| Schreck, K. A., Mulick, J. A., & Smith, A. F. (2004). Sleep problems as possible predictors of intensified symptoms of autism. *Research in developmental disabilities, 25*(1), 57-66. | Patients took medication |
| Shui, A. M., Lampinen, L. A., Zheng, S., & Katz, T. (2021). Characteristics associated with parental estimates of sleep duration in children with autism spectrum disorders. *Research in autism spectrum disorders, 80*, 101698. | Patients took medication |
| Souders, M. C., Mason, T. B., Valladares, O., Bucan, M., Levy, S. E., Mandell, D. S., . . . Pinto-Martin, J. (2009). Sleep behaviors and sleep quality in children with autism spectrum disorders. *Sleep, 32*(12), 1566-1578. | Patients took medication |
| Surtees, A. D., Richards, C., Clarkson, E. L., Heald, M., Trickett, J., Denyer, H., . . . Oliver, C. (2019). Sleep problems in autism spectrum disorders: A comparison to sleep in typically developing children using actigraphy, diaries and questionnaires. *Research in autism spectrum disorders, 67*, 101439. | Patients took medication |
| Taylor, B. J., & Siegel, M. (2019). 0783 Characterization Of Sleep Disturbance Profiles In Hospitalized Children With Autism Spectrum Disorder. *Sleep, 42*(Supplement_1), A314-A315. | Patients took medication |
| Taylor, B., Palka, T., Grados, M., Peura, C., Verdi, M., & Siegel, M. (2018). 0841 Discrepancies between Parent-Reported and Observed Sleep Disturbance in Hospitalized Children with Autism Spectrum Disorder (ASD). *Sleep, 41*(suppl_1), A312-A312. | Patients took medication |
| Thenhausen, N., Kuss, M., Wiater, A., & Schlarb, A. A. (2017). Sleep problems in adolescents with Asperger syndrome or high-functioning autism. *Somnologie, 21*(3), 218-228. | Patients took medication |
| Thirumalai, S. S., Shubin, R. A., & Robinson, R. (2002). Rapid eye movement sleep behavior disorder in children with autism. *Journal of child neurology, 17*(3), 173-178. | Patients took medication |
| Tomkies, A., Johnson, R. F., & Mitchell, R. B. (2018). Obstructive sleep apnea in children with autism spectrum disorder. *Otolaryngology - Head and Neck Surgery (United States), 159*(1), P308. | Patients took medication |
| Tsai, T. H., Chen, Y. L., & Gau, S. S. (2021). Relationships between autistic traits, insufficient sleep, and real-world executive functions in children: a mediation analysis of a national epidemiological survey. *Psychol Med, 51*(4), 579-586. | Patients took medication |
| Tse, C. Y. A., Lee, H. P., Chan, K. S. K., Edgar, V. B., Wilkinson-Smith, A., & Lai, W. H. E. (2019). Examining the impact of physical activity on sleep quality and executive functions in children with autism spectrum disorder: A randomized controlled trial. *Autism, 23*(7), 1699-1710. | Patients took medication |
| Tudor, M. E., Walsh, C. E., Mulder, E. C., & Lerner, M. D. (2015). Pain as a predictor of sleep problems in youth with autism spectrum disorders. *Autism, 19*(3), 292-300. | Patients took medication |
| Veatch, O. J., Reynolds, A., Katz, T., Weiss, S. K., Loh, A., Wang, L., & Malow, B. A. (2016). Sleep in children with autism spectrum disorders: How are measures of parent report and actigraphy related and affected by sleep education? *Behavioral sleep medicine, 14*(6), 665-676. | Patients took medication |
| Wachob, D., & Lorenzi, D. G. (2015). Brief report: Influence of physical activity on sleep quality in children with autism. *Journal of Autism and Developmental Disorders, 45*, 2641-2646. | Patients took medication |
| Wiggs, L., & Stores, G. (2004). Sleep patterns and sleep disorders in children with autistic spectrum disorders: insights using parent report and actigraphy. *Developmental medicine and child neurology, 46*(6), 372-380. | Patients took medication |
| Williams, T. I. (2006). Evaluating effects of aromatherapy massage on sleep in children with autism: a pilot study. *Evidence-Based Complementary and Alternative Medicine, 3*(3), 373-377. | Patients took medication |
| Yavuz-Kodat, E., Reynaud, E., Geoffray, M.-M., Limousin, N., Franco, P., Bonnet-Brilhault, F., . . . Schroder, C. M. (2020). Disturbances of continuous sleep and circadian rhythms account for behavioral difficulties in children with autism spectrum disorder. *Journal of clinical medicine, 9*(6), 1978. | Patients took medication |
| Yavuz-Kodat, E., Reynaud, E., Geoffray, M.-M., Limousin, N., Franco, P., Bourgin, P., & Schroder, C. M. (2019). Validity of actigraphy compared to polysomnography for sleep assessment in children with autism spectrum disorder. *Frontiers in Psychiatry, 10*, 551. | Patients took medication |
| Yu, X., Lam, H., Au, C., Chan, S., Chan, D., & Li, A. (2015). Extended parent-based behavioural education improves sleep in children with autism spectrum disorder. *HK J Paediatr, 20*(4), 219-225. | Patients took medication |
| Zaidman-Zait, A., Zwaigenbaum, L., Duku, E., Bennett, T., Szatmari, P., Mirenda, P., . . . Waddell, C. (2020). Factor analysis of the children’s sleep habits questionnaire among preschool children with autism spectrum disorder. *Research in developmental disabilities, 97*, 103548. | Patients took medication |
| Zwaigenbaum, L., Zaidman-Zait, A., Duku, E., Bennett, T., Mirenda, P., Smith, I., . . . Elsabbagh, M. (2020). 55 Profiles of Sleep Problems among Young Children with Autism Spectrum Disorders. *Paediatrics & Child Health, 25*, e23. | Patients took medication |
| Abel, E. A., Schwichtenberg, A., Brodhead, M. T., & Christ, S. L. (2018). Sleep and challenging behaviors in the context of intensive behavioral intervention for children with autism. *Journal of Autism and Developmental Disorders, 48*, 3871-3884. | Out of scope |
| Accardo, J., Freedman, B., Kalb, L., Vaurio, R., Goldman, S., & Malow, B. (2011). *ADDRESSING SLEEP PROBLEMS IN CHILDREN WITH AUTISM SPECTRUM DISORDERS AT A MULTIDISCIPLINARY AUTISM CENTER.* Paper presented at the Sleep. | Out of scope |
| Adams, H. L., Matson, J. L., & Jang, J. (2014). The relationship between sleep problems and challenging behavior among children and adolescents with autism spectrum disorder. *Research in autism spectrum disorders, 8*(9), 1024-1030. | Out of scope |
| Adams, H. L., Matson, J. L., Cervantes, P. E., & Goldin, R. L. (2014). The relationship between autism symptom severity and sleep problems: Should bidirectionality be considered. *Research in autism spectrum disorders, 8*(3), 193-199. | Out of scope |
| Baird, G., Robinson, R. O., Boyd, S., & Charman, T. (2006). Sleep electroencephalograms in young children with autism with and without regression. *Developmental medicine and child neurology, 48*(7), 604-608. | Out of scope |
| Berrillo-Batista, S., Morales-Chacon, L. M., Baez-Martin, M. M., Gomez-Fernandez, L., Vera-Cuesta, H., Maragoto-Rizo, C., . . . Perez-Mayo, L. (2018). Functional connectivity derived from an electroencephalogram during non-REM sleep in autism spectrum disorders. *Revista de Neurologia, 67*(2), 41-49. | Out of scope |
| Carlson, C., Suliman, A., Alivar, A., Prakash, P., Thompson, D., Natarajan, B., & Warren, S. (2018). *A pilot study of an unobtrusive bed-based sleep quality monitor for severely disabled autistic children.* Paper presented at the 2018 40th Annual International Conference of the IEEE Engineering in Medicine and Biology Society (EMBC). | Out of scope |
| Carlson, C., Suliman, A., Prakash, P., Thompson, D., Wang, S., Natarajan, B., & Warren, S. (2016). *Bed-based instrumentation for unobtrusive sleep quality assessment in severely disabled autistic children.* Paper presented at the 2016 38th Annual International Conference of the IEEE Engineering in Medicine and Biology Society (EMBC). | Out of scope |
| Carnett, A., McLay, L., Hansen, S., France, K., & Blampied, N. (2021). Sleep problems in children and adolescents with autism: Type, severity and impact. *Journal of Developmental and Physical Disabilities*, 1-15. | Out of scope |
| Cavalieri, A. (2016). Sleep issues in children with autism spectrum disorder. *Pediatric Nursing, 42*(4), 169. | Out of scope |
| Cohen, S., Fulcher, B. D., Rajaratnam, S. M., Conduit, R., Sullivan, J. P., St Hilaire, M. A., . . . McConnell, K. (2018). Sleep patterns predictive of daytime challenging behavior in individuals with low‐functioning autism. *Autism Research, 11*(2), 391-403. | Out of scope |
| D'Agati, E., Abate, R., Gialloreti, L., Napolitano, C., Postorino, V., Curatolo, P., & Mazzone, L. (2020). Sleep problems in attention-deficit/hyperactivity disorder and autism spectrum disorder: Sex differences and parental stress. *Psychiatry Research, 291*, 113099. | Out of scope |
| DelRosso, L., & Ferri, R. (2019). The prevalence of restless sleep disorder in a single sleep center. *Sleep medicine, 64*, S89-S90. | Out of scope |
| Devnani, P. A., & Hegde, A. U. (2015). Autism and sleep disorders. *Journal of pediatric neurosciences, 10*(4), 304. | Out of scope |
| Dosman, C. F., Brian, J. A., Drmic, I. E., Senthilselvan, A., Harford, M. M., Smith, R. W., . . . Roberts, S. W. (2007). Children with autism: effect of iron supplementation on sleep and ferritin. *Pediatric Neurology, 36*(3), 152-158. | Out of scope |
| Gail Williams, P., Sears, L. L., & Allard, A. (2004). Sleep problems in children with autism. *Journal of Sleep Research, 13*(3), 265-268. | Out of scope |
| Gee, B. M., Lloyd, K., Sutton, J., & McOmber, T. (2020). Weighted blankets and sleep quality in children with autism spectrum disorders: a single-subject design. *Children, 8*(1), 10. | Out of scope |
| Gee, B. M., Peterson, T. G., Buck, A., & Lloyd, K. (2016). Improving sleep quality using weighted blankets among young children with an autism spectrum disorder. *International Journal of Therapy and Rehabilitation, 23*(4), 173-181. | Out of scope |
| Hayashi, E. (2000). Effect of melatonin on sleep–wake rhythm: The sleep diary of an autistic male. *Psychiatry and clinical neurosciences, 54*(3), 383-384. | Out of scope |
| Hayashi, E. (2001). Seasonal changes in sleep and behavioral problems in a pubescent case with autism. *Psychiatry and clinical neurosciences, 55*(3), 223-224. | Out of scope |
| Hochard, K. D., Pendrous, R., Mari, T., & Flynn, S. (2020). Examining the relationship between autism traits and sleep duration as predictors of suicidality. *Journal of Autism and Developmental Disorders, 50*, 3575-3584. | Out of scope |
| Horiuchi, F., Kawabe, K., Oka, Y., Nakachi, K., Hosokawa, R., & Ueno, S.-I. (2020). The association between autistic traits and sleep habits/problems in toddlers. *Developmental neuropsychology, 45*(7-8), 485-495. | Out of scope |
| Huda, N., Saidah, Q. i., Wardani, R. N., & Muflikhah, M. (2020). Effectiveness of Chamomile Tea toward Sleep Quality Amount Autistic Children. *Journal of Global Pharma Technology, 12*(1), 426-432. | Out of scope |
| Hundley, R. J., Shui, A., & Malow, B. A. (2016). Relationship between subtypes of restricted and repetitive behaviors and sleep disturbance in autism spectrum disorder. *Journal of Autism and Developmental Disorders, 46*, 3448-3457. | Out of scope |
| Hunter, J. E., McLay, L. K., France, K. G., & Blampied, N. M. (2021). Sleep and stereotypy in children with autism: Effectiveness of function-based behavioral treatment. *Sleep medicine, 80*, 301-304. | Out of scope |
| Johansson, A. E., Feeley, C. A., Dorman, J. S., & Chasens, E. R. (2018). Characteristics of sleep in children with autism spectrum disorders from the Simons Simplex Collection. *Research in autism spectrum disorders, 53*, 18-30. | Out of scope |
| Johnson, K. P., & Malow, B. A. (2008). Assessment and pharmacologic treatment of sleep disturbance in autism. *Child and adolescent psychiatric clinics of North America, 17*(4), 773-785. | Out of scope |
| Johnson, K. P., Giannotti, F., & Cortesi, F. (2009). Sleep patterns in autism spectrum disorders. *Child and Adolescent Psychiatric Clinics, 18*(4), 917-928. | Out of scope |
| Katz, T., Malow, B. A., Goldman, S. E., Shui, A., Sohl, K. A., & Coury, D. (2013). Sensitivity and specificity of parent report measures of child sleep in children with autism spectrum disorders. Sleep, 36, A376-A377. | Out of scope |
| Kawabe, K., Horiuchi, F., Oka, Y., & Ueno, S.-i. (2014). The melatonin receptor agonist ramelteon effectively treats insomnia and behavioral symptoms in autistic disorder. *Case reports in psychiatry, 2014*. | Out of scope |
| Knight, R. M., & Johnson, C. M. (2014). Using a behavioral treatment package for sleep problems in children with autism spectrum disorders. *Child & Family Behavior Therapy, 36*(3), 204-221. | Out of scope |
| Knüppel, A., Telléus, G. K., Jakobsen, H., & Lauritsen, M. B. (2018). Quality of life in adolescents and adults with autism spectrum disorder: Results from a nationwide Danish survey using self-reports and parental proxy-reports. *Research in developmental disabilities, 83*, 247-259. | Out of scope |
| Kodak, T., & Piazza, C. C. (2008). Assessment and behavioral treatment of feeding and sleeping disorders in children with autism spectrum disorders. *Child and adolescent psychiatric clinics of North America, 17*(4), 887-905. | Out of scope |
| Kozlowski, A. M., Matson, J. L., Belva, B., & Rieske, R. (2012). Feeding and sleep difficulties in toddlers with autism spectrum disorders. *Research in autism spectrum disorders, 6*(1), 385-390. | Out of scope |
| Kuhlthau, K. A., McDonnell, E., Coury, D. L., Payakachat, N., & Macklin, E. (2018). Associations of quality of life with health-related characteristics among children with autism. *Autism, 22*(7), 804-813. | Out of scope |
| Kurz, E. M., Conzelmann, A., Barth, G. M., Hepp, L., Schenk, D., Renner, T. J., . . . Zinke, K. (2019). Signs of enhanced formation of gist memory in children with autism spectrum disorder–a study of memory functions of sleep. *Journal of Child Psychology and Psychiatry, 60*(8), 907-916. | Out of scope |
| Lesca, G., Rudolf, G., Labalme, A., Hirsch, E., Arzimanoglou, A., Genton, P., . . . Boulay, C. (2012). Epileptic encephalopathies of the Landau‐Kleffner and continuous spike and waves during slow‐wave sleep types: Genomic dissection makes the link with autism. *Epilepsia, 53*(9), 1526-1538. | Out of scope |
| Loring, W. A., L Johnston, R., Shui, A. M., & Malow, B. A. (2018). Impact of a brief behavioral intervention for insomnia on daytime behaviors in adolescents with autism spectrum disorders. *Journal of Contemporary Psychotherapy, 48*, 165-177. | Out of scope |
| Luiselli, J. K., Harper, J. M., Shlesinger, A., Murphy, K. J., & Luke, K. (2020). Faded bedtime intervention for delayed sleep onset in an adolescent with autism spectrum disorder. *Clinical Case Studies, 19*(3), 180-188. | Out of scope |
| Malow, B. A., Findling, R. L., Schroder, C. M., Maras, A., Breddy, J., Nir, T., . . . Gringras, P. (2021). Sleep, growth, and puberty after 2 years of prolonged-release melatonin in children with autism spectrum disorder. *Journal of the American Academy of Child & Adolescent Psychiatry, 60*(2), 252-261. e253. | Out of scope |
| Marvin, A. R., Coury, D. L., Malow, B. A., Law, J. K., & Bennett, A. E. (2021). Brief report: Measures of effectiveness for single-question sleep problem screeners in children with autism spectrum disorder. *Research in autism spectrum disorders, 80*, 101699. | Out of scope |
| Mayes, S. D., & Calhoun, S. L. (2009). Variables related to sleep problems in children with autism. *Research in autism spectrum disorders, 3*(4), 931-941. | Out of scope |
| Mazurek, M. O., & Sohl, K. (2016). Sleep and behavioral problems in children with autism spectrum disorder. *Journal of Autism and Developmental Disorders, 46*, 1906-1915. | Out of scope |
| McCue, L. M., Flick, L. H., Twyman, K. A., & Xian, H. (2017). Gastrointestinal dysfunctions as a risk factor for sleep disorders in children with idiopathic autism spectrum disorder: A retrospective cohort study. *Autism, 21*(8), 1010-1020. | Out of scope |
| McLay, L., France, K., Blampied, N., Danna, K., & Hunter, J. (2017). Using functional behavioral assessment to develop a multicomponent treatment for sleep problems in a 3-year-old boy with autism. *Clinical Case Studies, 16*(3), 254-270. | Out of scope |
| McLay, L., France, K., Blampied, N., van Deurs, J., Hunter, J., Knight, J., . . . Gibbs, R. (2021). Function-based behavioral interventions for sleep problems in children and adolescents with autism: Summary of 41 clinical cases. *Journal of Autism and Developmental Disorders, 51*, 418-432. | Out of scope |
| McLay, L., Hansen, S. G., Carnett, A., France, K. G., & Blampied, N. M. (2020). Attributions, causal beliefs, and help-seeking behavior of parents of children with autism spectrum disorder and sleep problems. *Autism, 24*(7), 1829-1840. | Out of scope |
| Melke, J., Goubran Botros, H., Chaste, P., Betancur, C., Nygren, G., Anckarsäter, H., . . . Delorme, R. (2008). Abnormal melatonin synthesis in autism spectrum disorders. *Molecular psychiatry, 13*(1), 90-98. | Out of scope |
| Nadeau, J. M., Arnold, E. B., Keene, A. C., Collier, A. B., Lewin, A. B., Murphy, T. K., & Storch, E. A. (2015). Frequency and clinical correlates of sleep-related problems among anxious youth with autism spectrum disorders. *Child Psychiatry & Human Development, 46*, 558-566. | Out of scope |
| Ness, S. L., Bangerter, A., Manyakov, N. V., Lewin, D., Boice, M., Skalkin, A., . . . Goodwin, M. S. (2019). An observational study with the Janssen Autism Knowledge Engine (JAKE®) in individuals with autism spectrum disorder. *Frontiers in neuroscience, 13*, 111. | Out of scope |
| Pałka-Szafraniec, K., Gmitrowicz, A., & Makowska, I. (2018). Sleep disorders in autism. *Journal of Psychiatry and Clinical Psychology, 18*(4), 413. | Out of scope |
| Pavlopoulou, G. (2020). A good night’s sleep: learning about sleep from autistic adolescents’ personal accounts. *Frontiers in Psychology, 11*, 583868. | Out of scope |
| Potter, C. A. (2017). Fathers experiences of sleeping problems in children with autism. *Advances in Autism*. | Out of scope |
| Prakash, P., Kuehl, P., McWilliams, B., Rubenthaler, S., Schnell, E., Singleton, G., & Warren, S. (2014). *Sensors and instrumentation for unobtrusive sleep quality assessment in autistic children.* Paper presented at the 2014 36th Annual International Conference of the IEEE Engineering in Medicine and Biology Society. | Out of scope |
| Reynolds, A. M., & Malow, B. A. (2011). Sleep and autism spectrum disorders. *Pediatric Clinics, 58*(3), 685-698. | Out of scope |
| Reynolds, K. C., Patriquin, M., Alfano, C. A., Loveland, K. A., & Pearson, D. A. (2017). Parent-reported problematic sleep behaviors in children with comorbid autism spectrum disorder and attention-deficit/hyperactivity disorder. *Research in autism spectrum disorders, 39*, 20-32. | Out of scope |
| Rzepecka, H., McKenzie, K., McClure, I., & Murphy, S. (2011). Sleep, anxiety and challenging behaviour in children with intellectual disability and/or autism spectrum disorder. *Research in developmental disabilities, 32*(6), 2758-2766. | Out of scope |
| Salmela, L., Kuula, L., Merikanto, I., Räikkönen, K., & Pesonen, A.-K. (2019). Autistic traits and sleep in typically developing adolescents. *Sleep medicine, 54*, 164-171. | Out of scope |
| Sannar, E. M., Palka, T., Beresford, C., Peura, C., Kaplan, D., Verdi, M., . . . Grados, M. (2018). Sleep problems and their relationship to maladaptive behavior severity in psychiatrically hospitalized children with autism spectrum disorder (ASD). *Journal of Autism and Developmental Disorders, 48*, 3720-3726. | Out of scope |
| Schreck, K. A. (2001). Behavioral treatments for sleep problems in autism: Empirically supported or just universally accepted? *Behavioral Interventions: Theory & Practice in Residential & Community‐Based Clinical Programs, 16*(4), 265-278. | Out of scope |
| Schreck, K. A., & Richdale, A. L. (2020). Sleep problems, behavior, and psychopathology in autism: inter-relationships across the lifespan. *Current Opinion in Psychology, 34*, 105-111. | Out of scope |
| Schroeder, C. (2012). *Sleep and circadian rhythm disturbances in autism.* Paper presented at the Journal of Sleep Research. | Out of scope |
| Shlesinger, A., Duhanyan, K., Bird, F., Harper, J. M., & Luiselli, J. K. (2020). Description, implementation integrity, and social validity of a computer-assisted sleep monitoring system among residential care providers of students with autism spectrum disorder. *Journal of Developmental and Physical Disabilities, 32*(2), 365-374. | Out of scope |
| Shui, A. M., Katz, T., Malow, B. A., & Mazurek, M. O. (2018). Predicting sleep problems in children with autism spectrum disorders. *Research in developmental disabilities, 83*, 270-279. | Out of scope |
| Sivertsen, B. (2010). Sleep problems in children with autism spectrum disorder. A longitudinal population-based study. *Journal of Sleep Research, 19*, 263. | Out of scope |
| Sivertsen, B., Posserud, M.-B., Gillberg, C., Lundervold, A. J., & Hysing, M. (2012). Sleep problems in children with autism spectrum problems: a longitudinal population-based study. *Autism, 16*(2), 139-150. | Out of scope |
| Souders, M., Bennett, A., & Herrington, J. (2011). *SLEEP DISORDERS IN A COHORT OF CHILDREN WITH AUTISM SPECTRUM DISORDER.* Paper presented at the Sleep. | Out of scope |
| Taylor, B. J., Reynolds, C. F., & Siegel, M. (2021). Insomnia subtypes and clinical impairment in hospitalized children with autism spectrum disorder. *Autism, 25*(3), 656-666. | Out of scope |
| Taylor, M. A., Schreck, K. A., & Mulick, J. A. (2012). Sleep disruption as a correlate to cognitive and adaptive behavior problems in autism spectrum disorders. *Research in developmental disabilities, 33*(5), 1408-1417. | Out of scope |
| Tilford, J. M., Payakachat, N., Kuhlthau, K. A., Pyne, J. M., Kovacs, E., Bellando, J., . . . Frye, R. E. (2015). Treatment for sleep problems in children with autism and caregiver spillover effects. *Journal of Autism and Developmental Disorders, 45*, 3613-3623. | Out of scope |
| TOPER, Ö. (2018). Sleep Problems and Behavioral Interventions for Children With Autism Spectrum Disorders and Intellectual Disabilities. *ANKARA UNIVERSITESI EGITIM BILIMLERI FAKULTESI OZEL EGITIM DERGISI-ANKARA UNIVERSITY FACULTY OF EDUCATIONAL SCIENCES JOURNAL OF SPECIAL EDUCATION, 19*(4). | Out of scope |
| Tudor, M. E., Hoffman, C. D., & Sweeney, D. P. (2012). Children with autism: sleep problems and symptom severity. *Focus on Autism and Other Developmental Disabilities, 27*(4), 254-262. | Out of scope |
| Uren, J., Richdale, A. L., Cotton, S. M., & Whitehouse, A. J. (2019). Sleep problems and anxiety from 2 to 8 years and the influence of autistic traits: A longitudinal study. *European child & adolescent psychiatry, 28*(8), 1117-1127. | Out of scope |
| Veatch, O. J., Goldman, S. E., Adkins, K. W., & Malow, B. A. (2015). Melatonin in children with autism spectrum disorders: How does the evidence fit together. *J Nat Sci, 1*(7), e125. | Out of scope |
| Veatch, O. J., Reynolds, A., Katz, T., Weiss, S. K., Loh, A., Wang, L., & Malow, B. A. (2015). Sleep in children with autism spectrum disorders: How are measures of parent report and actigraphy related? Sleep, 38, A277-A278. | Out of scope |
| Verhoeff, M. E., Blanken, L. M., Kocevska, D., Mileva-Seitz, V. R., Jaddoe, V. W., White, T., . . . Tiemeier, H. (2018). The bidirectional association between sleep problems and autism spectrum disorder: a population-based cohort study. *Molecular Autism, 9*, 1-9. | Out of scope |
| Vite, T. K. G., Guerrero, F. A., Salgado, E. L. M., & Paniagua, R. C. (2018). Characterization of the Mu rhythm during the sleep of children with autism spectrum disorder level 1. *Salud mental, 41*(3), 109-116. | Out of scope |
| Waddington, H., McLay, L., Woods, L., & Whitehouse, A. J. (2020). Child and family characteristics associated with sleep disturbance in children with autism spectrum disorder. *Journal of Autism and Developmental Disorders, 50*, 4121-4132. | Out of scope |
| Weiskop, S., Richdale, A., & Matthews, J. (2005). Behavioural treatment to reduce sleep problems in children with autism or fragile X syndrome. *Developmental medicine and child neurology, 47*(2), 94-104. | Out of scope |
| Williams, G., Sears, L., & Allard, A. (2006). Parent perceptions of efficacy for strategies used to facilitate sleep in children with autism. *Journal of Developmental and Physical Disabilities, 18*(1), 25-33. | Out of scope |
| Wirojanan, J., Jacquemont, S., Diaz, R., Bacalman, S., Anders, T. F., Hagerman, R. J., & Goodlin-Jones, B. L. (2009). The efficacy of melatonin for sleep problems in children with autism, fragile X syndrome, or autism and fragile X syndrome. *Journal of Clinical Sleep Medicine, 5*(2), 145-150. | Out of scope |
| Won, D. C., Feldman, H. M., & Huffman, L. C. (2019). Sleep Problem Detection and Documentation in Children With Autism Spectrum Disorder and Attention-Deficit/Hyperactivity Disorder by Developmental-Behavioral Pediatricians: A DBPNet Study. *Journal of developmental and behavioral pediatrics : JDBP, 40*(1), 20-31. | Out of scope |
| Youssef, J., Huntington, N., Gregas, M., Loddenkemper, T., Becker, R., & Kothare, S. (2011). *Periodic limb movements of sleep, serum-ferritin levels, and sleep fragmentation on polysomnogram in Autism Spectrum Disorder.* Paper presented at the ANNALS OF NEUROLOGY. | Out of scope |
| Abel, E., Kim, S. Y., Kellerman, A. M., & Brodhead, M. T. (2017). Recommendations for identifying sleep problems and treatment resources for children with autism spectrum disorder. *Behavior Analysis in Practice, 10*, 261-269. | Review article |
| Cortesi, F., Giannotti, F., Ivanenko, A., & Johnson, K. (2010). Sleep in children with autistic spectrum disorder. *Sleep medicine, 11*(7), 659-664. | Review article |
| Johnson, C. R. (1996). Sleep problems in children with mental retardation and autism. *Child and adolescent psychiatric clinics of North America, 5*(3), 673-684. | Review article |
| Kelmanson, I. (2015). Sleep disturbances in children with autistic spectrum disorders. *Zhurnal Nevrologii i Psikhiatrii Imeni SS Korsakova, 115*(4), 102-107. | Review article |
| Kotagal, S., & Broomall, E. (2012). Sleep in children with autism spectrum disorder. *Pediatric Neurology, 47*(4), 242-251. | Review article |
| Lázár, A. S., & Bódizs, R. (2008). The structure and patterns of sleep in autism spectrum disorders. *Psychiatria Hungarica: A Magyar Pszichiatriai Tarsasag Tudomanyos Folyoirata, 23*(2), 109-128. | Review article |
| Maxwell-Horn, A., & Malow, B. A. (2017). Sleep in Autism. *Seminars in Neurology, 37*(4), 413-418. | Review article |
| McLay, L.-L. K., & France, K. (2016). Empirical research evaluating non-traditional approaches to managing sleep problems in children with autism. *Developmental neurorehabilitation, 19*(2), 123-134. | Review article |
| Miano, S., & Ferri, R. (2010). Epidemiology and management of insomnia in children with autistic spectrum disorders. *Pediatric Drugs, 12*, 75-84. | Review article |
| Moore, M., Evans, V., Hanvey, G., & Johnson, C. (2017). Assessment of sleep in children with autism spectrum disorder. *Children, 4*(8), 72. | Review article |
| Schroder, C. M., Chabaux-Delarai, C., Gras-Vincendon, A., Florence, E., Danion-Grilliat, A., Bursztejn, C., & Bourgin, P. (2010). Sleep disturbances in autism spectrum disorders: Correlation with autistic core symptomatology. *Journal of Sleep Research, 19*, 203. | Review article |
| Veatch, O. J., Maxwell-Horn, A. C., & Malow, B. A. (2015). Sleep in autism spectrum disorders. *Current Sleep Medicine Reports, 1*, 131-140. | Review article |

# **eAppendix 8. Unification of sleep parameters assessed by subjective measurements**

## **Table S7. Unification of sleep parameters assessed by subjective measurements**

| Sleep measurements | Original parameters | Used as original | United to |
| --- | --- | --- | --- |
| CSHQ | Bedtime resistance | Bedtime resistance |  |
|  | Daytime sleepiness | Daytime sleepiness |  |
|  | Night waking | Night waking |  |
|  | Parasomnias | Parasomnias |  |
|  | Sleep anxiety | Sleep anxiety |  |
|  | Sleep disordered breathing | Sleep disordered breathing |  |
|  | Sleep duration | Sleep duration |  |
|  | Sleep latency | Sleep latency |  |
|  | Total score | Total score |  |
| SDSC | Disorders in initiating and maintaining sleep | Disorders in initiating and maintaining sleep |  |
|  | Disorders of arousal |  | Parasomnias |
|  | Disorders of excessive somnolence |  | Daytime sleepiness |
|  | Sleep breathing disorder |  | Sleep disordered breathing |
|  | Sleep hyperhydrosis | Sleep hyperhydrosis |  |
|  | Sleep-wake transition disorders | Sleep-wake transition disorders |  |
|  | Total score |  | Total score |
| PSQ | Breathing problems |  | Sleep disordered breathing |
|  | Insomnia |  | Sleep latency |
|  | Other sleep problems | Other sleep problems |  |
|  | Periodic movement disorder of sleep | Periodic movement disorder of sleep |  |
|  | Sleepiness |  | Daytime sleepiness |
|  | Snoring | Snoring |  |
|  | Total score |  | Total score |
| Sleep diary | Sleep latency |  | Sleep latency |
|  | Sleep quality | Sleep quality |  |
|  | Total sleep time | Total sleep time |  |
| Sleep log assessment | Sleep duration |  | Sleep duration |
|  | Sleep efficiency | Sleep efficiency |  |
|  | Sleep latency |  | Sleep latency |

Abbreviations: CSHQ, the Children's Sleep Habits Questionnaire; SDSC, the Sleep Disturbance Scale for Children; PSQ, Pediatric Sleep questionnaire

# **eAppendix 9. Result of the study quality assessment (Newcastle-Ottawa scale)**

## **Table S8. Result of the Newcastle-Ottawa scale**

| Author, year | Selection | | | | Comparability | Exposure | | | Total score | Study quality |
| --- | --- | --- | --- | --- | --- | --- | --- | --- | --- | --- |
|  | **Definition of cases** | **Representativeness of the cases** | **Selection of cases** | **Definition of controls** |  | **Ascertainment of exposure** | **Same method of ascertainment** | **Non-response rate** |  |  |
| Allik 2006 | * | * | * | * | * | * | * | * | 8 | **Good** |
| Anders 2012 | * | * | * | * | * | * | * | * | 8 | **Good** |
| Bruni 2007 | * | * | No description was presented about the selection of cases | * | Study did not control for any factor. | * | * | * | 6 | **Poor** |
| CY Tse 2020 | * | * | * | * | * | * | * | * | 8 | **Good** |
| Elia 2000 | * | * | * | No description was presented about the source of controls | * | * | * | Non-response rate was not identical between cases and controls. | 6 | **Good** |
| Harder 2016 | * | * | * | * | * | * | * | * | 8 | **Good** |
| Lambert 2016 | * | * | * | * | ** | * | * | * | 9 | **Good** |
| Malow 2006 | * | * | * | * | ** | * | * | * | 9 | **Good** |
| Miano 2007 | * | * | * | * | * | * | * | * | 8 | **Good** |
| Mutleur 2016 | * | * | * | * | ** | * | * | * | 9 | **Good** |
| Paavonen 2007 | * | * | * | * | Study did not control for any factor. | * | * | * | 7 | **Poor** |
| Pace 2016 | * | * | * | * | * | * | * | * | 8 | **Good** |
| Reynolds 2018 | * | * | * | * | ** | * | * | * | 9 | **Good** |
| Romeo 2021 | * | * | * | * | * | * | * | * | 8 | Good |
| Tessier 2015 | * | * | * | no description about the source of controls | ** | * | * | * | 8 | Good |
| Tyagi 2018 | * | * | * | * | * | * | * | Non-response rate was not identical between cases and controls. | 7 | Good |

# **eAppendix 10. Statistical results of meta-regression analyses – Publication year, mean age of ASD group, Percentage of boys in ASD group**

## **Table S9. Statistical results of meta-regression analyses - Actigraphy**

| Sleep parameters | Meta-regression moderators | k | Coefficient (95% CI) | p value | NA reason |
| --- | --- | --- | --- | --- | --- |
| Sleep efficiency (%) | Publication year | 4 | -0.0057 (-0.1290, 0.1176) | 0.9277 |  |
|  | Mean age of ASD group | 4 | -0.0491 (-0.2560, 0.1578) | 0.6419 |  |
|  | Percentage of boys in ASD group |  |  |  | k<4 |
| Sleep latency (min) | Publication year |  |  |  | k<4 |
|  | Mean age of ASD group |  |  |  | k<4 |
|  | Percentage of boys in ASD group |  |  |  | k<4 |
| Total sleep time (min) | Publication year | 4 | -0.0524 (-0.1599, 0.0551) | 0.3394 |  |
|  | Mean age of ASD group | 4 | 0.0141 (-0.2249, 0.2530) | 0.9081 |  |
|  | Percentage of boys in ASD group |  |  |  | k<4 |
| Wake after sleep onset (min) | Publication year |  |  |  | k<4 |
|  | Mean age of ASD group |  |  |  | k<4 |
|  | Percentage of boys in ASD group |  |  |  | k<4 |

Abbreviations: ASD, autism spectrum disorders; CI, confidence interval; NA, not available; k, the number of studies.

## **Table S10. Statistical results of meta-regression analyses - Polysomnography**

| Sleep parameters | Meta-regression moderators | k | Coefficient (95% CI) | p value | NA reason |
| --- | --- | --- | --- | --- | --- |
| Number of awakenings per hour | Publication year |  |  |  | k<4 |
|  | Mean age of ASD group |  |  |  | k<4 |
|  | Percentage of boys in ASD group |  |  |  | k<4 |
| REM density (no./h REM sleep) | Publication year |  |  |  | k<4 |
|  | Mean age of ASD group |  |  |  | k<4 |
|  | Percentage of boys in ASD group |  |  |  | k<4 |
| REM latency (min) | Publication year | 6 | 0.0338 (-0.0357, 0.1033) | 0.3407 |  |
|  | Mean age of ASD group | 6 | -0.1711 (-0.3753, 0.0330) | 0.1004 |  |
|  | Percentage of boys in ASD group | 5 | 0.0072 (-0.0659, 0.0802) | 0.8475 |  |
| REM sleep (%) | Publication year | 6 | 0.0161 (-0.0416, 0.0737) | 0.585 |  |
|  | Mean age of ASD group | 6 | 0.0584 (-0.1268, 0.2436) | 0.5366 |  |
|  | Percentage of boys in ASD group | 5 | -0.0114 (-0.0606, 0.0378) | 0.6502 |  |
| S1 (%) | Publication year | 6 | 0.0448 (-0.0126, 0.1022) | 0.1259 |  |
|  | Mean age of ASD group | 6 | 0.1198 (-0.0657, 0.3052) | 0.2055 |  |
|  | Percentage of boys in ASD group | 5 | 0.0221 (-0.0272, 0.0714) | 0.3805 |  |
| S2 (%) | Publication year | 6 | 0.0426 (-0.0147, 0.0999) | 0.1446 |  |
|  | Mean age of ASD group | 6 | 0.0447 (-0.1484, 0.2379) | 0.6499 |  |
|  | Percentage of boys in ASD group | 5 | 0.0062 (-0.0432, 0.0556) | 0.8054 |  |
| Slow wave sleep (%) | Publication year | 6 | -0.1196 (-0.1817, -0.0574) | **<0.0005** |  |
|  | Mean age of ASD group | 6 | -0.1755 (-0.5796, 0.2287) | 0.3947 |  |
|  | Percentage of boys in ASD group | 5 | -0.0512 (-0.1222, 0.0198) | 0.1578 |  |
| Sleep efficiency (%) | Publication year | 6 | 0.0410 (-0.0219, 0.1038) | 0.2014 |  |
|  | Mean age of ASD group | 6 | 0.1546 (-0.0405, 0.3497) | 0.1204 |  |
|  | Percentage of boys in ASD group | 5 | 0.0340 (-0.0195, 0.0876) | 0.2132 |  |
| Sleep latency (min) | Publication year | 6 | -0.0374 (-0.0959, 0.0210) | 0.2096 |  |
|  | Mean age of ASD group | 6 | -0.0409 (-0.2574, 0.1755) | 0.7108 |  |
|  | Percentage of boys in ASD group | 5 | -0.0438 (-0.0966, 0.0089) | 0.1029 |  |
| Sleep period time (min) | Publication year |  |  |  | k<4 |
|  | Mean age of ASD group |  |  |  | k<4 |
|  | Percentage of boys in ASD group |  |  |  | k<4 |

Abbreviations: ASD, autism spectrum disorders; CI, confidence interval; NA, not available; k, the number of studies; REM, rapid eye movement.

## **Table S10. Continued**

| Sleep parameters | Meta-regression moderators | k | Coefficient (95% CI) | p value | NA reason |
| --- | --- | --- | --- | --- | --- |
| Stage shift per hour | Publication year |  |  |  | k<4 |
|  | Mean age of ASD group |  |  |  | k<4 |
|  | Percentage of boys in ASD group |  |  |  | k<4 |
| Time in bed (min) | Publication year | 4 | 0.0575 (-0.0133, 0.1282) | 0.1113 |  |
|  | Mean age of ASD group | 4 | -0.0691 (-0.3269, 0.1887) | 0.5992 |  |
|  | Percentage of boys in ASD group | 4 | 0.0012 (-0.1076, 0.1100) | 0.9827 |  |
| Total sleep time (min) | Publication year | 6 | 0.0903 (0.0172, 0.1635) | **<0.05** |  |
|  | Mean age of ASD group | 6 | 0.0011 (-0.3473, 0.3495) | 0.995 |  |
|  | Percentage of boys in ASD group | 5 | -0.0059 (-0.0614, 0.0495) | 0.8336 |  |
| Wake after sleep onset (min) | Publication year | 5 | 0.0577 (-0.0078, 0.1232) | 0.0843 |  |
|  | Mean age of ASD group | 5 | 0.0620 (-0.2086, 0.3327) | 0.6532 |  |
|  | Percentage of boys in ASD group | 5 | 0.0526 (0.0027, 0.1025) | **<0.05** |  |

Abbreviations: ASD, autism spectrum disorders; CI, confidence interval; NA, not available; k, the number of studies; REM, rapid eye movement.

## **Table S11. Statistical results of meta-regression analyses - Actigraphy + Polysomnography**

| Sleep parameters | Meta-regression moderators | k | Coefficient (95% CI) | p value | NA reason |
| --- | --- | --- | --- | --- | --- |
| Sleep efficiency (%) | Publication year | 10 | 0.0188 (-0.0365, 0.0741) | 0.5056 |  |
|  | Mean age of ASD group | 10 | -0.0032 (-0.1325, 0.1261) | 0.9616 |  |
|  | Percentage of boys in ASD group | 8 | 0.0079 (-0.0284, 0.0443) | 0.6696 |  |
| Sleep latency (min) | Publication year | 8 | -0.0588 (-0.1173, -0.0002) | **<0.05** |  |
|  | Mean age of ASD group | 8 | 0.0815 (-0.0099, 0.1728) | 0.0804 |  |
|  | Percentage of boys in ASD group | 7 | -0.0100 (-0.0595, 0.0396) | 0.6935 |  |
| Time in bed (min) | Publication year | 5 | 0.0693 (0.0080, 0.1307) | **<0.01** |  |
|  | Mean age of ASD group | 5 | 0.0465 (-0.272, 0.365) | 0.7749 |  |
|  | Percentage of boys in ASD group | 4 | 0.0012 (-0.1076, 0.1100) | 0.9827 |  |
| Total sleep time (min) | Publication year | 10 | 0.0285 (-0.0399, 0.0970) | 0.414 |  |
|  | Mean age of ASD group | 10 | 0.0074 (-0.1575, 0.1722) | 0.9302 |  |
|  | Percentage of boys in ASD group | 8 | -0.0021 (-0.0428, 0.0386) | 0.9187 |  |

Abbreviation: ASD, autism spectrum disorders; CI, confidence interval; NA, not available; k, the number of studies.

## **Table S12. Statistical results of meta-regression analyses - Subjective measurements**

| Sleep parameters | Meta-regression moderators | k | Coefficient (95% CI) | p value | NA reason |
| --- | --- | --- | --- | --- | --- |
| Daytime sleepiness | Publication year | 7 | -0.0122 (-0.0638, 0.0394) | 0.6432 |  |
|  | Mean age of ASD group | 7 | 0.0698 (0.0232, 0.1164) | **<0.005** |  |
|  | Percentage of boys in ASD group | 6 | -0.0710 (-0.2077, 0.0657) | 0.309 |  |
| Parasomnias | Publication year | 5 | -0.0565 (-0.1061, -0.0069) | **<0.05** |  |
|  | Mean age of ASD group | 5 | 0.0716 (-0.0520, 0.1953) | 0.2561 |  |
|  | Percentage of boys in ASD group | 4 | -0.0601 (-0.3151, 0.1948) | 0.6439 |  |
| Sleep disordered breathing | Publication year | 6 | -0.0530 (-0.1483, 0.0422) | 0.2753 |  |
|  | Mean age of ASD group | 6 | 0.1078 (-0.0308, 0.2464) | 0.1274 |  |
|  | Percentage of boys in ASD group | 5 | -0.0397 (-0.3937, 0.3144) | 0.8263 |  |
| Sleep latency | Publication year | 5 | 0.0387 (-0.0296, 0.1071) | 0.2741 |  |
|  | Mean age of ASD group | 5 | 0.0487 (-0.2358, 0.3332) | 0.7371 |  |
|  | Percentage of boys in ASD group | 4 | -0.0936 (-0.1665, -0.0207) | **<0.05** |  |
| Bedtime resistance | Publication year |  |  |  | k<4 |
|  | Mean age of ASD group |  |  |  | k<4 |
|  | Percentage of boys in ASD group |  |  |  | k<4 |
| Disorders in initiating and maintaining sleep | Publication year |  |  |  | k<4 |
|  | Mean age of ASD group |  |  |  | k<4 |
|  | Percentage of boys in ASD group |  |  |  | k<4 |
| Night waking | Publication year |  |  |  | k<4 |
|  | Mean age of ASD group |  |  |  | k<4 |
|  | Percentage of boys in ASD group |  |  |  | k<4 |
| Sleep anxiety | Publication year |  |  |  | k<4 |
|  | Mean age of ASD group |  |  |  | k<4 |
|  | Percentage of boys in ASD group |  |  |  | k<4 |

Abbreviations: ASD, autism spectrum disorders; CI, confidence interval; NA, not available; k, the number of studies.

| Sleep parameters | Meta-regression moderators | k | Coefficient (95% CI) | p value | NA reason |
| --- | --- | --- | --- | --- | --- |
| Sleep duration | Publication year |  |  |  | k<4 |
|  | Mean age of ASD group |  |  |  | k<4 |
|  | Percentage of boys in ASD group |  |  |  | k<4 |
| Sleep hyperhidrosis | Publication year |  |  |  | k<4 |
|  | Mean age of ASD group |  |  |  | k<4 |
|  | Percentage of boys in ASD group |  |  |  | k<4 |
| Sleep-wake transition disorders | Publication year |  |  |  | k<4 |
|  | Mean age of ASD group |  |  |  | k<4 |
|  | Percentage of boys in ASD group |  |  |  | k<4 |
| Total sleep problem | Publication year | 7 | -0.0569 (-0.1060, -0.0078) | **<0.05** |  |
|  | Mean age of ASD group | 6 | 0.0693 (0.0171, 0.1215) | **<0.01** |  |
|  | Percentage of boys in ASD group | 5 | -0.0784 (-0.2434, 0.0866) | 0.3515 |  |

## **Table S12. Continued**

Abbreviations: ASD, autism spectrum disorders; CI, confidence interval; NA, not available; k, the number of studies.

# **eAppendix 11. Statistical results of the subgroup analyses - Inclusion of Intellectual disability**

## **Table S13. Statistical results of the subgroup analyses(Inclusion of Intellectual disability) - Actigraphy**

| Sleep parameters | Subgroup | k | Meta-analysis | Heterogeneity | | p values | NA reason |
| --- | --- | --- | --- | --- | --- | --- | --- |
|  |  |  | Hedges' g (95% CI) | Q value | *I^2^* (%) |  |  |
| Sleep efficiency (%) | Included | 1 | -1.0694 (-1.8513, -0.2875) |  |  | 0.1164 |  |
|  | Excluded | 3 | -0.3275 (-0.8240, 0.1689) | 5.79 | 65.5 |  |  |
| Sleep latency (min) | NA |  |  |  |  |  | All individual studies excluded ID patients |
| Total sleep time (min) | Included | 1 | -1.3268 (-1.7771, -0.8766) |  |  | **<0.001** |  |
|  | Excluded | 3 | -0.3729 (-0.6867, -0.0592) | 2.78 | 28 |  |  |
| Wake after sleep onset (min) | Included | 1 | 0.7831 (0.4571, 1.1090) |  |  | **<0.005** |  |
|  | Excluded | 1 | 0.0677 (-0.2673, 0.4027) |  |  |  |  |

Abbreviations: CI, confidence interval; ID, intellectual disability; k, the number of studies; NA, not available.

## **Table S14. Statistical results of the subgroup analyses(Inclusion of Intellectual disability) - Polysomnography**

| Sleep parameters | Subgroup | k | Meta-analysis | Heterogeneity | | p values | NA reason |
| --- | --- | --- | --- | --- | --- | --- | --- |
|  |  |  | Hedges' g (95% CI) | Q value | *I^2^* (%) |  |  |
| Number of awakenings per hour | Included | 2 | 0.2226 (-0.6302, 1.0754) | 2.46 | 59.4 | 0.9581 |  |
|  | Excluded | 1 | 0.2664 (-1.1273, 1.6601) |  |  |  |  |
| REM density (no./h REM sleep) |  |  |  |  |  |  | All individual studies excluded ID patients |
| REM latency (min) | Included | 2 | -0.7620 (-1.3088, -0.2151) | 0 | 0 | **<0.05** |  |
|  | Excluded | 4 | -0.0161 (-0.4008, 0.3687) | 2.65 | 0 |  |  |
| REM sleep (%) | Included | 2 | -0.3223 (-0.8515, 0.2069) | 0.01 | 0 | 0.9854 |  |
|  | Excluded | 4 | -0.3284 (-0.7161, 0.0593) | 2.94 | 0 |  |  |
| S1 (%) | Included | 2 | 0.3085 (-0.2204, 0.8374) | 0 | 0 | 0.5105 |  |
|  | Excluded | 4 | 0.0885 (-0.2983, 0.4753) | 4.39 | 31.7 |  |  |
| S2 (%) | Included | 2 | -0.4534 (-0.9864, 0.0796) | 0 | 0 | 0.3205 |  |
|  | Excluded | 4 | -0.1198 (-0.5061, 0.2665) | 3.67 | 18.2 |  |  |
| Slow wave sleep (%) | Included | 2 | 0.3412 (-0.9681, 1.6506) | 0.01 | 0 | 0.8102 |  |
|  | Excluded | 4 | 0.1430 (-0.8074, 1.0933) | 18.44 | 83.7 |  |  |
| Sleep efficiency (%) | Included | 2 | -0.5400 (-1.2098, 0.1298) | 0.06 | 0 | 0.8003 |  |
|  | Excluded | 4 | -0.6477 (-1.1458, -0.1496) | 5.97 | 49.7 |  |  |
| Sleep latency (min) | Included | 2 | 0.5025 (-0.1162, 1.1212) | 0.03 | 0 | 0.7429 |  |
|  | Excluded | 4 | 0.6316 (0.1710, 1.0921) | 4.59 | 34.6 |  |  |
| Sleep period time (min) | Included | 2 | -1.0499 (-1.6159, -0.4840) | 0.06 | 0 | 0.3381 |  |
|  | Excluded | 1 | -1.7019 (-2.9100, -0.4938) |  |  |  |  |
| Stage shift per hour | Included | 2 | 0.3533 (-0.1774, 0.8841) | 0.33 | 0 | 0.8404 |  |
|  | Excluded | 1 | 0.0059 (-1.0255, 1.0373) |  |  |  |  |
| Time in bed (min) | Included | 2 | -0.8555 (-1.4790, -0.2320) | 0.01 | 0 | 0.8404 |  |
|  | Excluded | 2 | -0.7647 (-1.3912, -0.1382) | 2.72 | 63.2 |  |  |
| Total sleep time (min) | Included | 2 | -1.1004 (-2.0046, -0.1962) | 0.04 | 0 | 0.2745 |  |
|  | Excluded | 4 | -0.4800 (-1.1283, 0.1682) | 9.43 | 68.2 |  |  |
| Wake after sleep onset (min) | Included | 2 | 0.1653 (-0.6163, 0.9469) | 0.03 | 0 | 0.9614 |  |
|  | Excluded | 3 | 0.1399 (-0.5262, 0.8060) | 5.99 | 66.6 |  |  |

Abbreviations: CI, confidence interval; k, the number of studies; NA, not available; REM, rapid eye movement.

## **Table S15. Statistical results of the subgroup analyses(Inclusion of Intellectual disability) - Actigraphy + Polysomnography**

| Sleep parameters | Subgroup | k | Meta-analysis | Heterogeneity | | p values | NA reason |
| --- | --- | --- | --- | --- | --- | --- | --- |
|  |  |  | Hedges' g (95% CI) | Q value | *I^2^* (%) |  |  |
| Sleep efficiency (%) | Included | 3 | -0.8040 (-1.2936, -0.3144) | 2.81 | 28.7 | 0.27 |  |
|  | Excluded | 7 | -0.4692 (-0.8072, -0.1312) | 13.22 | 54.6 |  |  |
| Sleep latency (min) | Included | 2 | 0.504947 (-0.22, 1.2343) | 0.03 | 0 | 0.7724 |  |
|  | Excluded | 6 | 0.6271 (0.2327, 1.0215) | 13.64 | 63.3 |  |  |
| Time in bed (min) | Included | 2 | -0.8568 (-1.4092, -0.3044) | 0.01 | 0 | 0.2816 |  |
|  | Excluded | 3 | -0.4788 (-0.8891, -0.0685) | 4.49 | 55.5 |  |  |
| Total sleep time (min) | Included | 3 | -1.2435 (-1.6061, -0.8810) | 0.51 | 0 | **<0.0005** |  |
|  | Excluded | 7 | -0.3907 (-0.6441, -0.1374) | 12.21 | 50.9 |  |  |

Abbreviations: CI, confidence interval; k, the number of studies; NA, not available.

## **Table S16. Statistical results of the subgroup analyses(Inclusion of Intellectual disability) - Subjective measurements**

| Sleep parameters | Subgroup | k | Meta-analysis | Heterogeneity | | p values | NA reason |
| --- | --- | --- | --- | --- | --- | --- | --- |
|  |  |  | Hedges' g (95% CI) | Q value | *I^2^* (%) |  |  |
| Daytime sleepiness | Included | 2 | 0.6319 (0.2505, 1.0133) | 4.12 | 75.7 | 0.3363 |  |
|  | Excluded | 5 | 0.4000 (0.1260, 0.6794) | 5.49 | 27.2 |  |  |
| Parasomnias | Included | 1 | 0.1782 (-0.7348, 1.0911) |  |  | 0.7056 |  |
|  | Excluded | 4 | 0.3808 (-0.1399, 0.9015) | 14.47 | 79.3 |  |  |
| Sleep disordered breathing | Included | 2 | 0.4525 (-0.4000, 1.3050) | 14.34 | 93 | 0.4487 |  |
|  | Excluded | 4 | 0.0377 (-0.6141, 0.6895) | 21.39 | 86 |  |  |
| Sleep latency | Included | 2 | 1.5322 (1.2610, 1.8033) | 0.18 | 0 | **<0.005** |  |
|  | Excluded | 3 | 0.7526 (0.3609, 1.1443) | 1.14 | 0 |  |  |
| Bedtime resistance |  |  |  |  |  |  | All individual studies excluded ID patients |
| Disorders in initiating and maintaining sleep | Included | 1 | 0.4058 (0.1002, 0.7114)) |  |  | **<0.001** |  |
|  | Excluded | 2 | 1.0733 (0.8345, 1.3121) | 0.62 | 0 |  |  |
| Night waking |  |  |  |  |  |  | All individual studies excluded ID patients |
| Sleep anxiety |  |  |  |  |  |  | All individual studies excluded ID patients |
| Sleep duration | Included | 1 | -0.7545 (-3.7531, 2.2441) |  |  | 0.4473 |  |
|  | Excluded | 2 | 0.6898 (-1.5203, 2.8999) | 11 | 90.9 |  |  |
| Sleep hyperhidrosis | Included | 1 | 0.4689 (0.0346, 0.9033) |  |  | 0.9099 |  |
|  | Excluded | 2 | 0.5000 (0.1820, 0.8179) | 1.84 | 45.6 |  |  |
| Sleep-wake transition disorders | Included | 1 | -0.0240 (-0.4139, 0.3659) |  |  | **<0.001** |  |
|  | Excluded | 2 | 0.8001 (0.5076, 1.0926) | 1.53 | 34.5 |  |  |
| Total sleep problem | Included | 3 | 0.8074 (0.3658, 1.2491) | 11.87 | 83.2 | 0.66 |  |
|  | Excluded | 4 | 0.9506 (0.4902, 1.4109) | 7.4 | 59.5 |  |  |

Abbreviations: CI, confidence interval; ID, intellectual disability; NA, not available; k, the number of studies.

# **eAppendix 12. Statistical results of the subgroup analyses - Questionnaires for sleep (Subjective measurement)**

**Table S17. Statistical results of the subgroup analyses - Questionnaires for sleep (Subjective measurement)**

| Sleep parameters | Subgroup | k | Meta-analysis | Heterogeneity | | p values | NA reason |
| --- | --- | --- | --- | --- | --- | --- | --- |
|  |  |  | Hedges' g (95% CI) | Q value | *I^2^* (%) |  |  |
| Daytime sleepiness | CSHQ | 3 | 0.2188 (-0.0740, 0.5117) | 2.26 | 11.3 | **<0.05** |  |
|  | SDSC | 3 | 0.4397 (0.2575, 0.6219) | 1.69 | 0 |  |  |
|  | PSQ | 1 | 0.9084 (0.5256, 1.2912) |  |  |  |  |
| Parasomnias | CSHQ | 2 | 0.5918 (-0.2075, 1.3911) | 2.13 | 53.2 | 0.4379 |  |
|  | SDSC | 3 | 0.2269 (-0.2328, 0.6865) | 10.82 | 81.5 |  |  |
| Sleep disordered breathing | CSHQ | 2 | 0.3857 (-0.4865, 1.2578) | 2.29 | 56.3 | 0.1421 |  |
|  | SDSC | 3 | -0.1466 (-0.6934, 0.4003) | 17.4 | 88.5 |  |  |
|  | PSQ | 1 | 0.9336 (-0.0364, 1.9036) |  |  |  |  |
| Sleep latency | Sleep diary | 1 | 0.7928 (0.2267, 1.3588) |  |  | **<0.05** |  |
|  | CSHQ | 2 | 0.6980 (0.0620, 1.3340) | 1.08 | 7.3 |  |  |
|  | PSQ | 1 | 1.6006 (1.1145, 2.0867) |  |  |  |  |
|  | Sleep log assessment | 1 | 1.4831 (1.0512, 1.9149) |  |  |  |  |
| Bedtime resistance |  |  |  |  |  |  | All individual studies used CSHQ |
| Disorders in initiating and maintaining sleep |  |  |  |  |  |  | All individual studies used SDSC |
| Night waking |  |  |  |  |  |  | All individual studies used CSHQ |
| Sleep anxiety |  |  |  |  |  |  | All individual studies used CSHQ |
| Sleep duration | CSHQ | 2 | 0.6898 (-1.5203, 2.8999) | 11 | 90.9 | 0.4473 |  |
|  | Sleep log assessment | 1 | -0.7545 (-3.7531, 2.2441) |  |  |  |  |
| Sleep hyperhidrosis |  |  |  |  |  |  | All individual studies used SDSC |
| Sleep-wake transition disorders |  |  |  |  |  |  | All individual studies used SDSC |
| Total sleep problem | CSHQ | 3 | 0.6834 (0.2531, 1.1137) | 5.95 | 66.4 | 0.2664 |  |
|  | SDSC | 3 | 0.8383 (0.4852, 1.1914) | 4.86 | 58.8 |  |  |
|  | PSQ | 1 | 1.3290 (0.6765, 1.9814) |  |  |  |  |

Abbreviations: CI, confidence interval; CSHQ, Children's Sleep Habits Questionnaire; k, the number of studies; NA, not available; PSQ, Pediatric Sleep questionnaire; SDSC, Sleep Disturbance Scale for Children.

# **eAppendix 13. Funnel plot, p-curve analysis plot, and influence analysis plot**

## **13.1. Actigraphy**

### **Figure S2. Funnel plot and influence analysis plot for Sleep efficiency (%)**

| 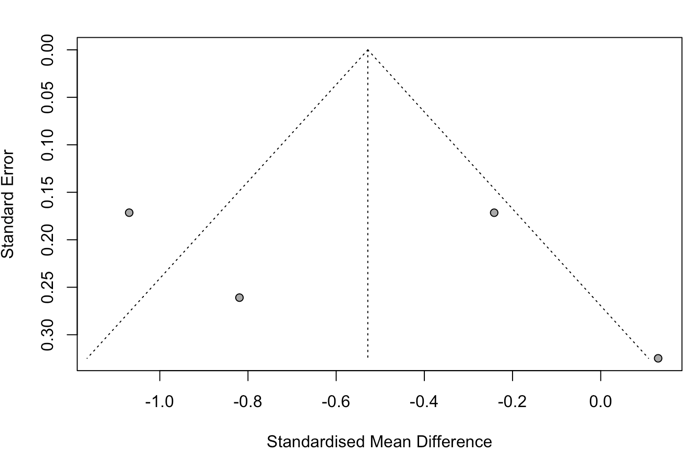 | 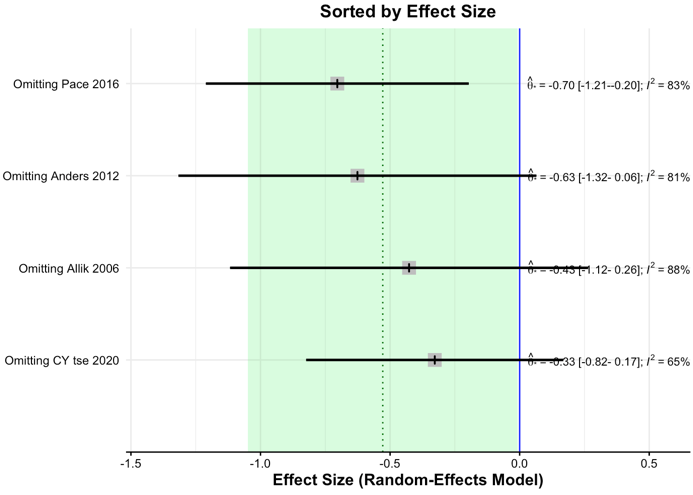 |
| --- | --- |

### **Figure S3. Funnel plot for Sleep latency(min)**

| 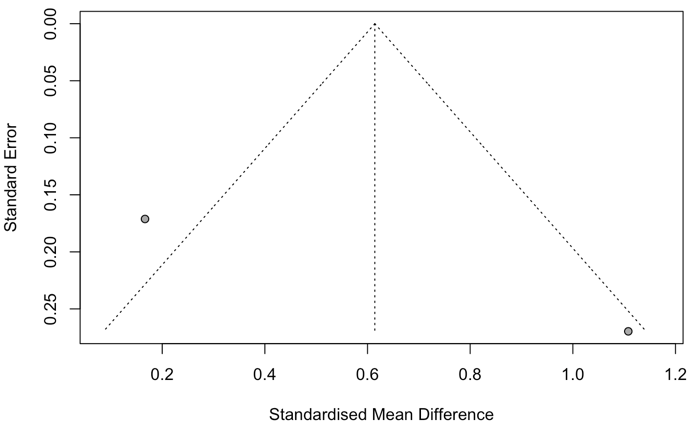 | - |
| --- | --- |

### **Figure S4. Funnel plot and influence analysis plot for Total sleep time (min)**

| 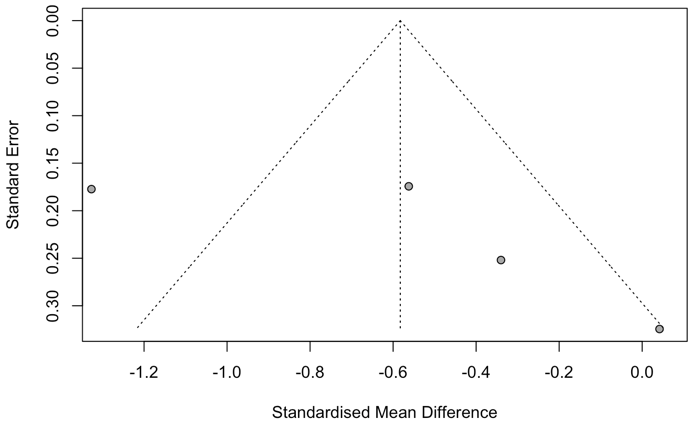 | 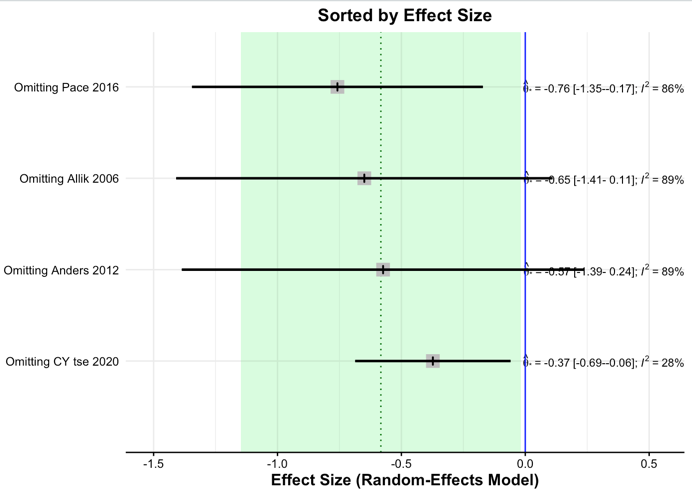 |
| --- | --- |

### **Figure S5. Funnel plot for Wake after sleep onset (min)**

| 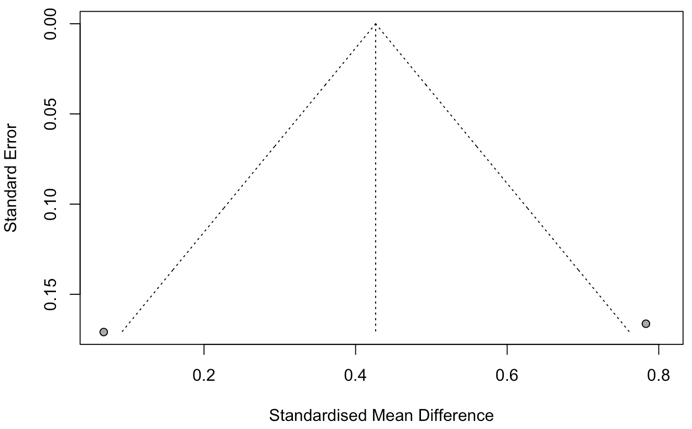 | - |
| --- | --- |

## **13.2. Polysomnography**

### **Figure S6. Funnel plot and influence analysis plot for Number of awakenings per hour**

| 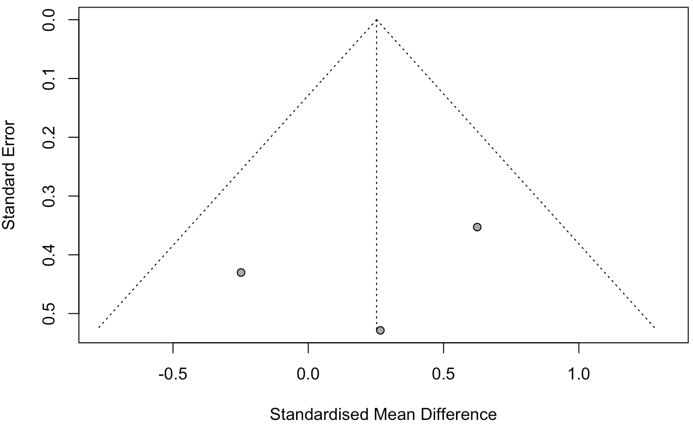 | 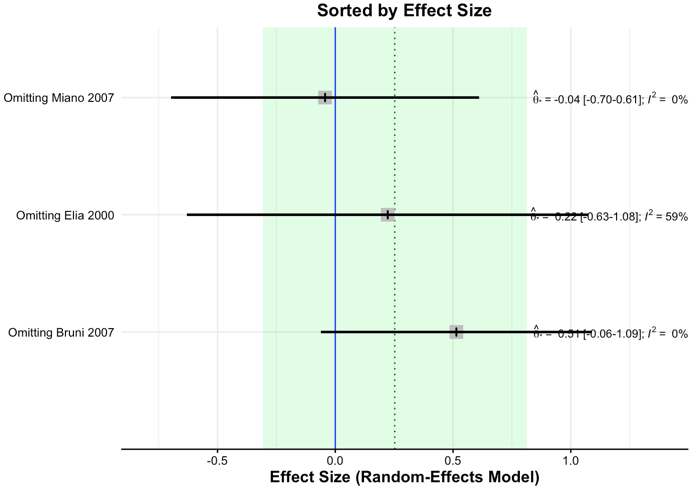 |
| --- | --- |

### **Figure S7. Funnel plot for REM density (no./h REM sleep)**

| 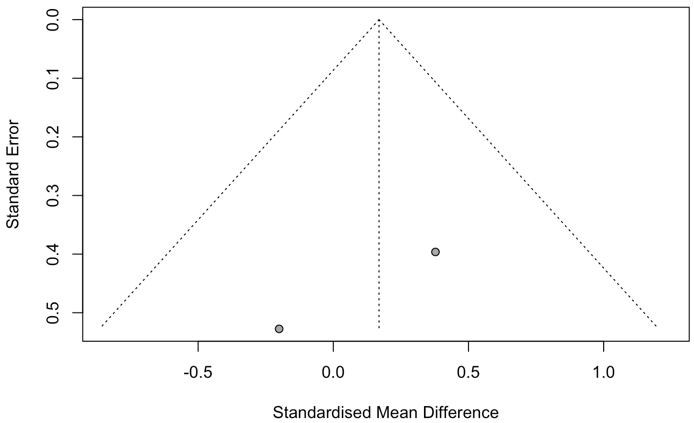 |  |
| --- | --- |

### **Figure S8. Funnel plot and influence analysis plot for REM latency (min)**

| 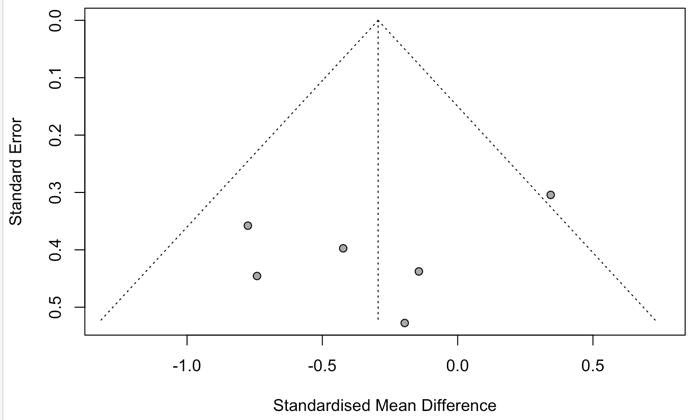 | 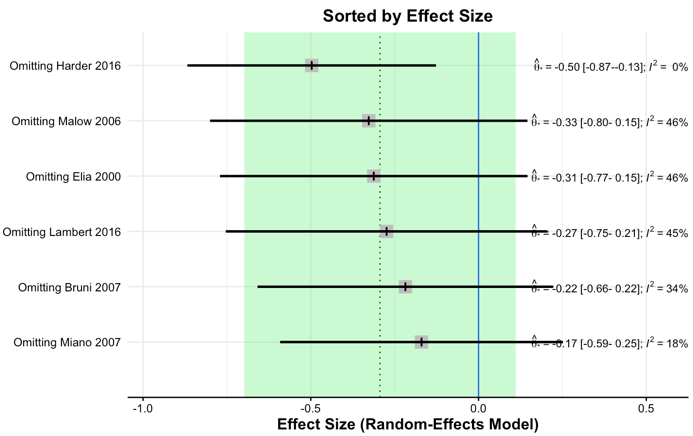 |
| --- | --- |

### **Figure S9. Funnel plot and influence analysis plot for REM sleep (%)**

| 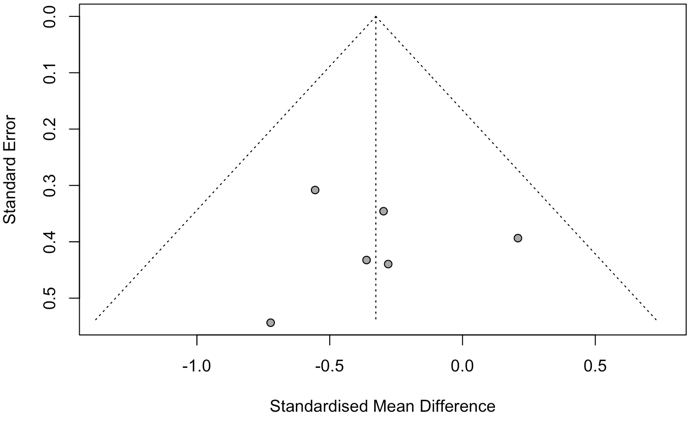 | 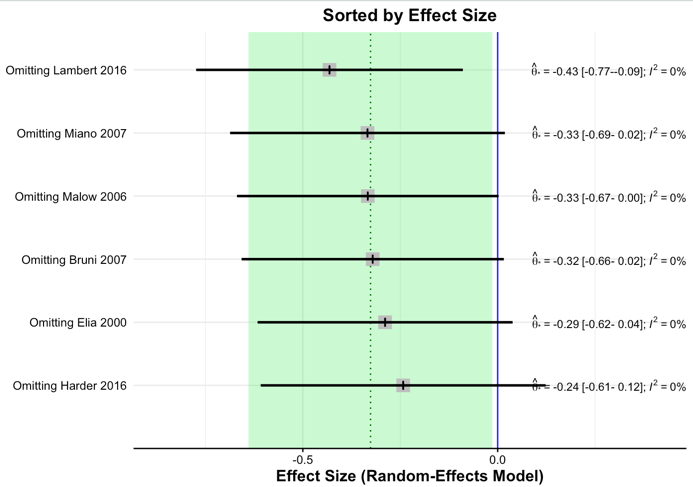 |
| --- | --- |

### **Figure S10. Funnel plot and influence analysis plot for S1 (%)**

| 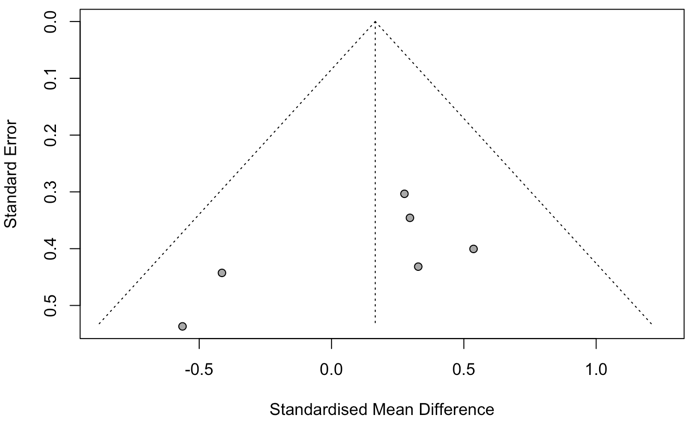 | 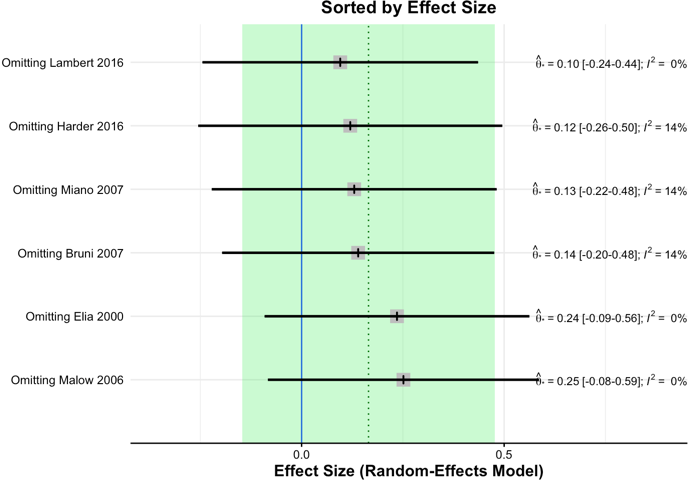 |
| --- | --- |

### **Figure S11. Funnel plot and influence analysis plot for S2 (%)**

| 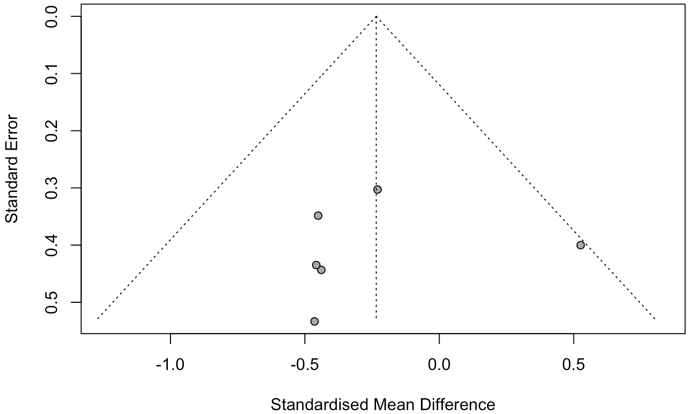 | 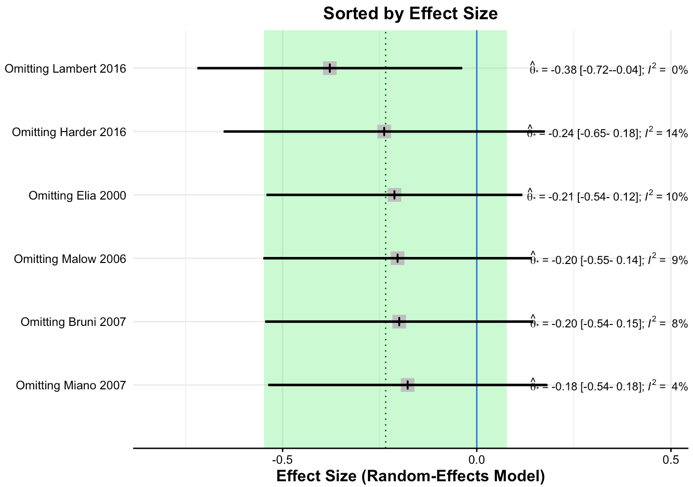 |
| --- | --- |

### **Figure S12. Funnel plot and influence analysis plot for Slow wave sleep (%)**

| 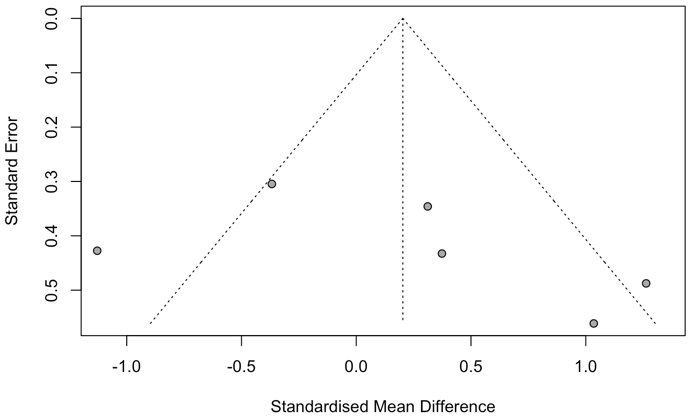 | 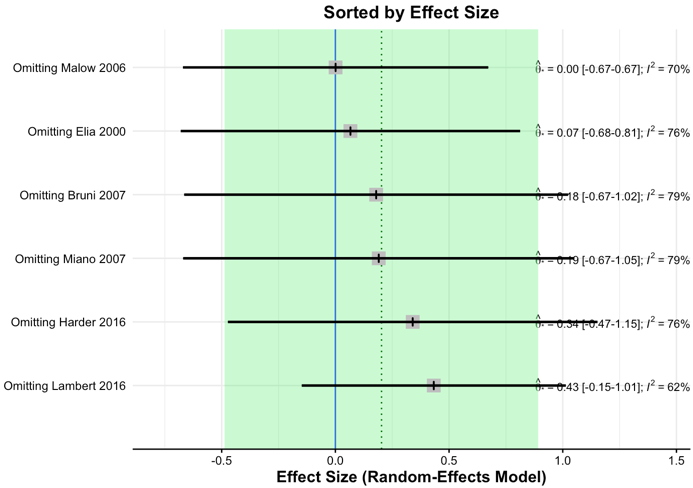 |
| --- | --- |

### **Figure S13. Funnel plot and influence analysis plot for Sleep efficiency (%)**

| 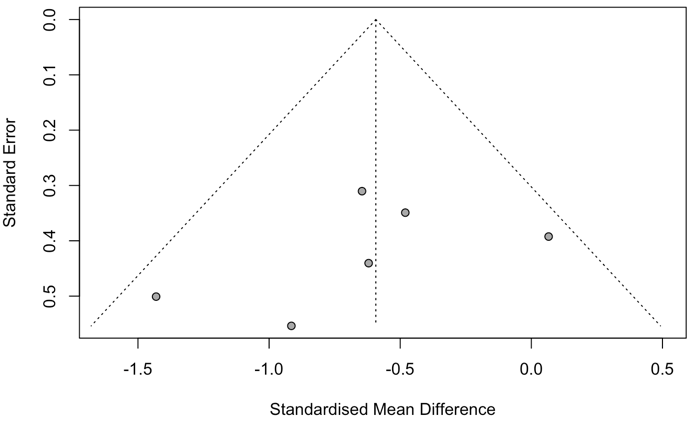 | 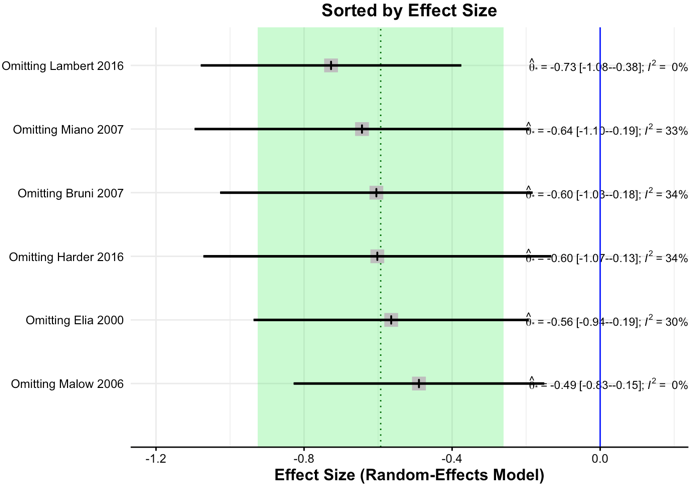 |
| --- | --- |

|  |  |
| --- | --- |

### **Figure S14. Funnel plot and influence analysis plot for Sleep latency (min)**

| 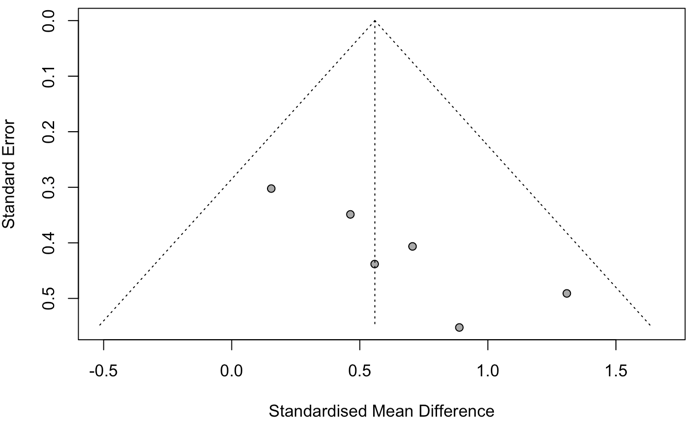 | 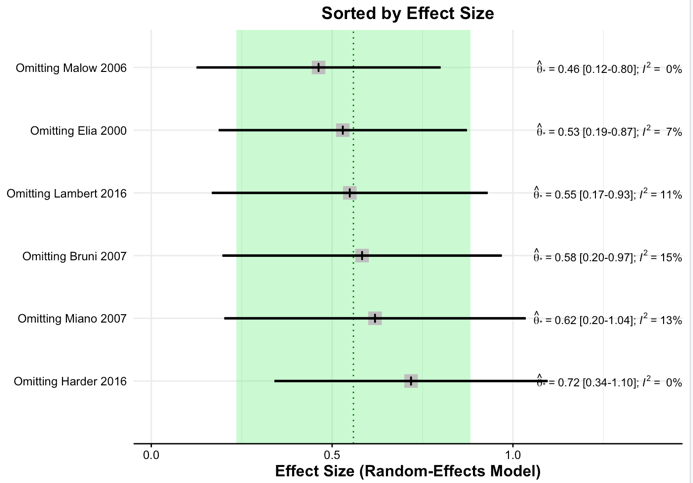 |
| --- | --- |

### **Figure S15. Funnel plot, p-curve plot, and influence analysis plot for Sleep period time (min)**

| 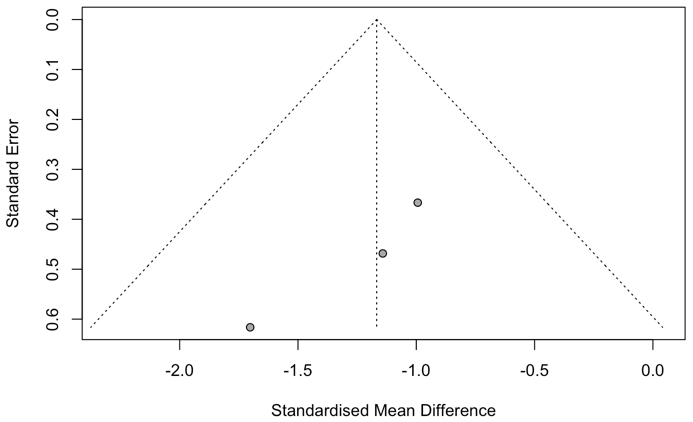 | 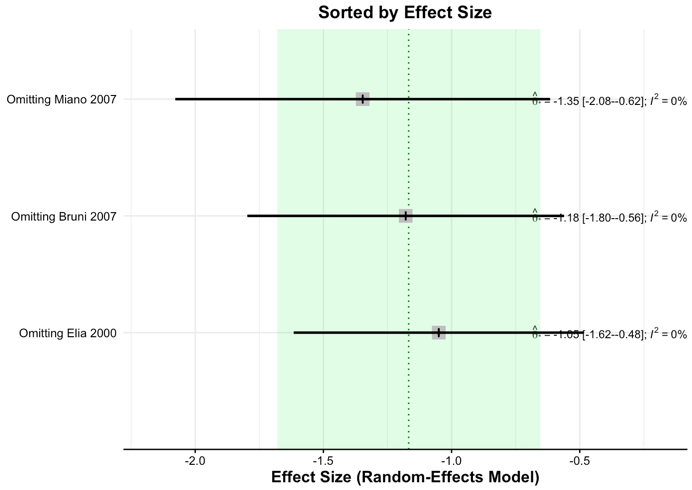 |
| --- | --- |
| **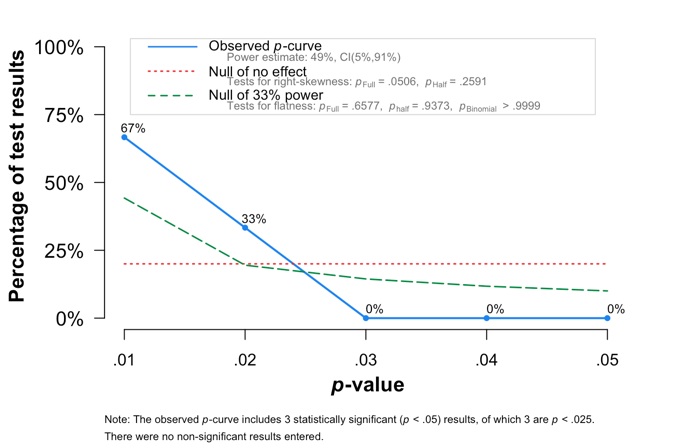** |  |

### **Figure S16. Funnel plot and influence analysis plot for Stage shift per hour**

| 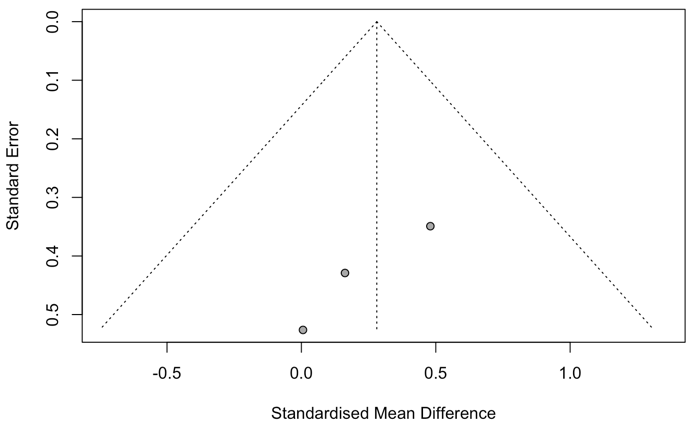 | 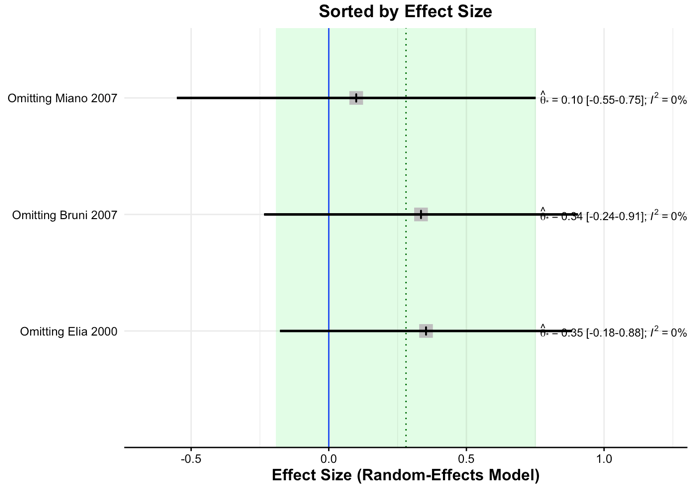 |
| --- | --- |

### **Figure S17. Funnel plot and influence analysis plot for Time in bed (min)**

| 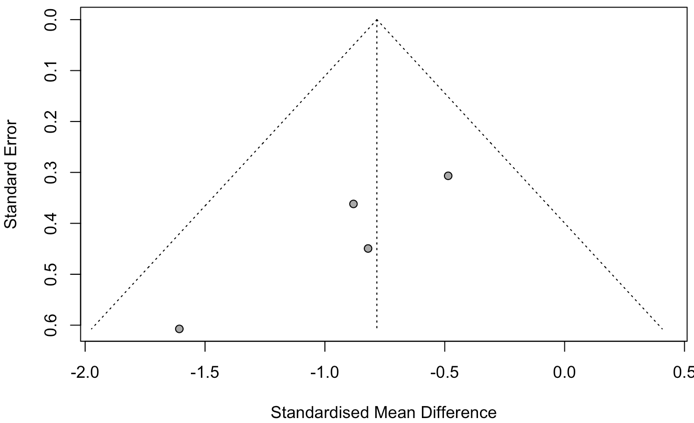 | 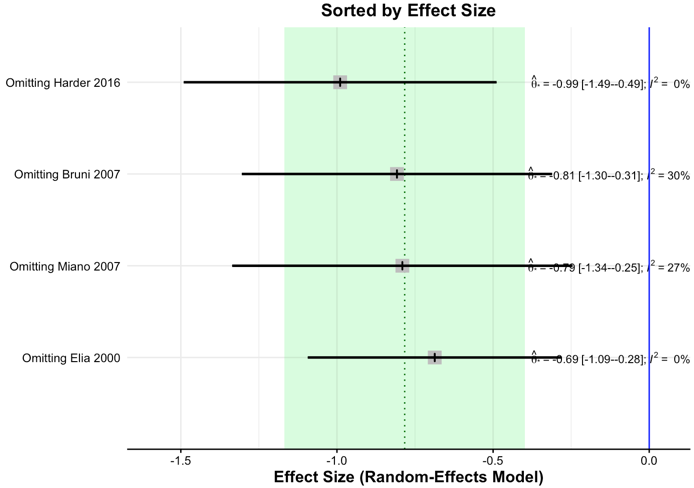 |
| --- | --- |

### **Figure S18. Funnel plot, p-curve plot, and influence analysis plot for Total sleep time (min)**

| 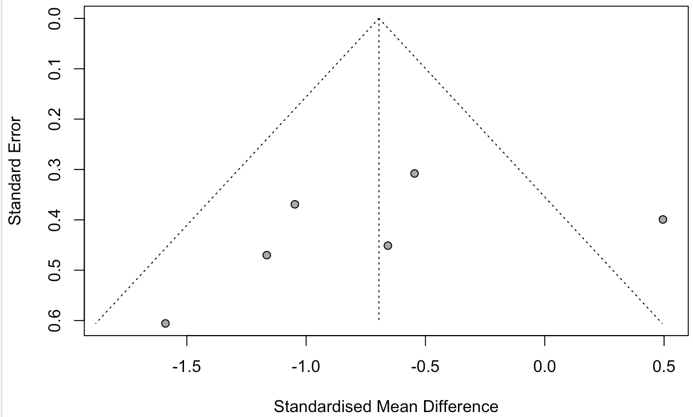 | 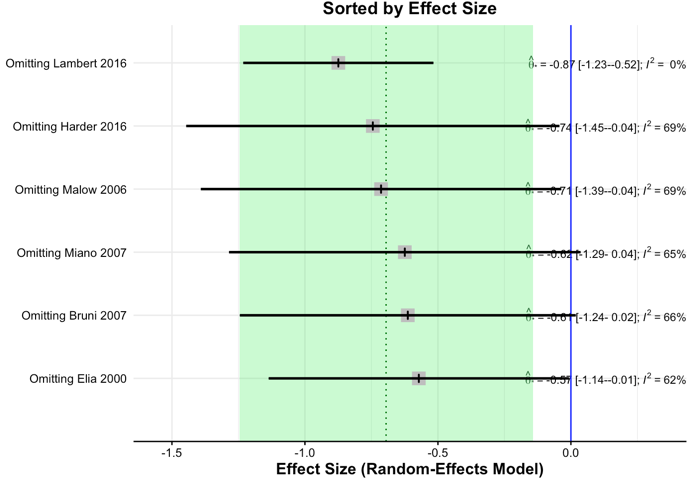 |
| --- | --- |
| 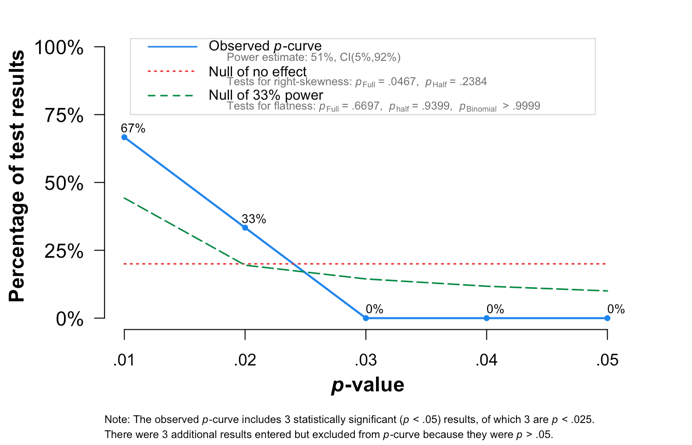 |  |

### **Figure S19. Funnel plot and influence analysis plot for Wake after sleep onset (min)**

| 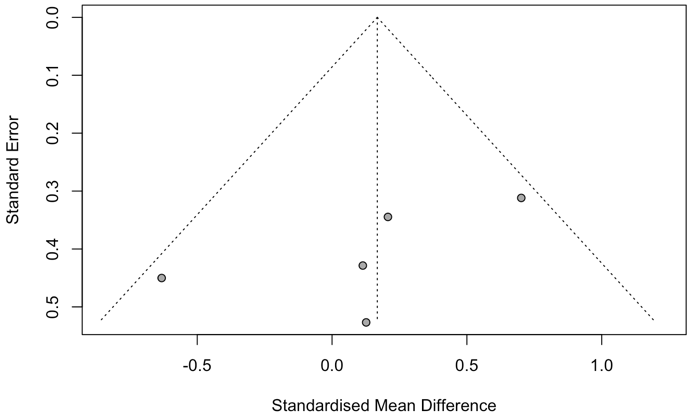 | 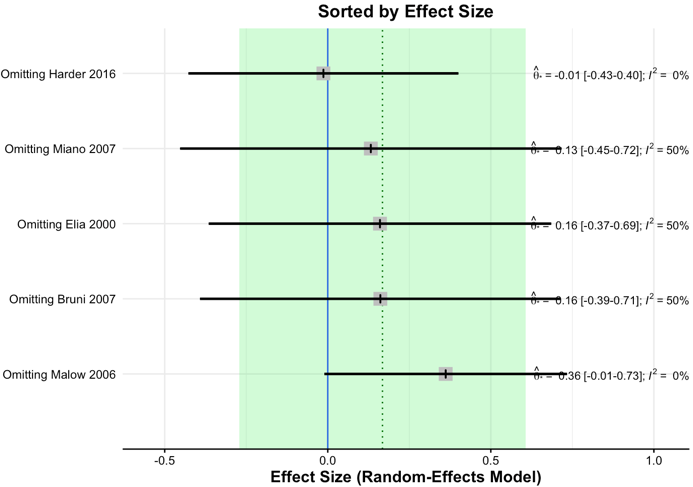 |
| --- | --- |

## **13.3. Actigraphy + Polysomnography**

### **Figure S20. Funnel plot, p-curve plot, and influence analysis plot for Sleep efficiency**

| 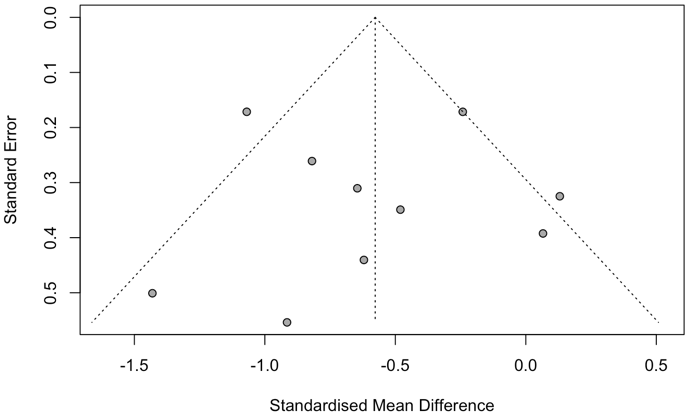 | 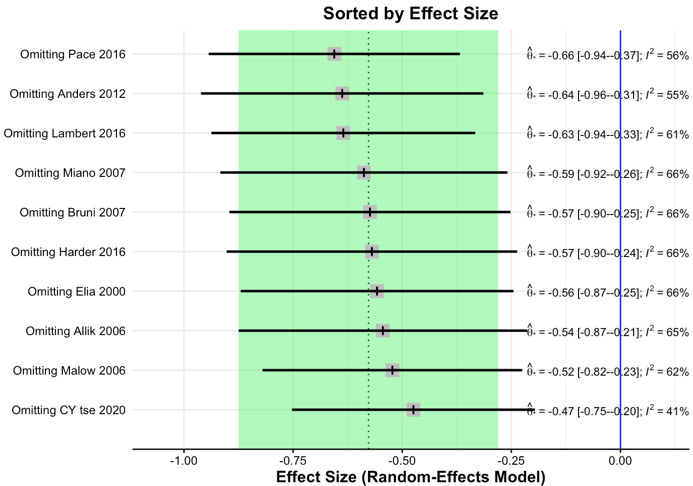 |
| --- | --- |
| 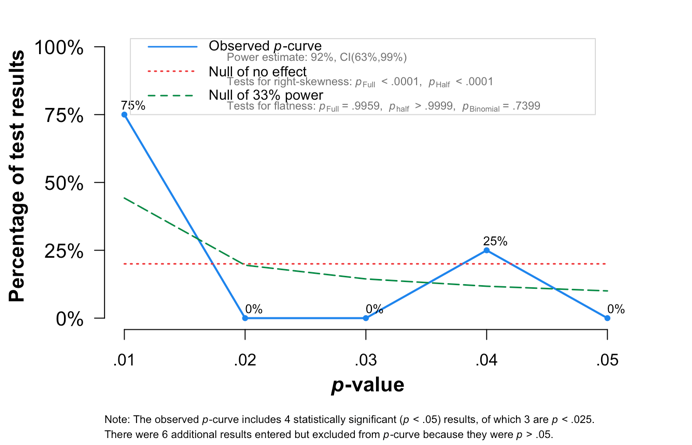 |  |

### **Figure S21. Funnel plot and influence analysis plot for Sleep latency (min)**

| 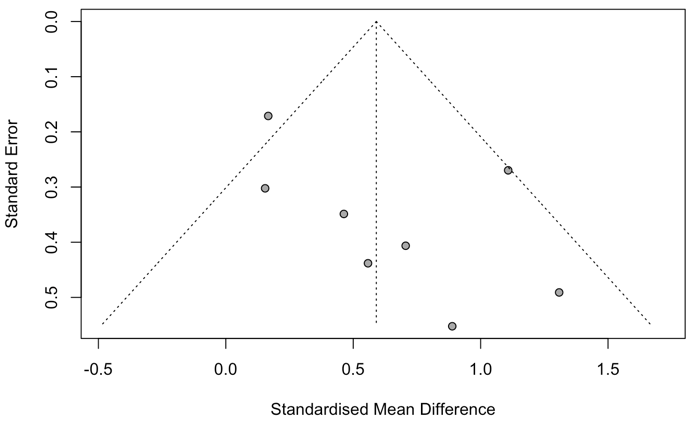 | 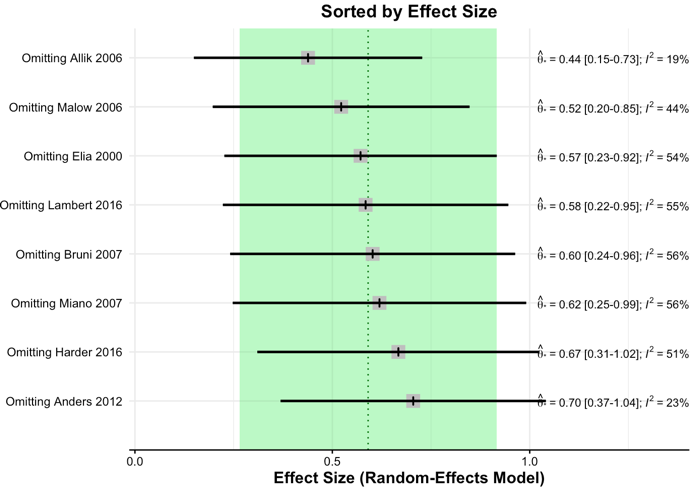 |
| --- | --- |

### **Figure S22. Funnel plot and influence analysis plot for Time in bed (min)**

| 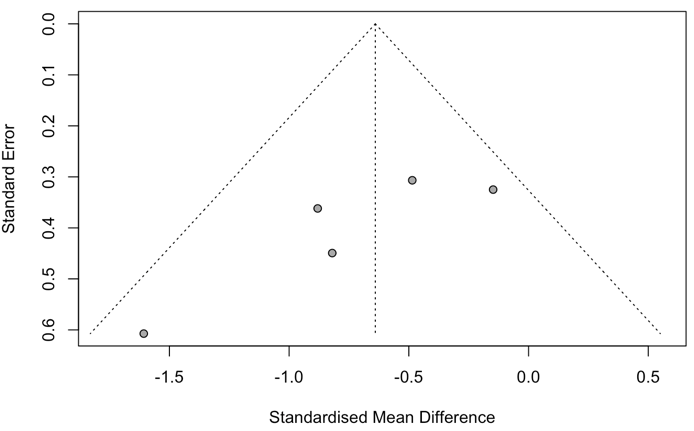 | 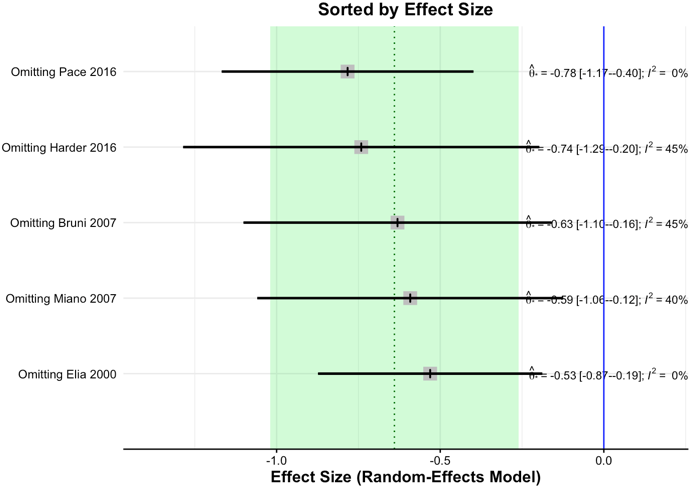 |
| --- | --- |

### **Figure S23. Funnel plot, p-curve plot, and influence analysis plot for Total sleep time (min)**

| 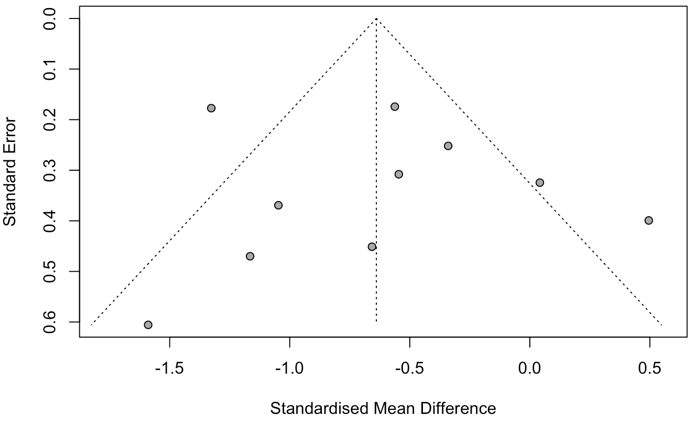 | 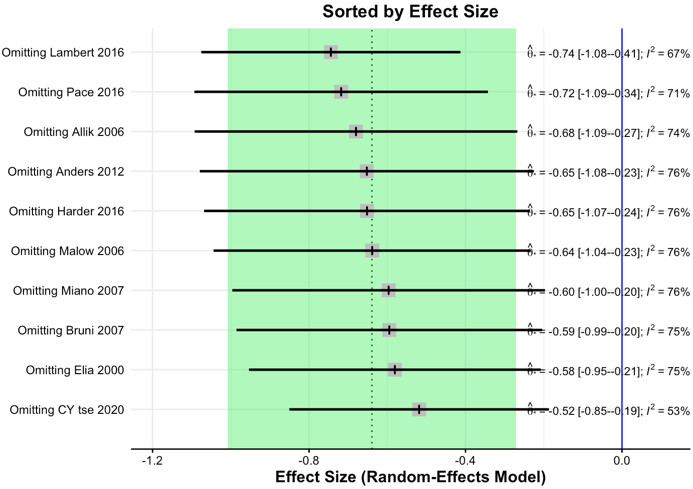 |
| --- | --- |
| 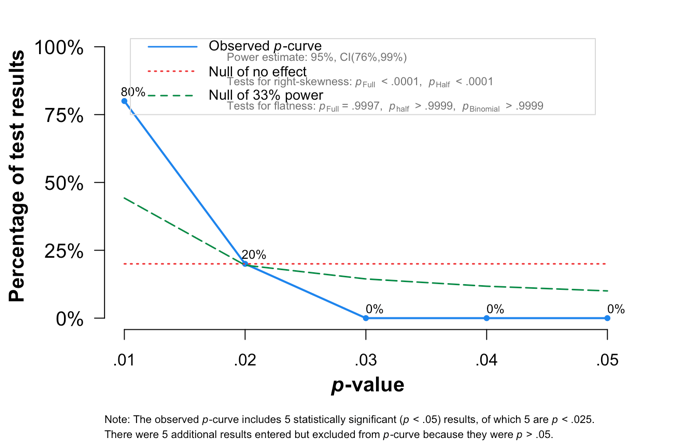 |  |

## **13.4. Subjective measurement**

### **Figure S24. Funnel plot, and p-curve plot, and influence analysis plot for Daytime sleepiness**

| 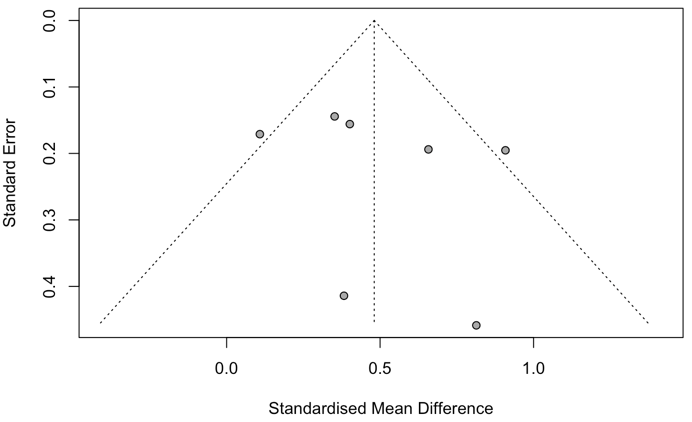 | 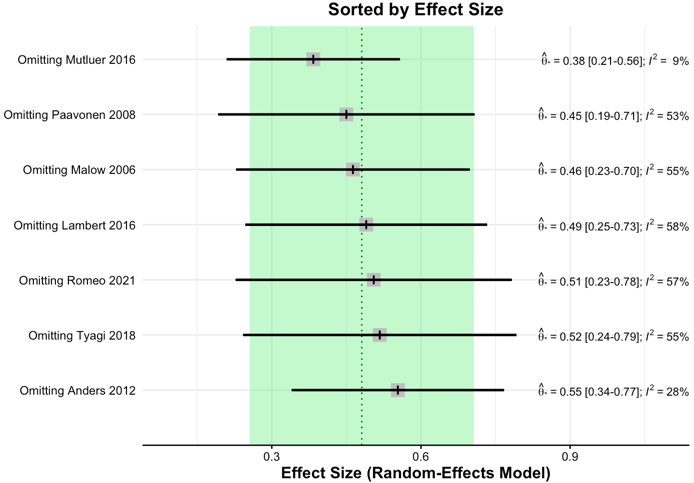 |
| --- | --- |
| 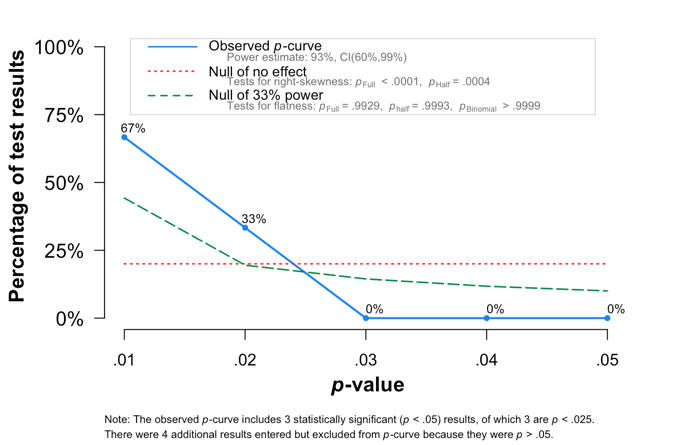 |  |

### **Figure S25. Funnel plot and influence analysis plot for Parasomnias**

| 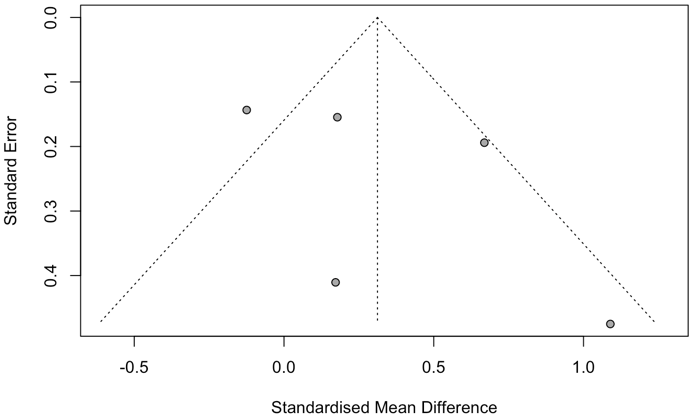 | 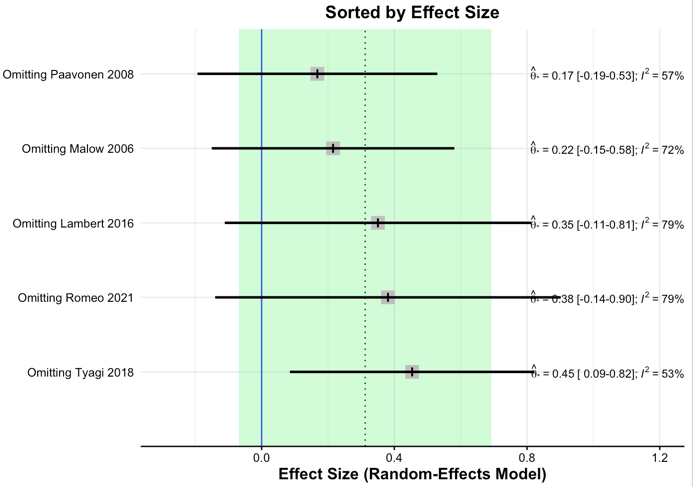 |
| --- | --- |

### **Figure S26. Funnel plot and influence analysis plot for Sleep disordered breathing**

| 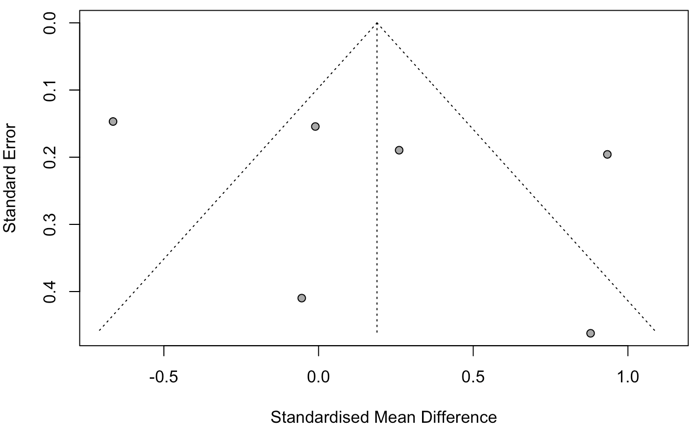 | 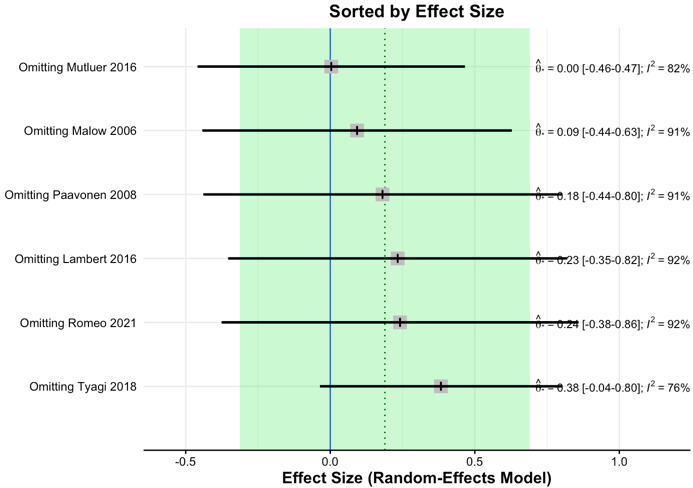 |
| --- | --- |

### **Figure S27. Funnel plot, p-curve plot, and influence analysis plot for Sleep latency**

| 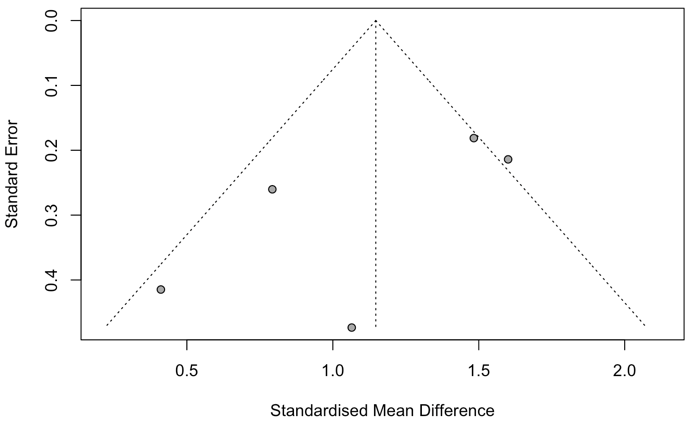 | 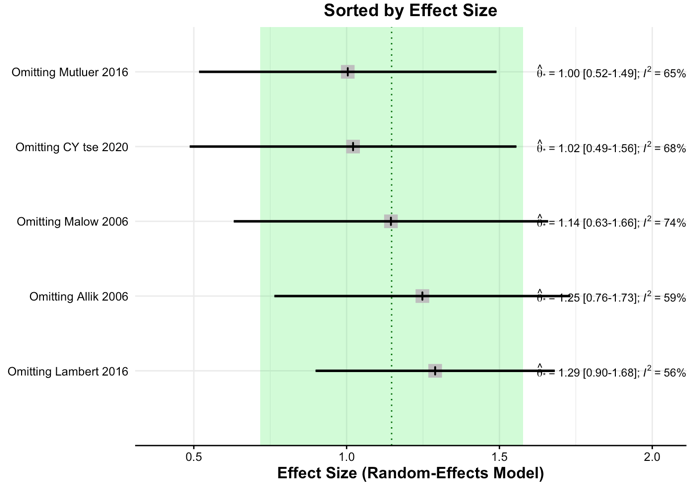 |
| --- | --- |
| 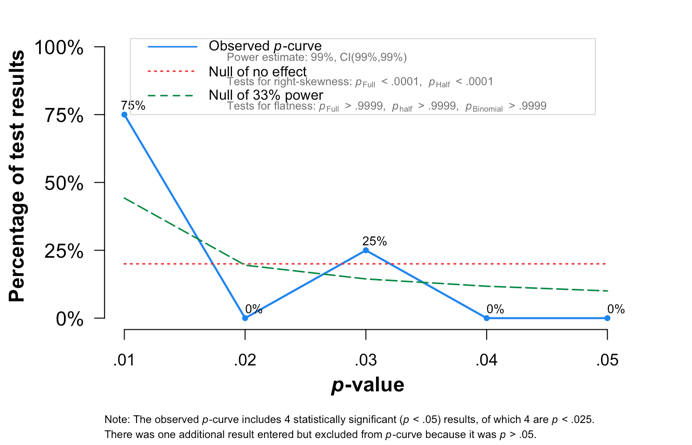 |  |

### **Figure S28. Funnel plot for Bedtime resistance**

| 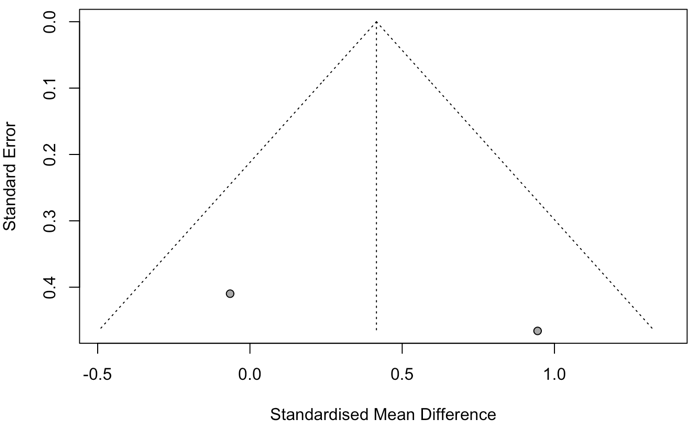 | - |
| --- | --- |

### **Figure S29. Funnel plot, and p-curve plot, and influence analysis plot for Disorders in initiating and maintaining sleep**

| 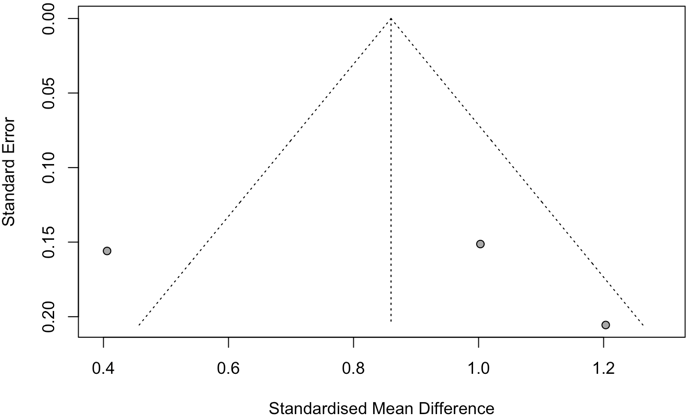 | 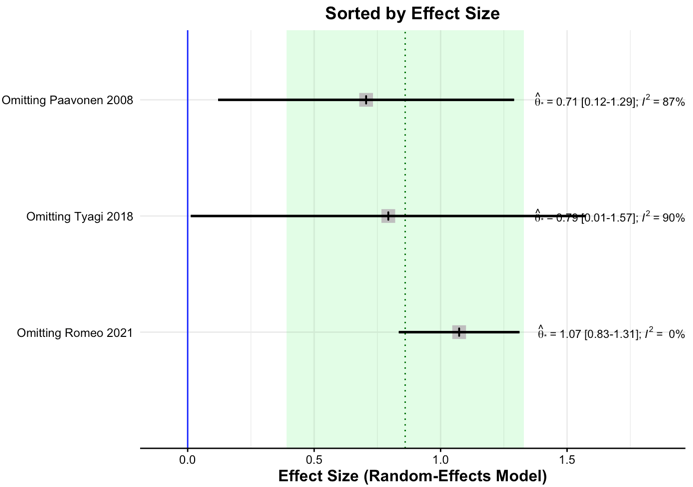 |
| --- | --- |
| 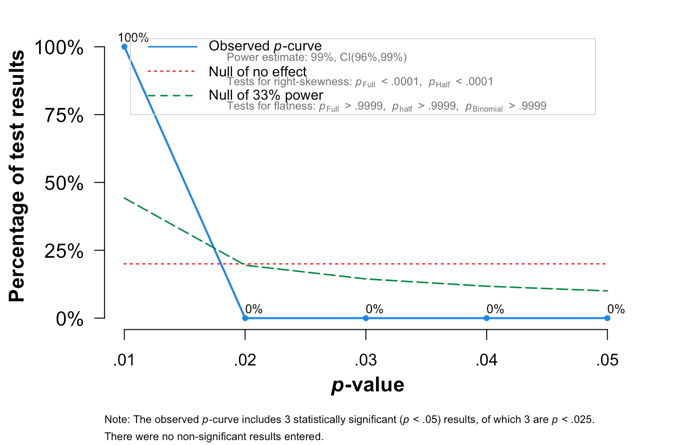 |  |

### **Figure S30. Funnel plot for Night waking**

| 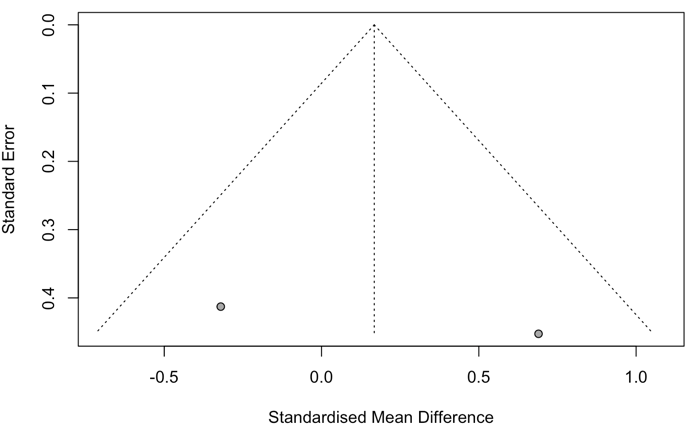 |  |
| --- | --- |

### **Figure S31. Funnel plot for Sleep anxiety**

| 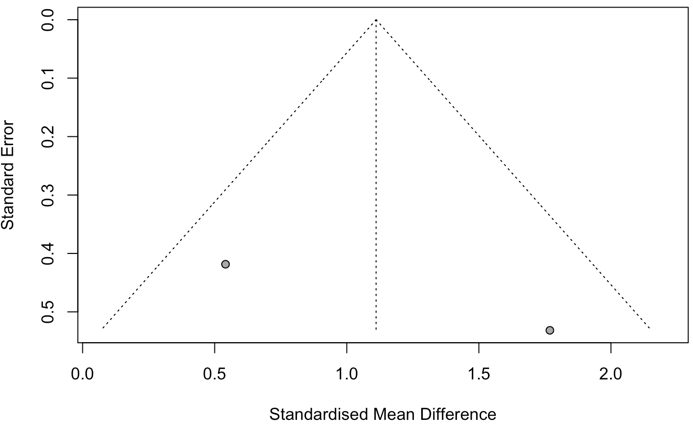 |  |
| --- | --- |

### **Figure S32. Funnel plot and influence analysis plot for Sleep duration**

| 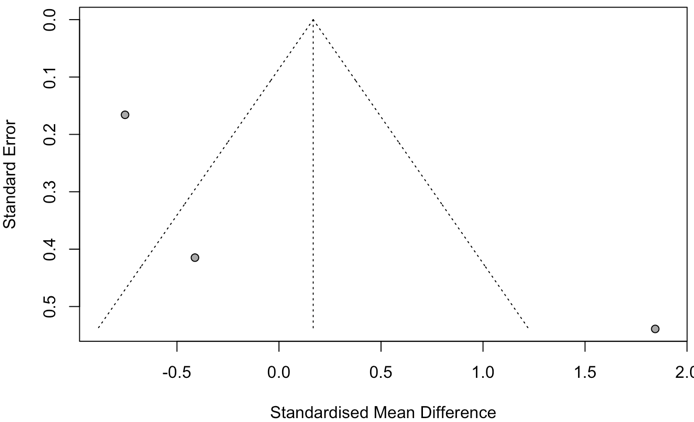 | 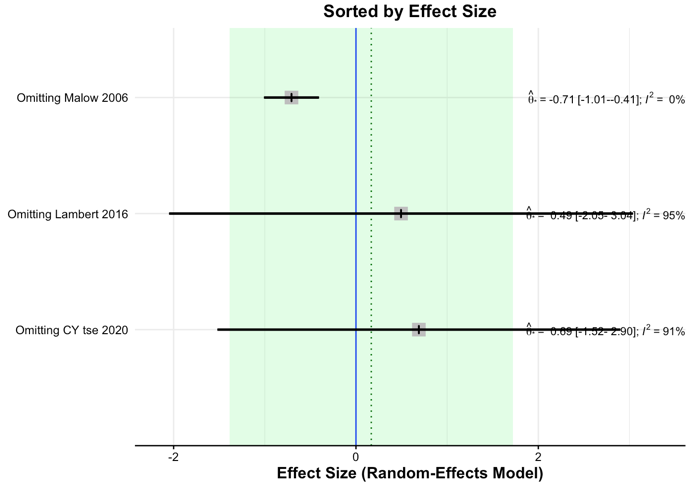 |
| --- | --- |

### **Figure S33. Funnel plot, and p-curve plot, and influence analysis plot for Sleep hyperhidrosis**

| 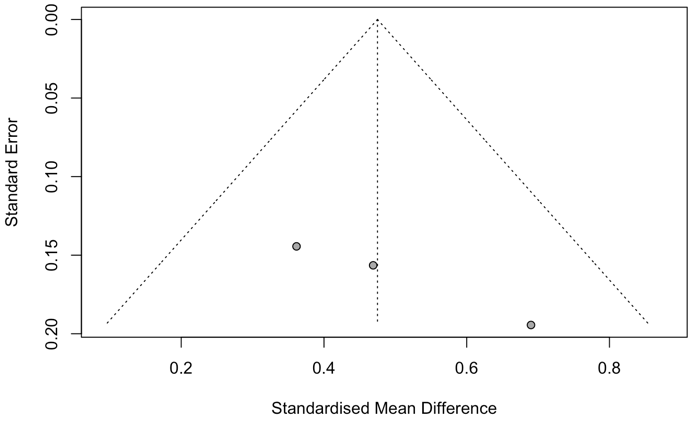 | 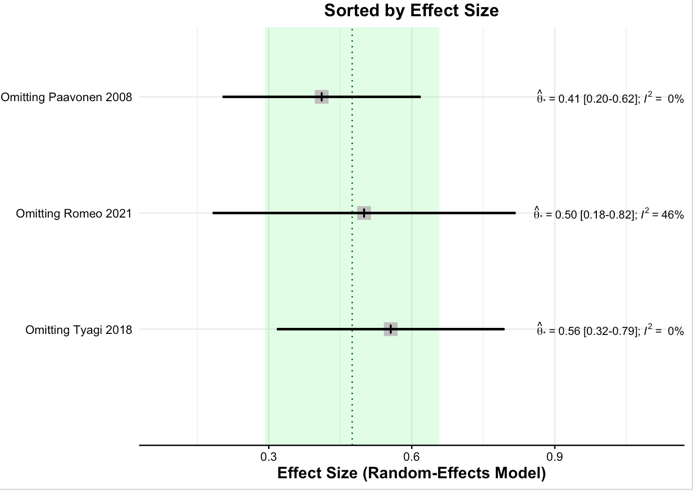 |
| --- | --- |
| 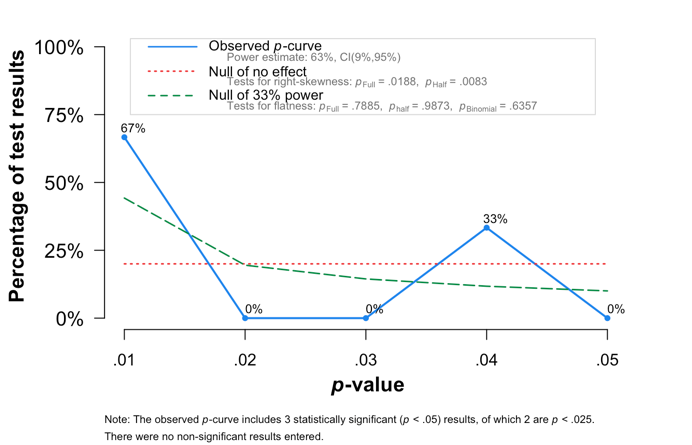 |  |

### **Figure S34. Funnel plot and influence analysis plot for Sleep-wake transition disorders**

| 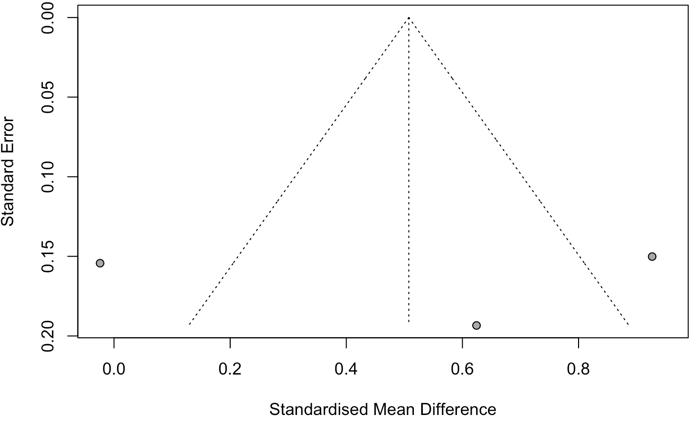 | 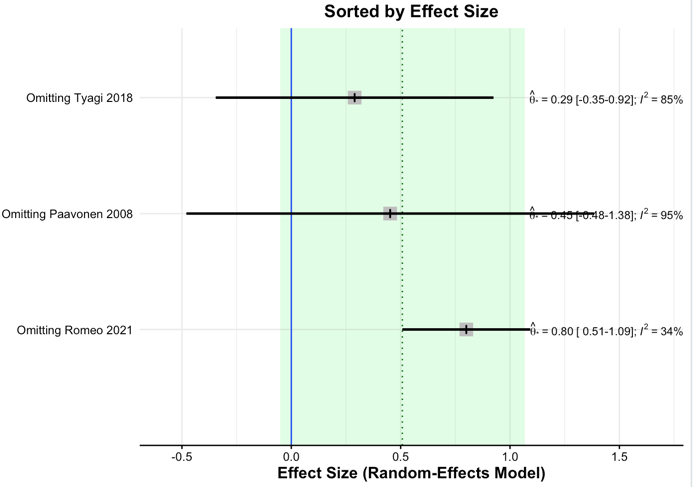 |
| --- | --- |

### **Figure S35. Funnel plot, and p-curve plot, and influence analysis plot for Total sleep problem**

| 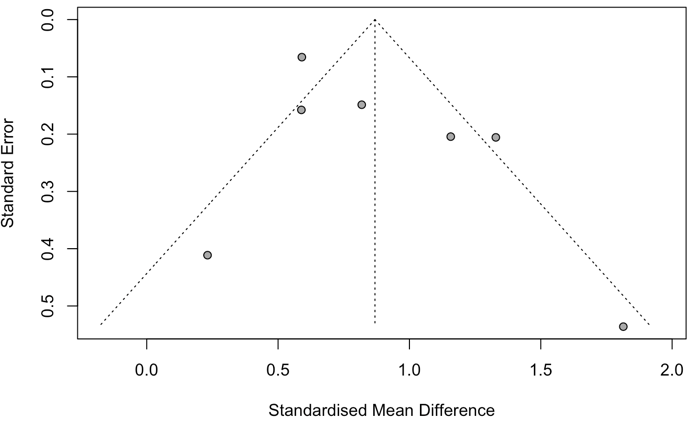 | 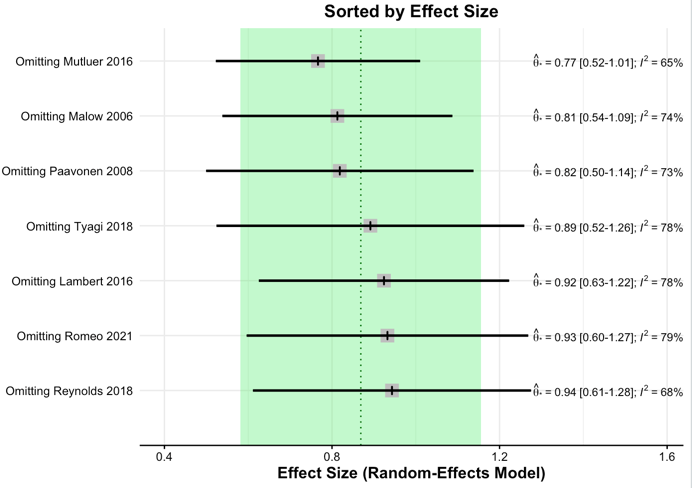 |
| --- | --- |
| 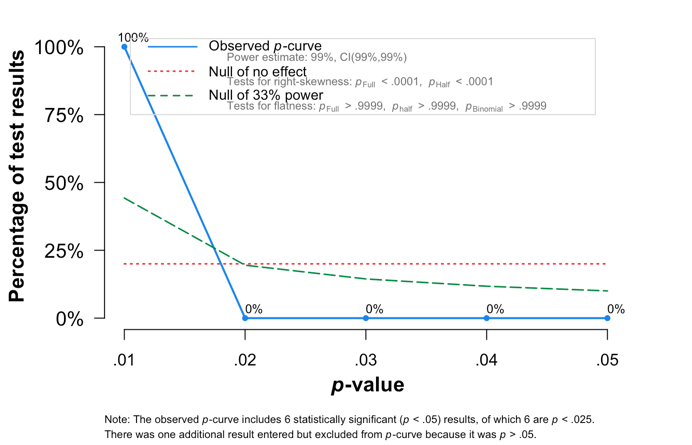 |  |
